# Scope and Synthetic Applications of the Aryl-Alcohol Oxidase from *Streptomyces hiroshimensis* (*Sh*AAO)

**DOI:** 10.1021/acs.orglett.5c03814

**Published:** 2025-10-22

**Authors:** Christian Ascaso-Alegre, Paula Cinca-Fernando, Tom L. Roberts, Pablo López-Fernández, Raquel P. Herrera, Sebastian C. Cosgrove, Patricia Ferreira, Juan Mangas-Sánchez

**Affiliations:** † Institute of Chemical Synthesis and Homogeneous Catalysis (ISQCH, Spanish National Research Council (CSIC)- 16765 University of Zaragoza, Pedro Cerbuna 12, 50009 Zaragoza, Spain; ‡ Department of Biochemistry and Molecular and Cellular Biology and Institute of Biocomputation and Physics of Complex Systems (BIFI, GBsC−CSIC Joint Unit), University of Zaragoza, Pedro Cerbuna 12, 50009 Zaragoza, Spain; § School of Chemical and Physical Sciences and Centre for Glycoscience, 4212 Keele University, Keele, Staffordshire ST5 5BG, U.K.; ∥ Department of Organic and Inorganic Chemistry, IUQOEM, 16763 University of Oviedo, Julián Clavería 8, 33006 Oviedo, Spain

## Abstract

The sustainable production of carbonyl compounds is of growing interest due to their broad academic and industrial interest. Herein, we explored the aryl-alcohol oxidase from *Streptomyces hiroshimensis* (*Sh*AAO), mapping its activity across diverse alcohols, revealing a distinct profile from other natural AAOs. Integrated into tandem organocatalytic processes, *Sh*AAO enabled the synthesis of various chiral compounds, including an intermediate for paroxetine. Gram-scale reactions delivered high yields and turnover numbers exceeding 65000. Immobilization further enhanced stability, highlighting *Sh*AAO as an efficient and industrially relevant biocatalyst.

## Graphical abstract

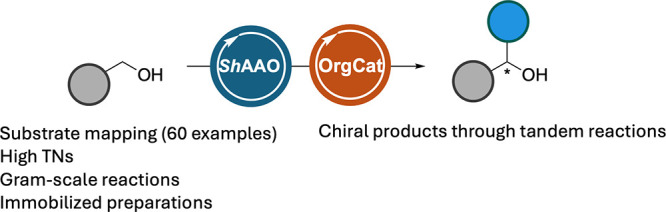

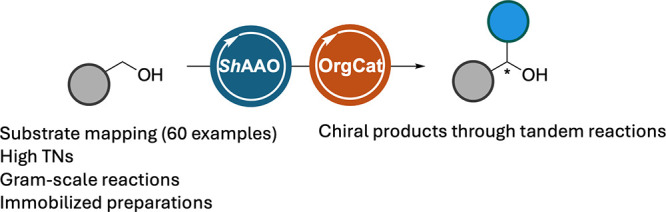

Alcohol oxidation is a central reaction in synthetic chemistry, and enzymatic approaches offer practical and environmental advantages over classical methods.^1–8^ For instance, ketoreductases (KREDs) catalyze reversible alcohol oxidation via hydride transfer to nicotinamide cofactors, but the reaction is thermodynamically unfavorable and requires cofactor recycling.^9,10^ Besides KREDs, laccases are blue copper oxidases that have also shown to be useful to mediate alcohol oxidation,^11–13^ but their low redox potential necessitates mediators, complicating large-scale use. Conversely, flavin-dependent alcohol oxidases (FAD-AOx) oxidize alcohols via hydride transfer to FAD, which is then regenerated by oxygen, producing H_2_O_2_ without requiring added cofactors since the FAD is typically tightly bound to the enzyme upon production.^14–18^ These advantages have led to an increased interest in the use of these enzymes for synthetic purposes.^19–22^ To date, a variant of the choline oxidase from *Arthrobacter chlorophenolicus* (*Ac*CO6)^23^ and different isoforms and engineered variants of the aryl-alcohol oxidase (AAO) from *Pleurotus eryngii* (*Pe*AAO) have been extensively studied.^19,20,24–27^ Their remarkable efficiency coupled with their ability to operate at high substrate loadings makes them highly promising tools for industrial biotechnology. Their substrate scope covers the oxidation of a broad range of electron-rich benzylic alcohols and aliphatic allylic alcohols such as *trans*-2-hexen-1-ol. The groups of Hollmann and Urlacher demonstrated the potential of *Pe*AAO for alcohol oxidation at scale,^19,20^ although these biocatalysts often require complex expression systems or labor-intensive refolding procedures, limiting their applicability. To address this, we have recently described two bacterial AAOs which represent a promising alternative due to their ease of production and scalability.^28,29^ In this study, we extend our investigation into the scope of bacterial AAO from *Streptomyces hiroshimensis* (*Sh*AAO) and its application to the scalable synthesis of aldehydes and chiral synthons through the construction of chemoenzymatic cascades.

To determine the scope of *Sh*AAO, we examined its activity with a panel of 60 primary and secondary alcohols in analytical scale biotransformations (Scheme 1, full list in Figure S1). Generally, *Sh*AAO displayed a strong preference for aliphatic and aromatic allylic alcohols such as cinnamyl alcohol (**1a**) or 2,4-hexadiene-1-ol (**9a**), with turnover numbers (TNs) ranging from 15000 to 35000 under these initial conditions. Cinnamyl alcohol derivatives (**2a**–**6a**) displayed similar TNs, except for the *o*-NO_2_-substituted derivative **5a**, which was obtained in 59% conversion using 2.2 μM (6185 TN) probably due to steric constraints. Notably, the propargylic alcohol **7a** was also converted, although at lower rates (TN 909). Aliphatic allylic alcohols **8a**–**12a** were also efficiently oxidized, with particularly high TNs in the case of sorbic alcohol (**9a**), *trans*-2-hexen-1-ol (**11a**) and geraniol (**12a**), with near-complete conversion to **12b** and a TN of 35656. Interestingly, the enzyme displayed no activity toward structurally similar compounds such as **47a** or **49a** (Figure S1). Good performances were found with aryl alcohol derivatives **13a**–**31a**. The fungal AAO scope includes electron-rich aryl alcohols **22a**–**25a** with TNs, in this case, ranging from 636 for the *m*-OMe derivative to a remarkable TN of 12371 for **24a**. Concerning halogenated compounds **14a**–**18a**, a preference toward *para*-substituted derivatives was found, with conversion decreasing from 78% for *p*-bromobenzyl alcohol **15a**, to 31% for *m*-bromobenzyl alcohol **16a** and 0% for *o*-bromobenzyl alcohol **17a**. Remarkable differences were found in the *p*-F, *p*-Cl, and *p*-Br series. While *Sh*AAO demonstrated high activity toward **15a**, a 10- and 20-fold decrease in TN was found for the *p*-chlorobenzyl and the *p*-fluorobenzyl derivatives **14a** and **18a**, respectively, suggesting a strong influence of the active site architecture in specificity and catalytic efficiency. *Sh*AAO was also studied toward nitro aromatics **19a**–**20a**. Similarly, a preference toward the *para*-derivative was found with moderate activities (3820 and 6731 TNs for the *meta* and *para* compounds, respectively). The presence of hydroxy and amino groups in the aromatic ring was evaluated, finding activity toward phenol derivative **25a** (41% conversion) but not aniline **26a**. Heteroaromatic alcohols **27a**–**31a** were also studied. Furan derivatives, which are promising building blocks for bioplastics,^30–32^ were well tolerated with a remarkable TN of 16000 for HMF **28a**. Unlike *Pe*AAO,^20^ no activity was found toward **27a**. Interestingly, while 3-pyridinemethanol **31a** was oxidized in 29% conversion (TN 5726), only traces of the product were detected when 2-pyridinemethanol **30a** was used. We also investigated the scope toward nonactivated alcohols. *Sh*AAO showed activity toward linear aliphatic alcohols starting from 1-pentanol **33a** (TN 364), with activity increasing up to 1-nonanol **37a** (40% conversion, TN 3636). For longer-chain alcohols, activity declined and became undetectable beyond 1-heptadecanol **40a**. To the best of our knowledge, *Sh*AAO represents the first naturally occurring AAO capable of oxidizing alkanols. We also tested a comprehensive list of primary and secondary alcohols that were not accepted by *Sh*AAO (selected examples in Scheme 1 and full list in Figure S1). Given the similarity between the active sites of *Sh*AAO and *Pe*AAO,^18^ steric hindrance likely prevents secondary alcohols from adopting a catalytically competent orientation for hydride transfer, which results in their oxidation being significantly impaired or totally restricted. Moreover, these results show that *Sh*AAO possesses a substrate scope comparable to that of *Ac*CO6, despite the latter not belonging to the AAO family. However, previous kinetic data,^28^ together with the high TNs obtained for allylic, benzylic, and cinnamyl alcohol derivatives, indicate that *Sh*AAO exhibits higher catalytic activity.

**Scheme 1:**
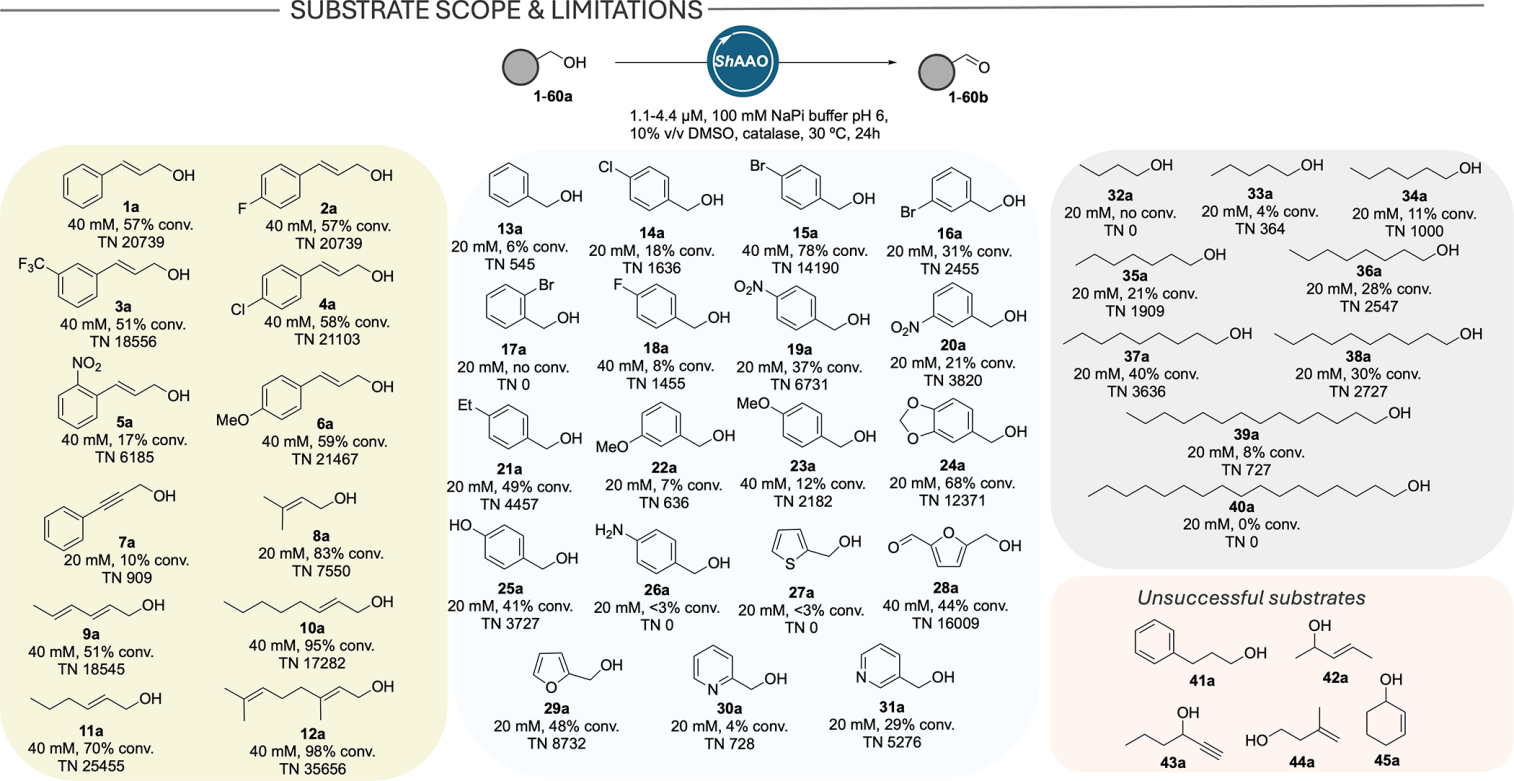
Alcohol Oxidation Scope of *Sh*AAO^a^

a Conditions: 20–40 mM substrate concentration (1 mL reaction volume), 10% v/v DMSO, 1.1–4.4 μM *Sh*AAO, catalase (2000 U/mL) in 100 mM sodium phosphate (NaPi) pH 6 at 30°C for 24 h. Conversions determined by ^1^H-NMR over the reaction crudes. TNs defined as mmol of product formed/mmol of catalyst. Representative unsuccessful substrates are shown. Full substrate list in the SI.

Artificial cascades combine consecutive reactions to produce complex molecules without intermediate purification, enabling rapid conversion of unstable or toxic intermediates like aldehydes while improving safety and efficiency.^33–41^ Following on our experience,^42,43^ we tested the use of *Sh*AOO in the synthesis of chiral synthons and other relevant compounds via one-pot cascade processes combining the biooxidation process with organocatalytic reactions and the Wittig olefination (Scheme 2). Initially, since aeration is crucial for oxygen-dependent biocatalysis,^44,45^ the effect of vessel size was tested in the oxidation of **1a**, **9a**, and **12a**. Larger 10 mL vials improved conversions compared with 2 mL tubes, likely from enhanced oxygen transfer (Scheme S2). Also, with the potential of DMSO to interfere with organocatalytic transformations, we assessed the oxidation reaction under DMSO-free conditions, finding minimal impact (Scheme S3).

**Scheme 2:**
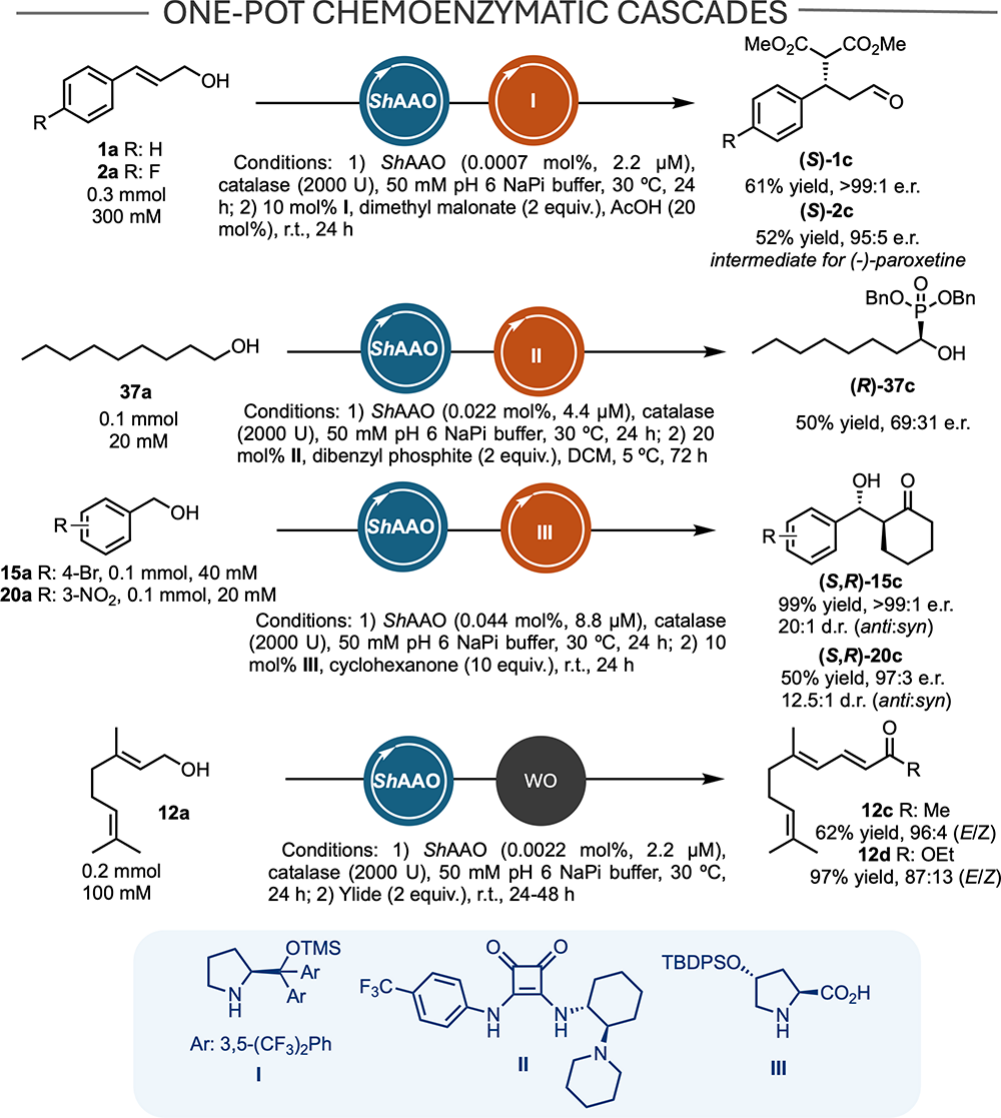
Synthetic Applications of *Sh*AAO to Access Chiral and Other Synthetically Relevant Synthons^a^

a Yields are for the isolated product after column chromatography. Enantiomeric ratios (e.r.) were determined by HPLC on a chiral stationary phase. WO: Wittig Olefination.

We started by investigating a one-pot process combining aerobic oxidation with the organocatalyzed asymmetric conjugate addition of dimethyl malonate via iminium ion activation. For the organocatalytic step, we evaluated the Hayashi-Jørgensen catalyst **I** ^46,47^ starting from **1a**, obtaining (*S*)-**1c** in 61% isolated yield and an e.r. > 99:1 using 2.2 μM *Sh*AAO in the initial oxidation step. Since the 4-fluoro derivative (*S*)-**2c** is a synthetic precursor of (−)-paroxetine,^48^ the cascade was also performed starting from **2a**, yielding (*S*)-**2c** in 52% yield and 95:5 e.r. *Sh*AAO displays a distinct scope to fungal oxidases, as these are not capable of oxidizing saturated aliphatic alcohols. In our previous report on the synthesis of chiral α-hydroxyphosphonates, access to saturated compounds was restricted due to limitations in the scope of FX9.^43^ With this in mind, nonanol **37a** was chosen as the substrate, and optimal conditions previously described were applied.^43^ Using 4.4 μM *Sh*AAO and squaramide **II** as catalysts, product (*R*)-**37c** was obtained in 50% yield with an er of 69:31. We also applied this combined approach with catalysts operating via enamine catalysis to access chiral hydroxy ketones. Among the different species described for aldol reactions in water,^49^ we selected proline-derivative **III** ^50^ and Singh’s catalyst^51^ due to their synthetic ease and excellent potential in the addition of cyclohexanone to benzaldehyde derivatives. Starting from **20a**, catalyst **III** demonstrated superior performance, isolating (*S*,*R*)-**20c** in 50% yield, a 97:3 er, and a dr of 12.5:1. We also evaluated 4-Br benzyl alcohol **15a**, obtaining (*S*,*R*)-**15c** after the tandem process in 99% isolated yield, >99:1 e.r., and 20:1 d.r.

Finally, the biooxidation process was coupled with a Wittig reaction, establishing a cascade analogous to the one recently described by Wahart et al.^52^ We chose geraniol **12a** (100 mM) and two phosphorus ylides, obtaining **12c** and **12d** in 62% and 97% yields, respectively. The use of *Sh*AAO enabled the process to be performed at higher substrate concentrations with lower catalyst loadings than those required in previously reported cascades.

We also evaluated *Sh*AAO for preparative-scale reactions using **1a** as the substrate (Table S1). Initially, 1 mL biotransformations were conducted in 10 mL reaction vials at increasing concentrations of **1a** with 1.1 μM enzyme and 2000 U/mL catalase, achieving 48% conversion at 120 mM (16 g/L) of **1a**. Increasing catalyst loading to 4.4 μM resulted in complete conversion, with 92% conversion at 240 mM (32 g/L) and 48% conversion at 500 mM (69 g/L), corresponding to a TN of 51349 and a TOF of 349 min^–1^ (Table S2, Figure S53). Considering the importance of oxygen concentration and transfer rate,^44,53^ we investigated the effect of catalase concentration, reaction vessel volume (25 mL), and use of O_2_ atmosphere, although no significant improvement was found. However, stepwise addition of catalase (2 × 8000 U/mL) slightly increased conversion to 57% (TN 64390), with no further gains observed using more catalase. Under these conditions, we performed a gram-scale process in a 2 × 10 mL reaction volume using 100 mL baffled flasks for improved aeration. After 6 h, we observed a 42% conversion and obtained 422 mg of **2a** after column chromatography (33% isolated yield and productivity of 168 g of product/g of enzyme). We then switched to cell-free extracts (CFE). Based on enzyme expression and purification data, we estimated that 4.3 μM *Sh*AAO corresponds to 2 mg/mL CFE. The process using CFE led to a 61% conversion and a 53% yield (17.1 g product/g CFE). This suggests that the stabilizing effect of CFE has a positive effect on the catalyst performance. To maximize yield, we finally increased CFE concentration to 4 mg/mL, achieving 94% and 91% conversion after 6 h. The combined reaction crudes were purified obtaining **2a** in 75% yield (0.96 g, 12 g product/g CFE).

To improve and demonstrate the synthetic utility of *Sh*AAO as a reusable biocatalyst, enzyme immobilization was explored. A range of affinity resins (EziG Amber, Coral, and Opal), an aminoglutaraldehyde resin (ECR8309F), and an epoxy resin (EMC7025) were tested.^54–58^ Immobilization at r.t. was unsuccessful due to enzyme precipitation, which was accelerated in the presence of the resins. Instead, immobilization was done at 4 °C. Initial conversion on Opal and Amber appeared most promising at 86% and 55% conversion, respectively (Figure 1); however, retained activity across two subsequent reaction cycles showed a loss in activity (Figure 2). It was evident that ECR8309F was the most suitable carrier for reuse and subsequent scale up experiments, with no demonstratable loss of activity observed across three ninety-minute reactions.

**Figure 1:**
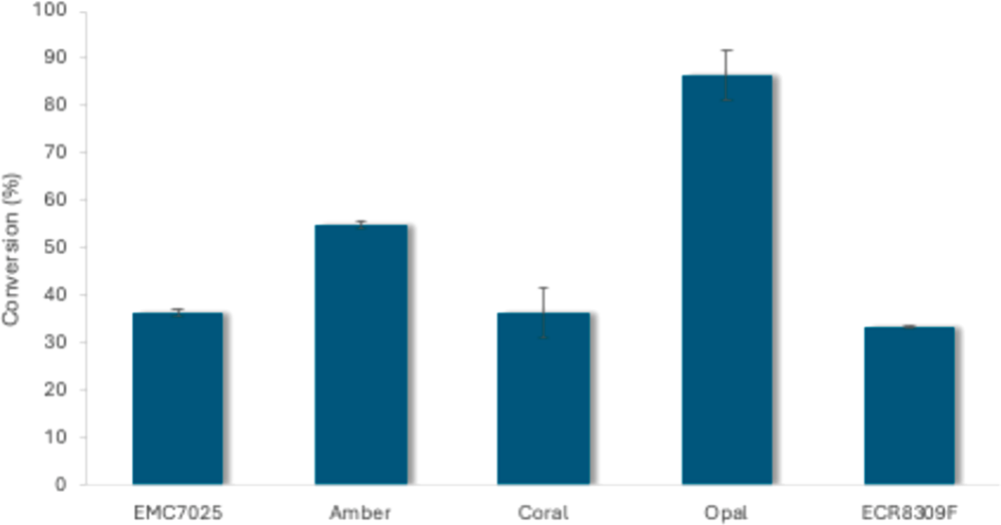
Initial conversion of **1a** (40 mM) using immobilized preparations of *Sh*AAO. Reactions carried out using 50 mg carrier, 2000 U/mL catalase in 1 mL reaction buffer (100 mM KPi, pH 6, 10% DMSO). Error bars are SEM, *n* = 2.

**Figure 2:**
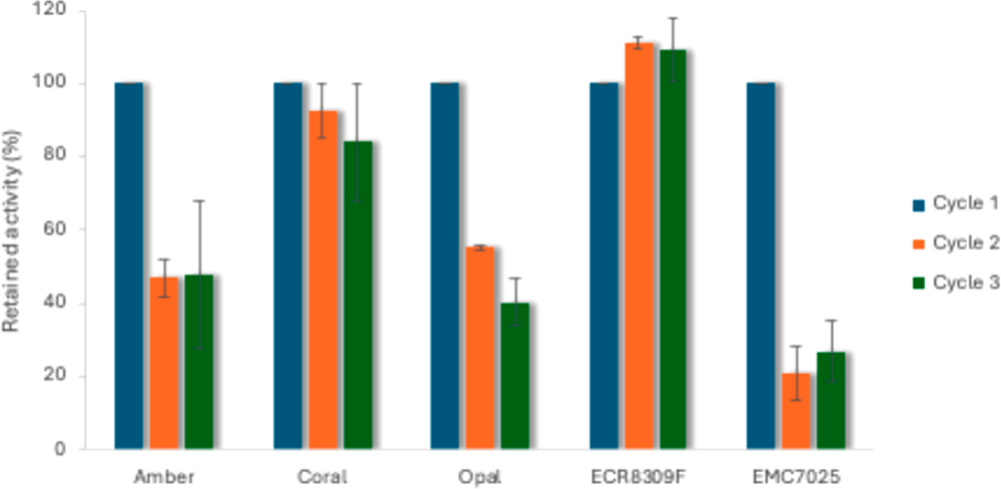
Retained activity studies of **1a** (40 mM) using immobilized preparations of *Sh*AAO. Reactions carried out using 50 mg carrier, 2000 U/mL catalase in 1 mL reaction buffer (100 mM KPi, pH 6, 10% DMSO). Error bars are SEM, *n* = 2.

The use of baffled flasks with high concentration (500 mM **1a**)/low volume (<10 mL) reactions was attempted using immobilized enzyme; however, issues with separation of the immobilizate from the solution led to poor conversions (12%). Falcon tubes were instead attempted, but presumed low oxygen availability also led to low conversion (13%). In subsequent reactions conducted in 100 mL Erlenmeyer flasks, the results were more promising at 29% conversion. Higher catalase concentrations caused difficulties in separation of the carrier between subsequent reactions, so subsequent reactions were instead completed at a lower 40 mM concentration of **1a**, with a 25 mL reaction volume in a 50 mL Falcon tube. Initial conversions showed an 81% conversion to **2a**. The immobilized biocatalyst retained full activity for a second cycle (86%) before a loss in activity during cycle 3 (44%). The loss in activity could be due to several deactivation methods, including enzyme unfolding. The nature of the amino resin also means leaching via imine hydrolysis could be occurring to a small extent. Despite this drop in activity, when combined, this amounts to a TN of 67267 and permitted the same sample of enzyme to be isolated and reused three times (Table S7).

In summary, we demonstrated the broad substrate tolerance, high catalytic efficiency, and scalable applicability of *Sh*AAO in the selective oxidation of primary alcohols. *Sh*AAO exhibited remarkable TNs (up to >50000) for allylic and benzylic alcohols and, notably, is the first naturally occurring AAO reported to oxidize alkanols, expanding the known substrate scope of this enzyme class. The biooxidation was successfully integrated into multiple chemoenzymatic cascade reactions, enabling the efficient synthesis of valuable chiral compounds. Importantly, *Sh*AAO also demonstrated a strong potential for preparative-scale applications. Gram-scale oxidations were achieved with high productivity using purified enzyme and CFEs. Although *Pe*AAO exhibits higher TN values for certain substrates,^20,59^ *Sh*AAO offers a more practical alternative thanks to its simple production and effective use in semipurified form. We have also shown increased stability upon immobilization, which permitted reuse for three subsequent reaction cycles, achieving a total TN > 65000. The unique properties of *Sh*AAO highlight its utility as a versatile biocatalyst for sustainable synthetic applications.

## Supplementary Material



## Data Availability

The data underlying this study are available in the published article and its Supporting Information.

## Notes

Full details of the materials, methods, additional experimental details, and data including NMR spectra and HPLC traces that support the findings of this study (PDF)
