# Supplementary material for: Scope and Synthetic Applications of the Aryl-Alcohol Oxidase from Streptomyces hiroshimensis (ShAAO)
Source: Org Lett. 2025 Oct 22;27(43):12086–91. doi: 10.1021/acs.orglett.5c03814 (PMC12584139; doi:10.1021/acs.orglett.5c03814)
Supplement: Supplementary file 1 [file ol5c03814_si_001.pdf]

SUPPLEMENTARY INFORMATION

Scope and synthetic applications of the aryl-alcohol oxidase from *Streptomyces hiroshimensis* (*Sh*AAO)

Christian Ascaso-Alegre,[a] Paula Cinca-Fernando,[b] Tom Roberts,[c] Pablo López-Fernández,[d] Raquel P. Herrera,[a] Sebastian C. Cosgrove,[c] Patricia Ferreira [b*] and Juan Mangas-Sánchez [d*]

*Corresponding authors: ferreira@unizar.es; mangasjuan@uniovi.es

Table of Contents

S1. GENERAL CONSIDERATIONS 2

S2. SUBSTRATE LIST 3

S3. GENE EXPRESSION AND PROTEIN PURIFICATION 4

S4. PREPARATION OF *Sh*AAO CELL-FREE-EXTRACT (CFE) 5

S5. PROTEIN CONCENTRATION ASSAY. 5

S6. GENERAL PROCEDURE FOR THE SYNTHESIS OF CINNAMYL ALCOHOL DERIVATIVES **2a**-**6a** .5

S7. SUBSTRATE SCOPE INVESTIGATION. GENERAL PROCEDURE. 5

S8. ^{1}H-NMR SPECTRA OF BIOXIDATION REACTIONS CATALYZED BY *Sh*AAO. 7

S9. SYNTHESIS OF CATALYST **III** AND SINGH'S CATALYST (**IV**) 26

S10. INITIAL OPTIMIZATION OF THE BIOOXIDATION STEP FOR CHEMOENZYMATIC CASCADES. 29

S11. GENERAL PROCEDURE FOR THE *ONE-POT* CASCADE PROCESS FOR THE SYNTHESIS OF CHIRAL HYDROXY KETONES (*S*,*R*)-**15c** AND (*S*,*R*)-**20c**. 30

S12. GENERAL PROCEDURE FOR THE *ONE-POT* CASCADE PROCESS FOR THE SYNTHESIS OF CHIRAL 1,2-HYDROXY PHOSPHONATE (*R*)-**37c.** 31

S13. GENERAL PROCEDURE FOR THE *ONE-POT* CASCADE PROCESS COMBINING *Sh*AAO-MEDIATED OXIDATION AND ORGANOCATALYTIC ASYMMETRIC MICHAEL ADDITIONS. 33

S14. GENERAL PROCEDURE FOR THE *ONE-POT* CASCADE PROCESS COMBINING *Sh*AAO-MEDIATED OXIDATION AND WITTIG OLEFINATION. 35

S15. CHIRAL HPLC ANALYSIS OF COMPOUNDS **15c**, **20c** AND **37c** 37

S16. PREPARATIVE-SCALE BIOTRANSFORMATIONS. 42

S17. ENZYME IMMOBILISATION 46

S1. General considerations

All starting products were chemically synthesised (see below synthetic procedures) or purchased from Sigma-Aldrich or BLD Pharmatech. Catalase from bovine liver C9322 was obtained from Sigma Aldrich. Reactions were monitored by thin-layer chromatography (TLC) using aluminum plates coated with silica gel and a fluorescent indicator (60 F254, 0.2 mm). Compounds were visualized at 254 nm using UV light. Products **1b**, (*S*)-**1c**, (*S*)-**2c**, (*R*)-**37c**, (*S*,*R*)-**15c**, (*S*,*R*)-**20c**, **12c** and **12d** were isolated by column chromatography using silica gel (0.06–0.2 mm) as the stationary phase and commercial mixtures of *n*-hexane and EtOAc as the eluent. Conversions of biocatalytic reactions were determined by ^{1}H NMR over the reaction crude. The performance of the *Sh*AAO enzyme was evaluated by turnover number (TN), calculated as the ratio between the moles of product formed and the moles of enzyme used.

Catalysts **II** and **III** and phosphorane **c** are commercially available. Phosphorane **d** was prepared as reported in the literature.[1]

The ^{1}H NMR and ^{13}C{^{1}H}-APT NMR spectra of reagents and products were recorded at 300 MHz (Bruker ARX300 spectrometer) or 400 MHz (Bruker AV400 spectrometer), in chloroform-d ($CDCl_3$) as deuterated solvent. Chemical shifts were reported in the δ scale relative to residual $CHCl_3$ (7.26 ppm) for ^{1}H NMR and the central line of $CDCl_3$ (77.16 ppm) for ^{13}C{1H}-APT NMR.

The chiral HPLC analysis of products (*S*)-**1c**, (*S*)-**2c**, (*S*,*R*)-**15c**, (*S*,*R*)-**20c** and (*R*)-**37c** was performed in a Waters 600 equipment, using a Daicel ChiralPak IC/IA or a Phenomenex i-Amylose-1column as stationary phase and mixtures of commercial *n*-hexane/isopropyl alcohol or *n*-hexane/ethyl acetate as eluent.

The spectral data of products **1c**,[2] **2c**,[3] **12c**,[4] **12d**,[5] **15c**,[6] **20c**,[7] and organocatalysts **III**[8] y **IV**[9] are consistent with values previously reported in the literature.

---

[1] J. Vicente, M. T. Chicote, I. Saura-Llamas, *J. Chem. Educ.* **1993**, *70*, 163−164.
[2] S. Brandau, A. Landa, J. Franzén, M. Marigo, K. A. Jørgensen, *Angew. Chem. Int. Ed.* **2006**, *45*, 4305—4309.
[3] O. V. Maltsev, A. S. Kucherenko, S. G. Zlotin, *Eur. J. Org. Chem.* **2009**, *2009*, 5134−5137.
[4] S. Narayanasamy, J. Sun, R. E. Pavlovicz, A. Eroglu, C. E. Rush, B. D. Sunkel, C. Li, E. H. Harrison, R. W. Curley, *J. Lipid Res.* **2017**, *58*, 1021−1029.
[5] D. M. Cermak, D. F. Wiemer, K. Lewis, R. J. Hohl, *Bioorg. Med. Chem.* **2000**, *8*, 2729−2737.
[6] A. Yanagisawa, Y. Nakatsuka, K. Asakawa, M. Wadamoto, H. Kageyama, H. Yamamoto, *Bull. Chem. Soc. Jpn.* **2001**, *74*, 1477−1484.
[7] B. Rodríguez, A. Bruckmann, C. Bolm, *Chem. Eur. J.* **2007**, *13*, 4710−4722.
[8] S. M. Opalka, J. L. Steinbacher, B. A. Lambiris, D. T. McQuade, *J. Org. Chem.* **2011**, *76*, 6503–6517.
[9] A. Berkessel, W. Harnying, N. Duangdee, J.-M. Neudörfl, H. Gröger, *Org. Process Res. Dev.* **2012**, *16*, 123–128.

S2. Substrate list

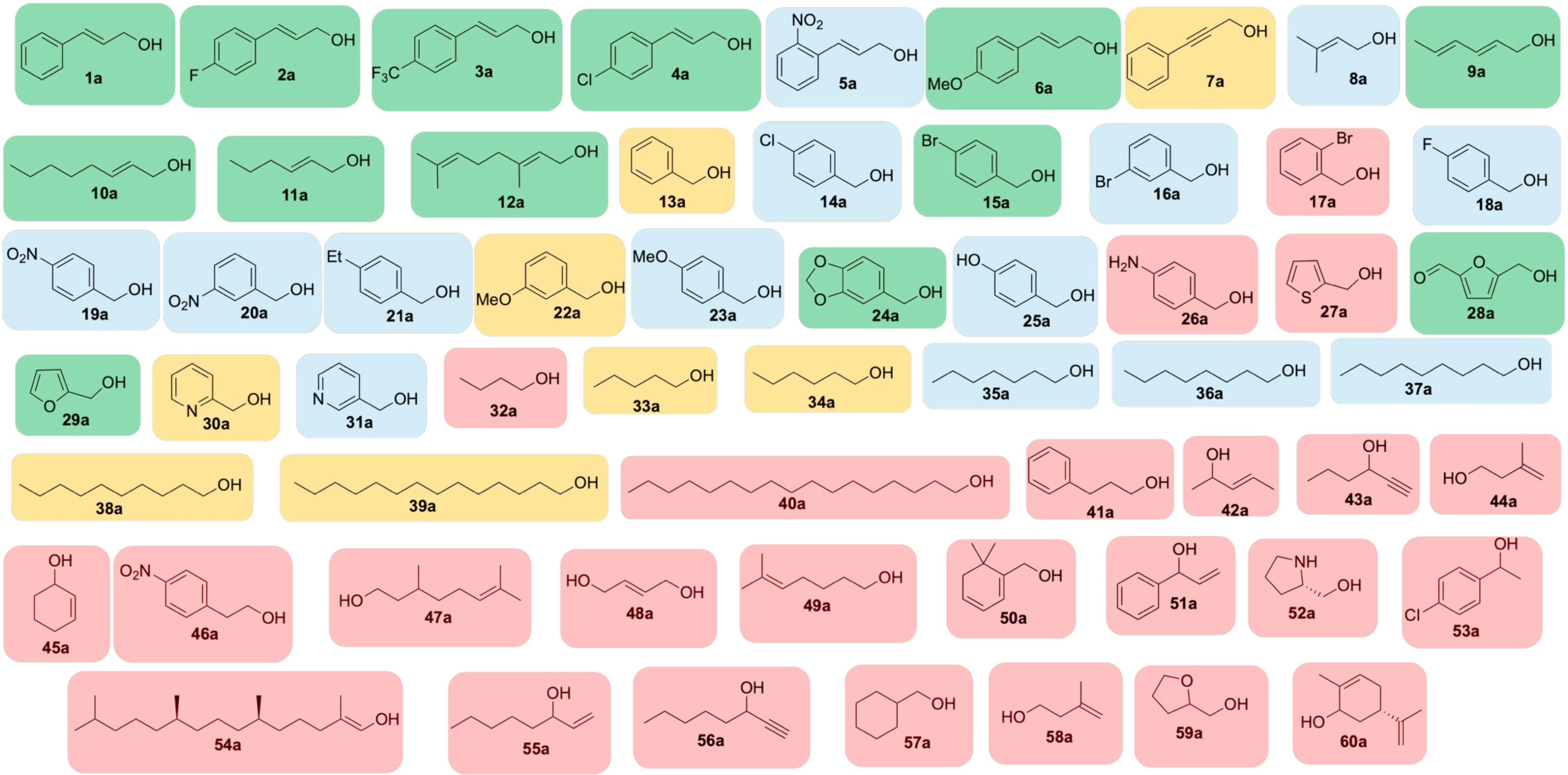

Figure S1. Full list of alcohols **1-60a** screened in this contribution. Red: no activity. Pale yellow: TN 0-1000. Blue: TN 1000-10000. Green: TN >10000. Turnover numbers (TN) calculated as the ratio between the moles of product formed and the moles of enzyme used.

S3. Gene expression and protein purification

Recombinant *Sh*AAO was heterogously expressed in *Escherichia coli* C41 (DE3) as a protein with a cleavable N-terminal His_6-tag, using the pET28a(+) expression vector. For protein production, a 50 mL preinoculum was prepared in LB medium supplemented with 30 µg/mL kanamycin and incubated overnight at 37 °C and 180 rpm. Overexpression was carried out in 2 L shaking flasks containing 1 L of TB medium supplemented with 30 µg/mL kanamycin and 8 mg/L riboflavin. The main cultures were inoculated with 5% (v/v) of the preinoculum and grown at 37 °C and 180 rpm. Upon reaching an OD_{600} ~1.5, protein expression was induced with 0.1 mM IPTG. After induction, the cultures were incubated for 72 hours at 37 °C and 180 rpm. Cells were harvested by centrifugation at 6000 × *g* for 15 minutes at 4 °C. The resulting cell pellets were washed and resuspended in 50 mM sodium phosphate, pH 7.4, followed by centrifugation at 6000 × *g* for 15 minutes at 4 °C. The final pellets were stored at −80 °C until the purification.

For purification, bacterial pellets from 1 L of culture were resuspended in 10 mL of 50 mM sodium phosphate, pH 7.4, supplemented with a protease inhibitor cocktail (cOmplete EDTA-free Protease Inhibitor Tablets from Roche). Cells were lysed by sonication, and the lysate was clarified by centrifugation at 8000 × *g* for 20 minutes at 4 °C. The soluble fraction was adjusted to 10 mM imidazole and 100 mM NaCl, and incubated with 4 mL of Ni^{2+}-charged IMAC Sepharose resin (GE Healthcare), previously equilibrated with the same binding buffer. After 2 hours of incubation at 4 °C on a bidirectional orbital shaker, the mixture was loaded onto a gravity-flow column and washed with five column volumes of binding buffer. The bound protein was eluted with 100 mM imidazole in 50 mM sodium phosphate, pH 7.4. Fractions showing an A_{280}/A_{450} ratio of ~10, corresponding to *Sh*AAO, were pooled and dialyzed using a PD-10 desalting column in 50 mM sodium phosphate, pH 6.0. Protein purity was confirmed by SDS-PAGE (Figure S2) and was stored at −80 °C for further studies.

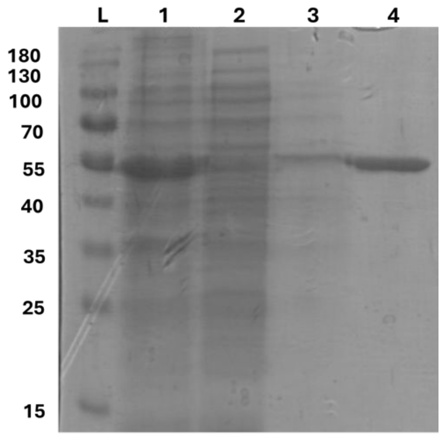

Figure S2. SDS-PAGE analysis of *Sh*AAO enzyme purification. L: Molecular weight marker; 1: Cell lysate; 2: Unbound protein; 3: Column wash; 4: Eluted protein.

S4. Preparation of *Sh*AAO cell-free-extract (CFE)

Following the gene expression protocol (Section S3), bacterial pellets from 1 L of culture were resuspended in 10 mL of 50 mM sodium phosphate pH 6.0, supplemented with a protease inhibitor cocktail (cOmplete EDTA-free Protease Inhibitor Tablets from Roche), sonicated, and then centrifuged at 4 °C for 20 minutes at 8000 × g. Subsequently, 20 mL of cell-free extract was transferred to a 50 mL falcon tube. The falcon tube was snap frozen in liquid nitrogen prior to freeze-drying for 1-2 days until powdery. The freeze-dried cell-free lysate (CFE) was stored at -20 °C prior to use.

S5. Protein concentration assay.

The protein concentration was determined using a UV/Visible spectrophotometer and its molar absorptivity coefficient (ε_{456} = 10914 M^{-1} cm^{-1} in 50 mM sodium phosphate pH 6.0 at 25 ºC).

S6. General procedure for the synthesis of cinnamyl alcohol derivatives 2a-6a

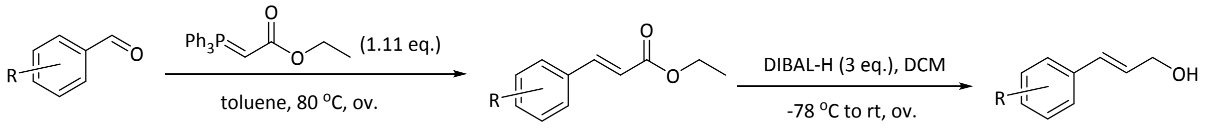

Cinnamyl alcohol derivatives **2-6a** were prepared following a telescopic protocol from benzaldehyde derivatives **2-6e**. A solution of the corresponding aldehyde **2-6e** (2 mmol, 1 equiv.) in toluene (3 mL) was treated with ethyl(triphenylphosphoranylidene)acetate (2.22 mmol, 1.11 equiv.), and the reaction mixture was stirred at 80 °C overnight using an oil bath. The resulting ethyl ester intermediates **2-6f** were purified by column chromatography using a mixture of *n*-hexane and ethyl acetate (9:1). Once purified, the corresponding ethyl esters **2-6f** (1.5 mmol, 1 eq.) were dissolved in dry dichloromethane (15 mL) under an inert atmosphere at −78 °C, and DIBAL-H (4.5 mmol, 3 equiv.) was added. The reaction was allowed to reach room temperature and stirred overnight. After this time, the reaction was diluted with diethyl ether, and the mixture was cooled to 0 ºC. Then, water and a 15% aqueous solution of NaOH were slowly added. The mixture was allowed to reach room temperature and stirred for 15 minutes. Finally, the mixture was dried over $MgSO_4$, stirred for another 15 minutes, and filtered to remove salts. Once the solvent was removed under reduced pressure, the crude product was purified by column chromatography using a mixture of *n*-hexane and ethyl acetate (8:2) to afford the cinnamyl alcohol derivatives **2-6a** in excellent yields (90-94%).

S7. Substrate scope investigation. General procedure.

To evaluate *Sh*AAO in preparative synthetic chemistry, small-scale biotransformations were conducted using substrate concentrations ranging from 20 to 100 mM and an enzyme concentration of 1.1 or 2.2 μM in a total volume of 1 mL in 50 mM NaPi pH 6 buffer with 10% v/v of DMSO. Catalase was also added to deplete the hydrogen peroxide generated during the reaction.

In an Eppendorf tube, a stock solution of the corresponding alcohol **1a-60a** (100 μL, 0.2-1.0 M) in DMSO, 50 mM NaPi pH 6.0 buffer (900 μL), catalase (1 mg, 2000–5000 U/mg of catalase) and *Sh*AAO (1.1-4.4 μM final concentration) were added to a total reaction volume of 1 mL. The reaction was shaken for 24 h at 160 rpm and 30 °C in an orbital shaker. Then, the mixture was extracted with $CDCl_3$ (3 × 0.5 mL), organic layers were combined, dried over $MgSO_4$, filtered, and conversion to the corresponding aldehyde was measured by ^{1}H-NMR in a 300 MHz NMR (Section S8).

Substrate	Concentration (mM)	*Sh*AAO (μM)	Conversion (%)	Method	TN
1a	40	1.1	57	NMR	20739
2a	40	1.1	57	NMR	20739
3a	40	1.1	51	NMR	18556
4a	40	1.1	58	NMR	21103
5a	40	1.1	17	NMR	6185
6a	40	1.1	59	NMR	21467
7a	20	1.1	10	NMR	909
8a	20	1.1	83	NMR	7550
9a	40	1.1	51	NMR	18545
10a	40	1.1	95	NMR	17282
11a	40	1.1	70 (GC) 68 (NMR)	NMR & GC	25455
12a	40	1.1	98	NMR	35656
13a	20	2.2	6	NMR	545
14a	20	2.2	18	NMR	1636
15a	40	2.2	78	NMR	14190
16a	20	2.2	31	NMR	2455
18a	40	2.2	8	NMR	1455
19a	20	2.2	37	NMR	6731
20a	20	2.2	21	NMR	3820
21a	20	2.2	49	NMR	4457
22a	20	2.2	7	NMR	636
23a	20	2.2	12	NMR	2182
24a	20	2.2	68	NMR	12371
25a	20	2.2	41	NMR	3727
28a	40	2.2	44	NMR	16009
29a	20	2.2	48	NMR	8732
30a	20	2.2	4	NMR	728
31a	20	2.2	29	NMR	5276
33a	20	2.2	4	NMR	364
34a	20	2.2	11	NMR	1000
35a	20	2.2	21	NMR	1909

36a	20	2.2	38	NMR	3455
36a	20	4.4	74	NMR	3364
37a	20	2.2	40	NMR	3636
38a	20	2.2	30	NMR	2727
39a	20	2.2	8	NMR	727

Table S1. Alcohol oxidation scope of *Sh*AAO, summary of conditions for successful substrates. Conversions determined by ^{1}H-NMR or GC over the reaction crudes. TNs defined as mmol of product formed/mmol of catalyst.

S8. ^{1}H-NMR spectra of bioxidation reactions catalyzed by *Sh*AAO.

The activity of *Sh*AAO was determined via NMR spectroscopy following extraction with $CDCl_3$. Catalyst performance was assessed using the turnover number (TN), calculated as the ratio of moles of product formed to moles of enzyme used. Reaction conversions were determined by ^{1}H NMR spectroscopy. Conversion values were quantified based on the integration of characteristic proton signals: the aldehydic proton of the product (1H; 9-10 ppm) and the CH_2 protons adjacent to the alcohol moiety of the substrate (2H; 4-4.5 ppm for allylic alcohols, 4.5-5 ppm for benzyl alcohols and 3.5-4 ppm for aliphatic alcohols). The corresponding substrate is displayed in each of the figures.

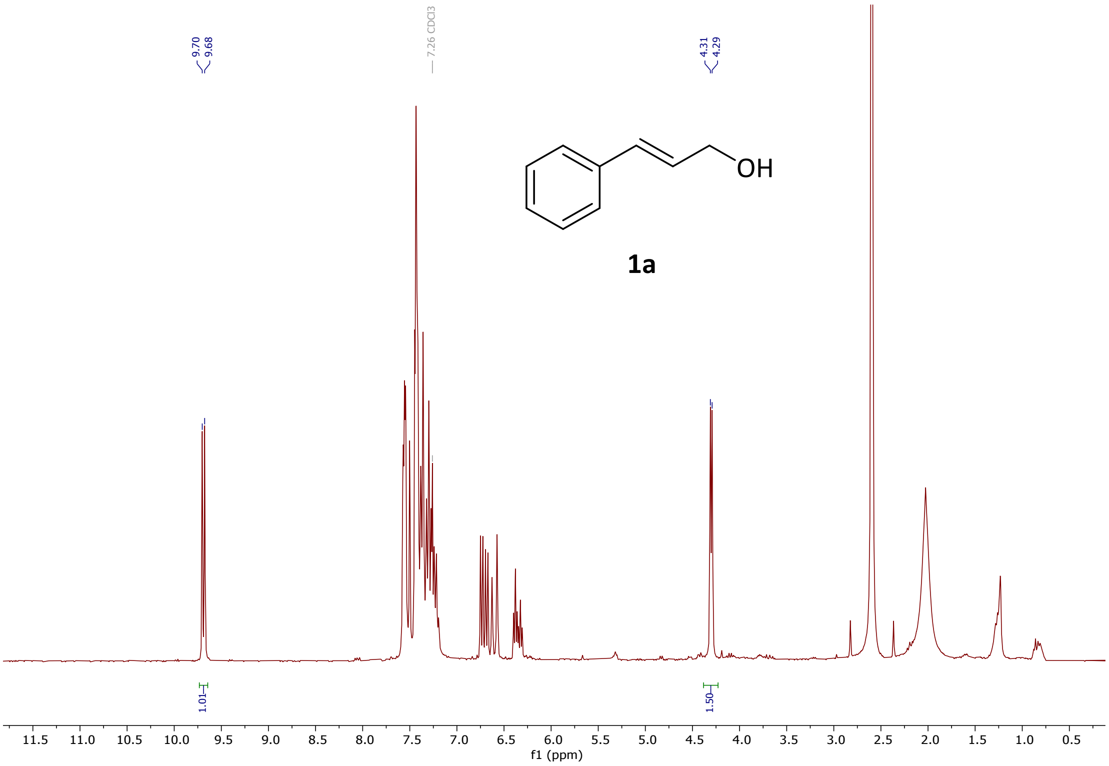

Figure S3. ^{1}H NMR spectrum of the reaction crude in the *Sh*AAO-catalyzed aerobic oxidation of **1a**. (400 Hz, $CDCl_3$).

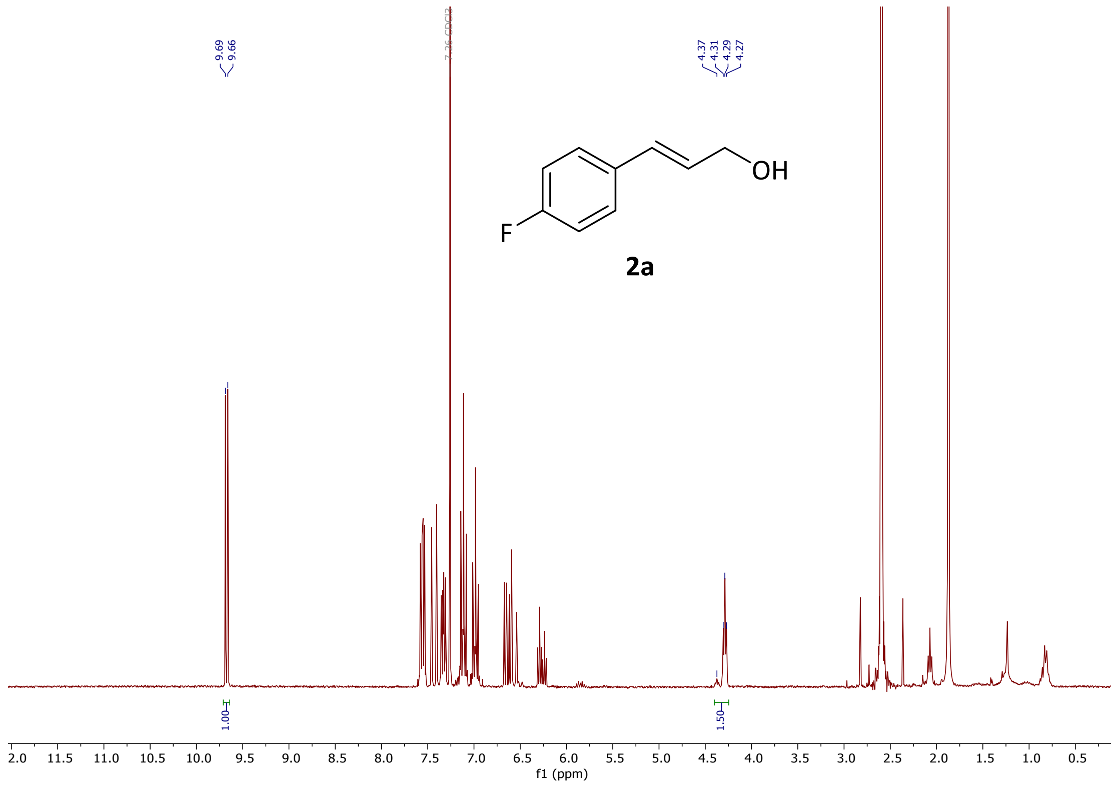

Figure S4. ^{1}H NMR spectrum of the reaction crude in the *Sh*AAO-catalyzed aerobic oxidation of **2a**. (300 Hz, $CDCl_3$).

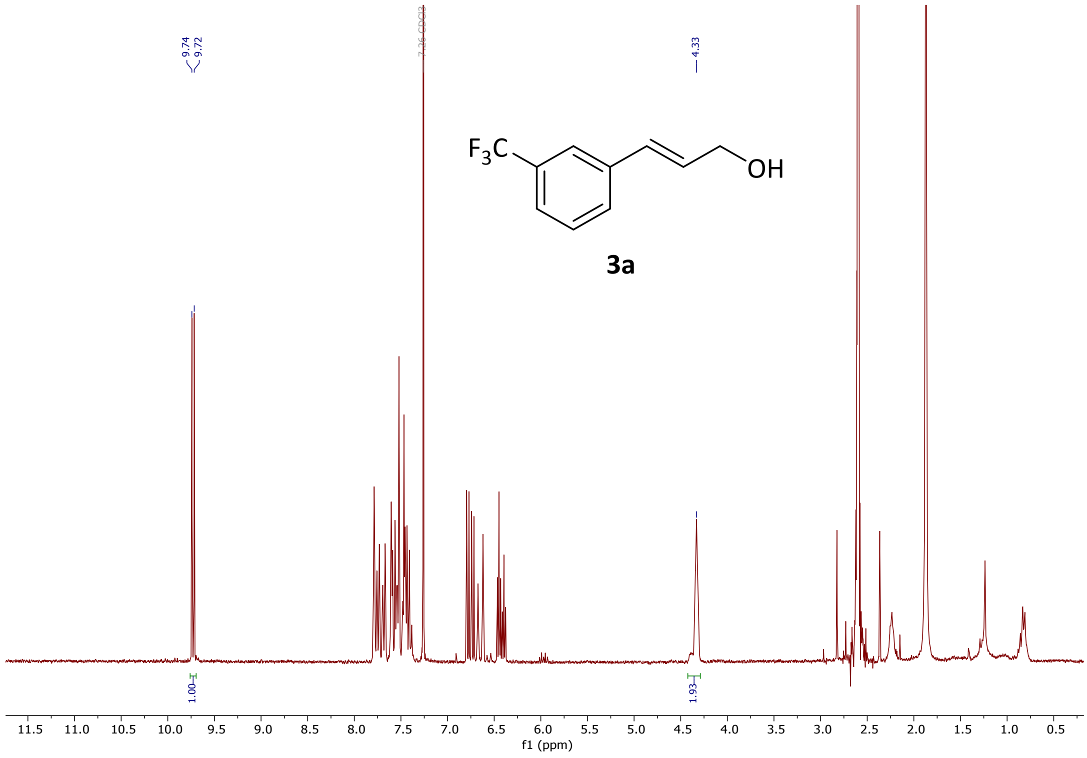

Figure S5. ^{1}H NMR spectrum of the reaction crude in the *Sh*AAO-catalyzed aerobic oxidation of **3a**. (300 Hz, $CDCl_3$).

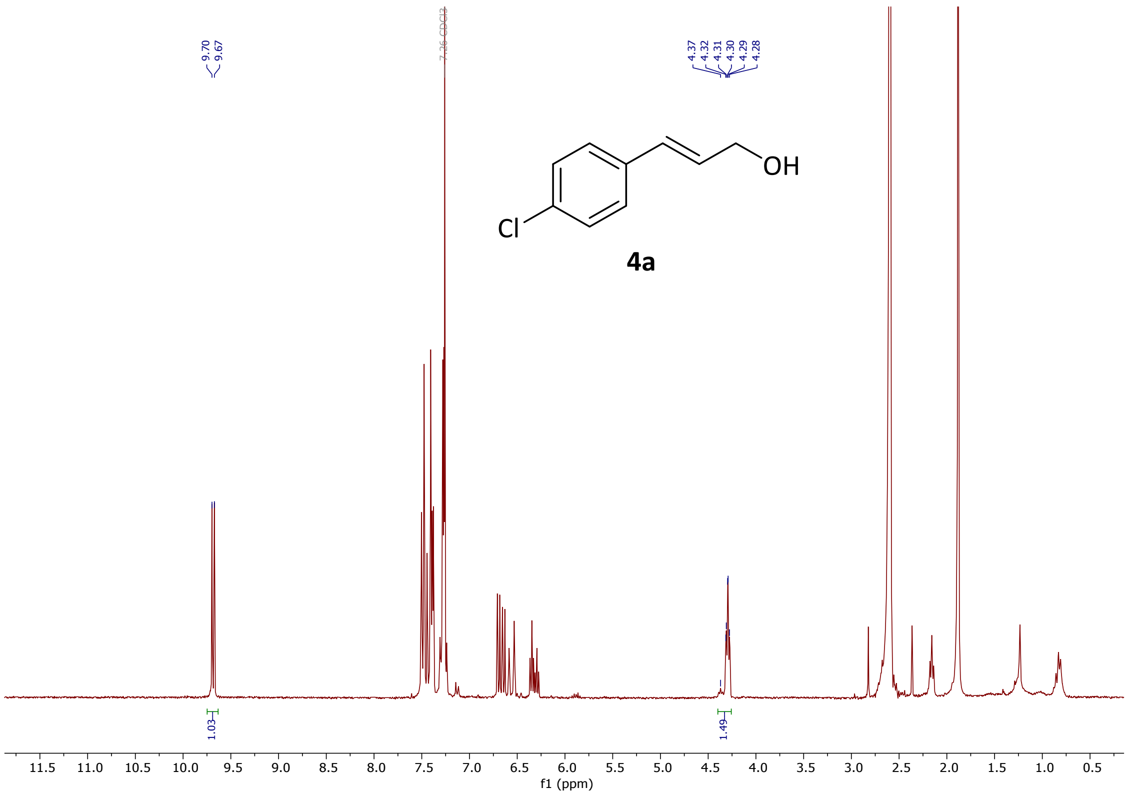

Figure S6. ^{1}H NMR spectrum of the reaction crude in the *Sh*AAO-catalyzed aerobic oxidation of **4a**. (300 Hz, $CDCl_3$).

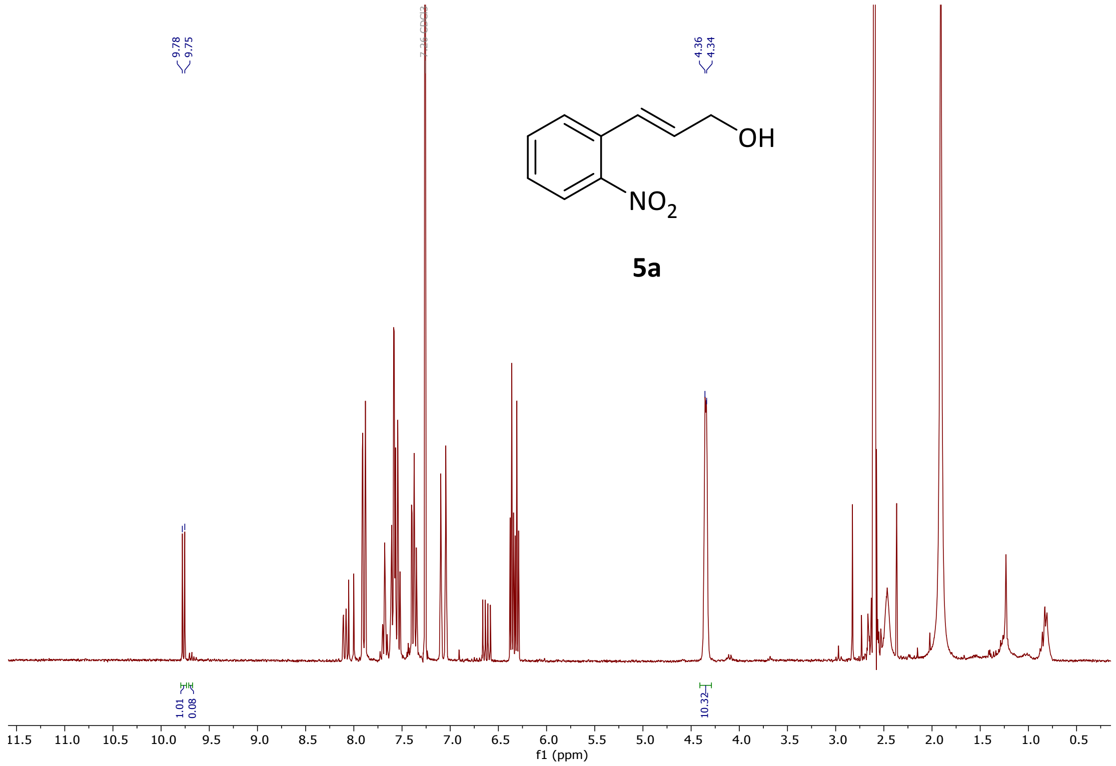

Figure S7. ^{1}H NMR spectrum of the reaction crude in the *Sh*AAO-catalyzed aerobic oxidation of **5a**. (300 Hz, $CDCl_3$).

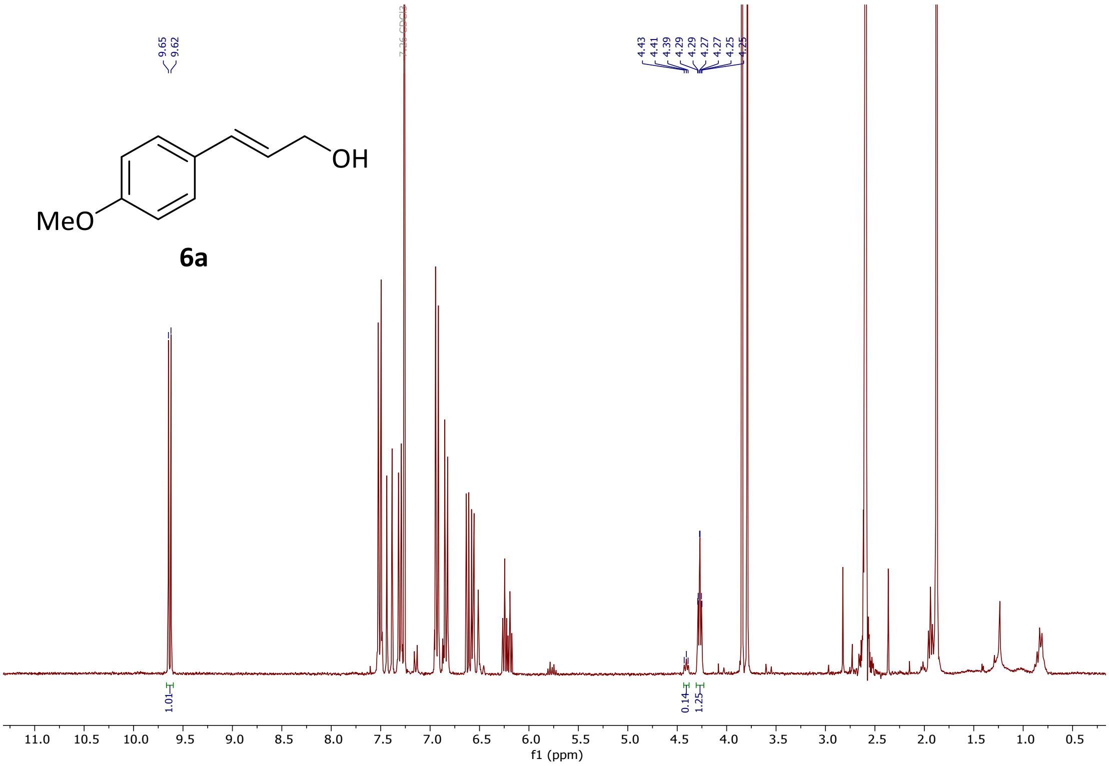

Figure S8. ^{1}H NMR spectrum of the reaction crude in the *Sh*AAO-catalyzed aerobic oxidation of **6a**. (300 Hz, $CDCl_3$).

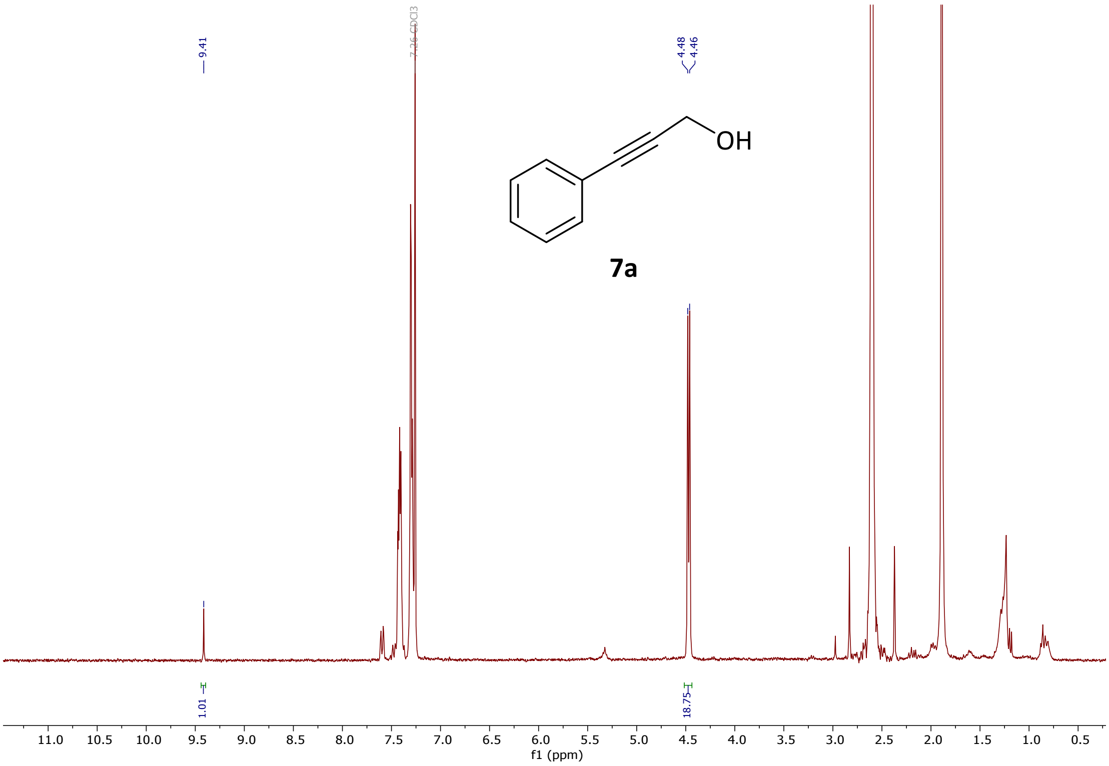

Figure S9. ^{1}H NMR spectrum of the reaction crude in the *Sh*AAO-catalyzed aerobic oxidation of **7a**. (400 Hz, $CDCl_3$).

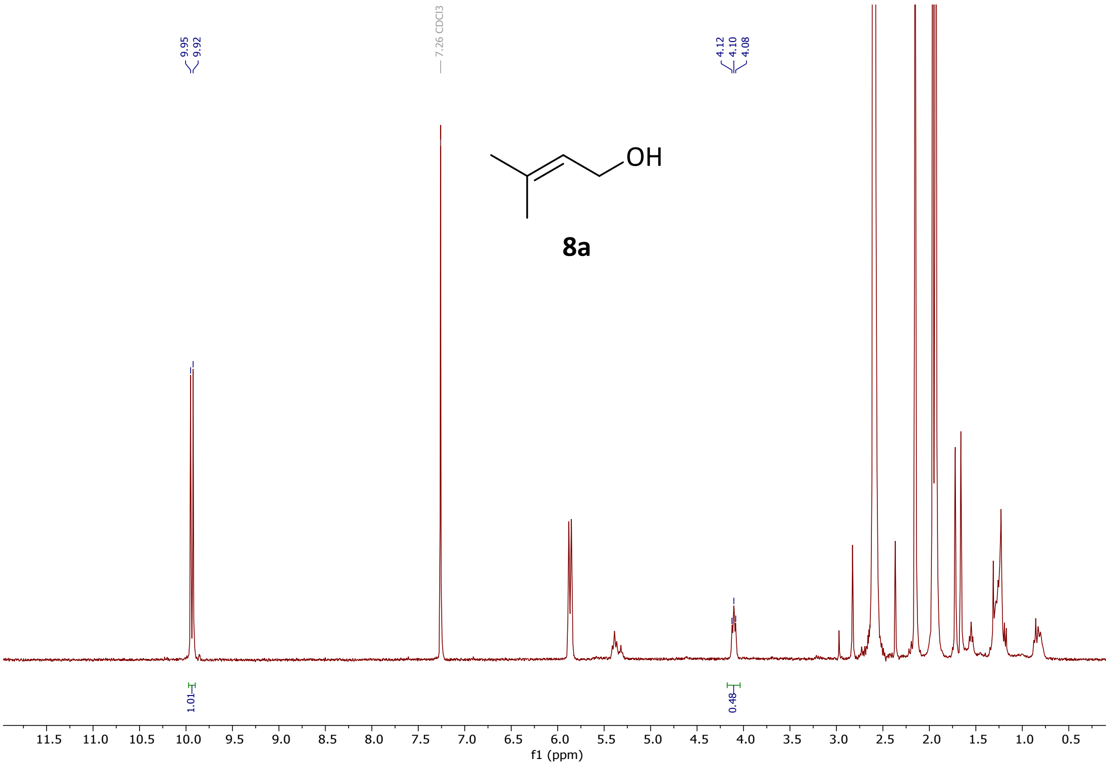

Figure S10. ¹H NMR spectrum of the reaction crude in the *Sh*AAO-catalyzed aerobic oxidation of **8a**. (400 Hz, $CDCl_3$).

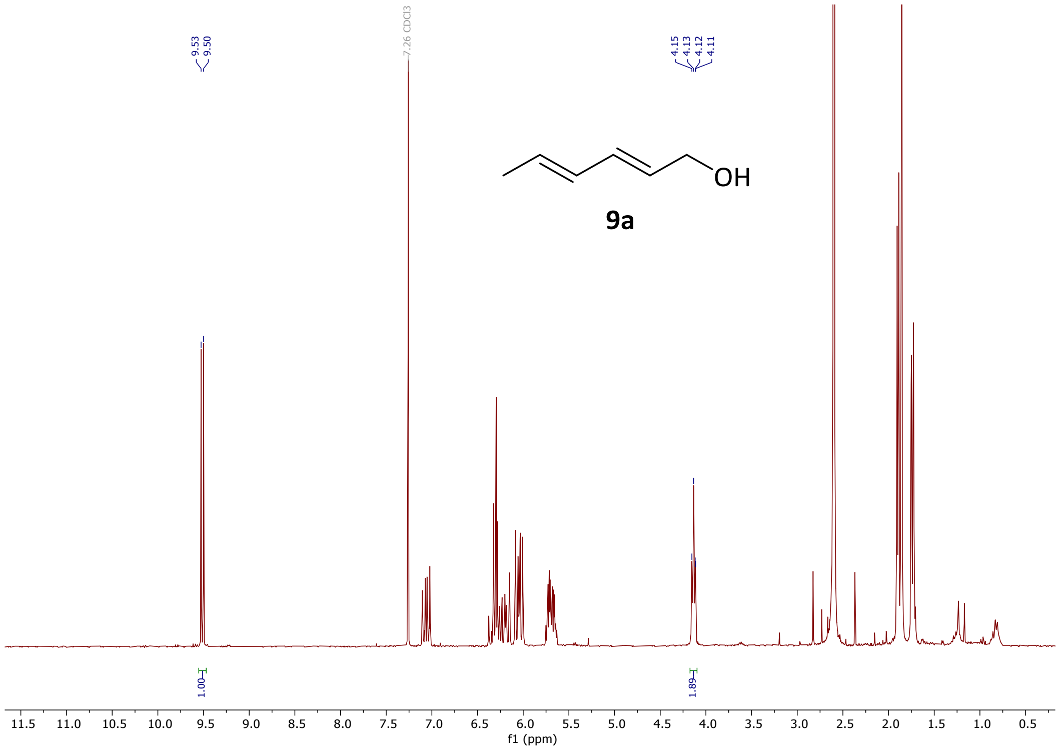

Figure S11. ¹H NMR spectrum of the reaction crude in the *Sh*AAO-catalyzed aerobic oxidation of **9a**. (300 Hz, $CDCl_3$).

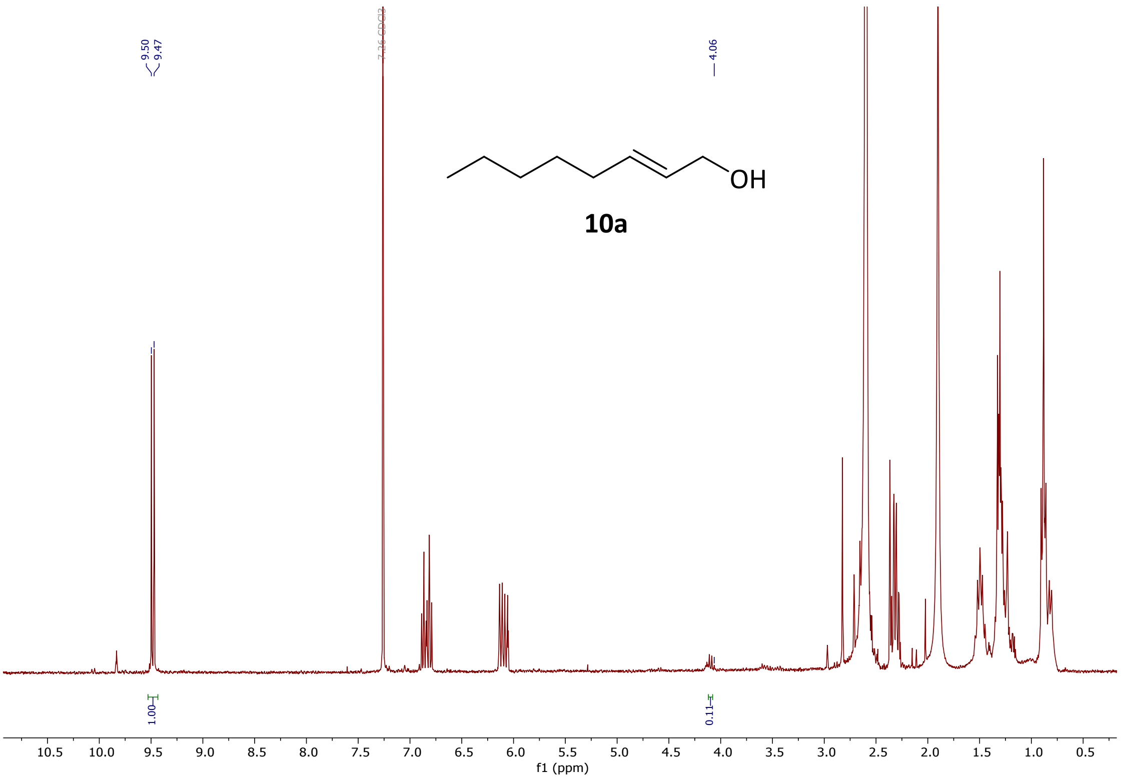

Figure S12. ^{1}H NMR spectrum of the reaction crude in the *Sh*AAO-catalyzed aerobic oxidation of **10a**. (300 Hz, $CDCl_3$).

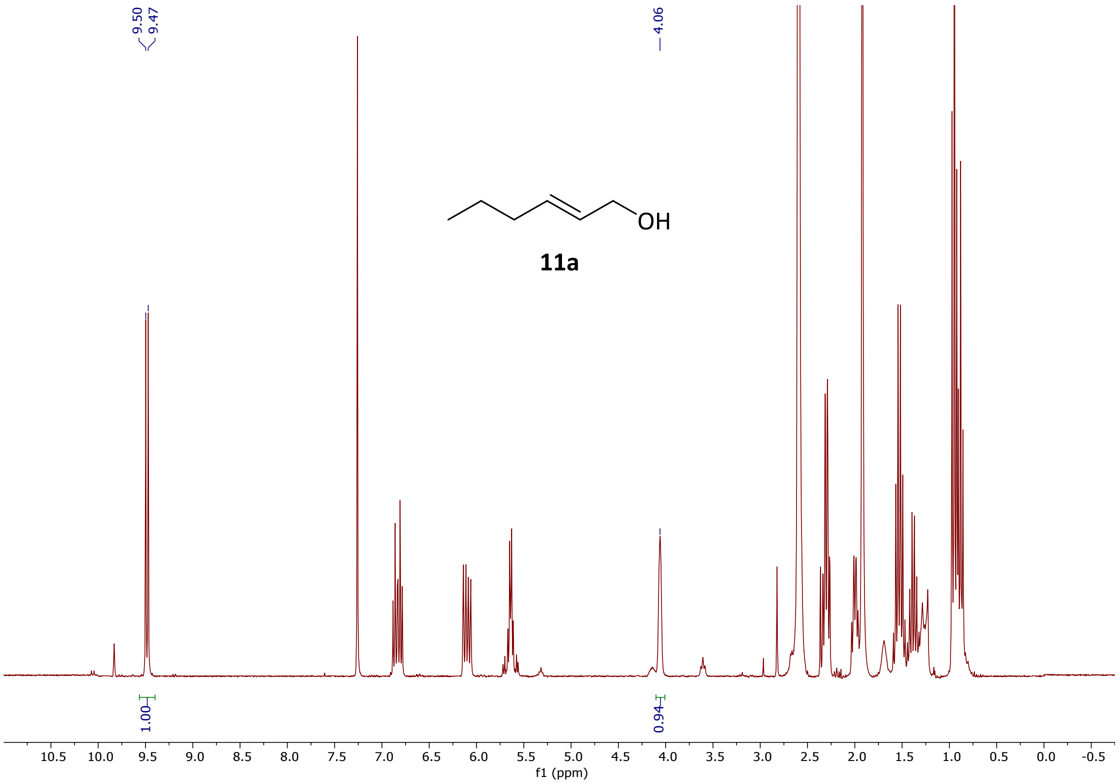

Figure S13. ^{1}H NMR spectrum of the reaction crude in the *Sh*AAO-catalyzed aerobic oxidation of **11a**. (400 Hz, $CDCl_3$).

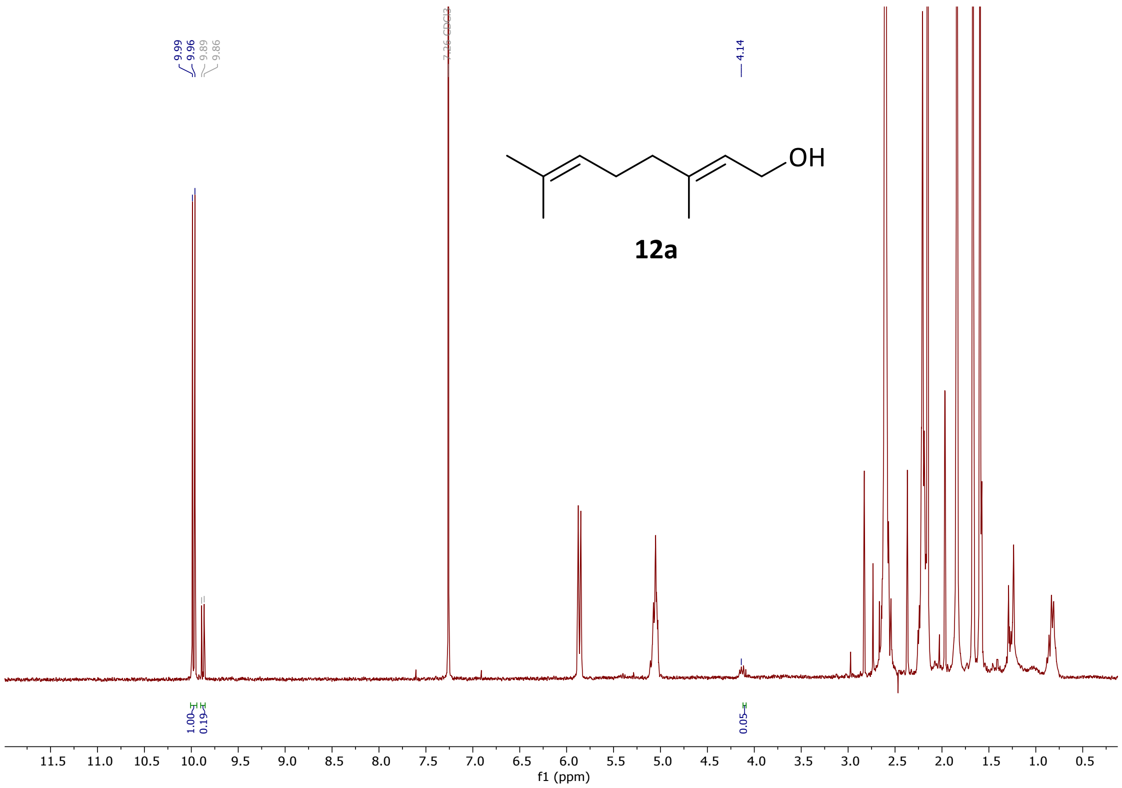

Figure S14. ^{1}H NMR spectrum of the reaction crude in the *Sh*AAO-catalyzed aerobic oxidation of **12a**. (400 Hz, $CDCl_3$).

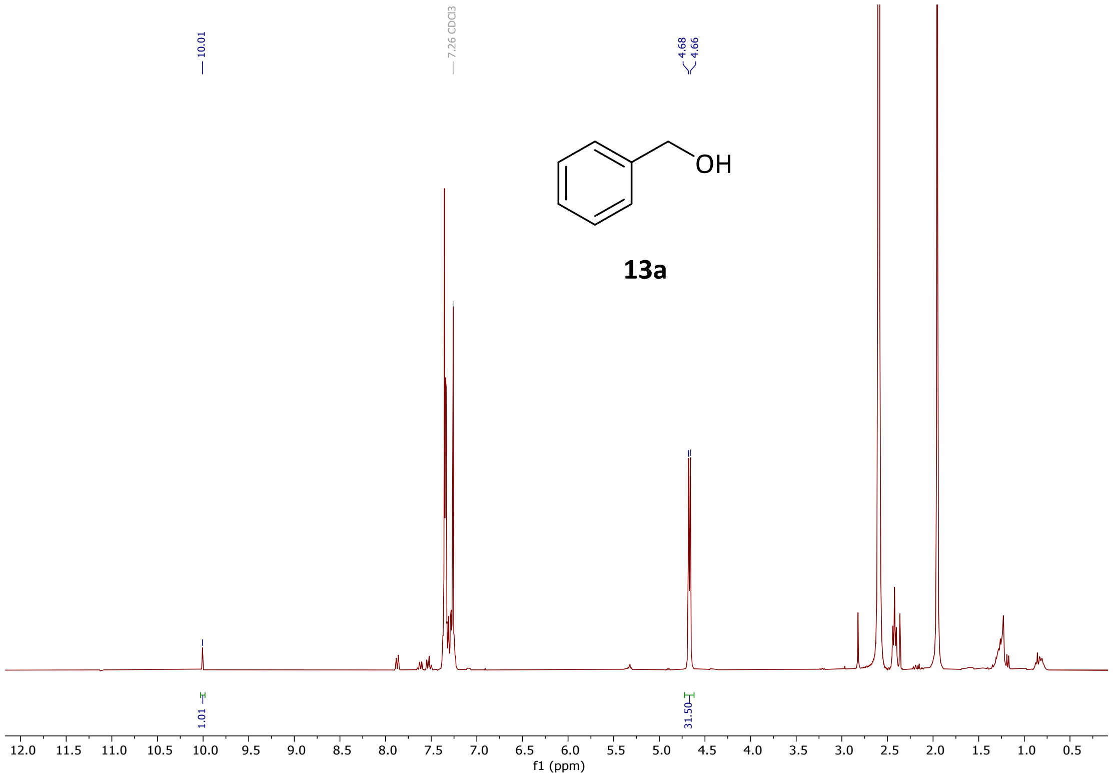

Figure S15. ^{1}H NMR spectrum of the reaction crude in the *Sh*AAO-catalyzed aerobic oxidation of **13a**. (400 Hz, $CDCl_3$).

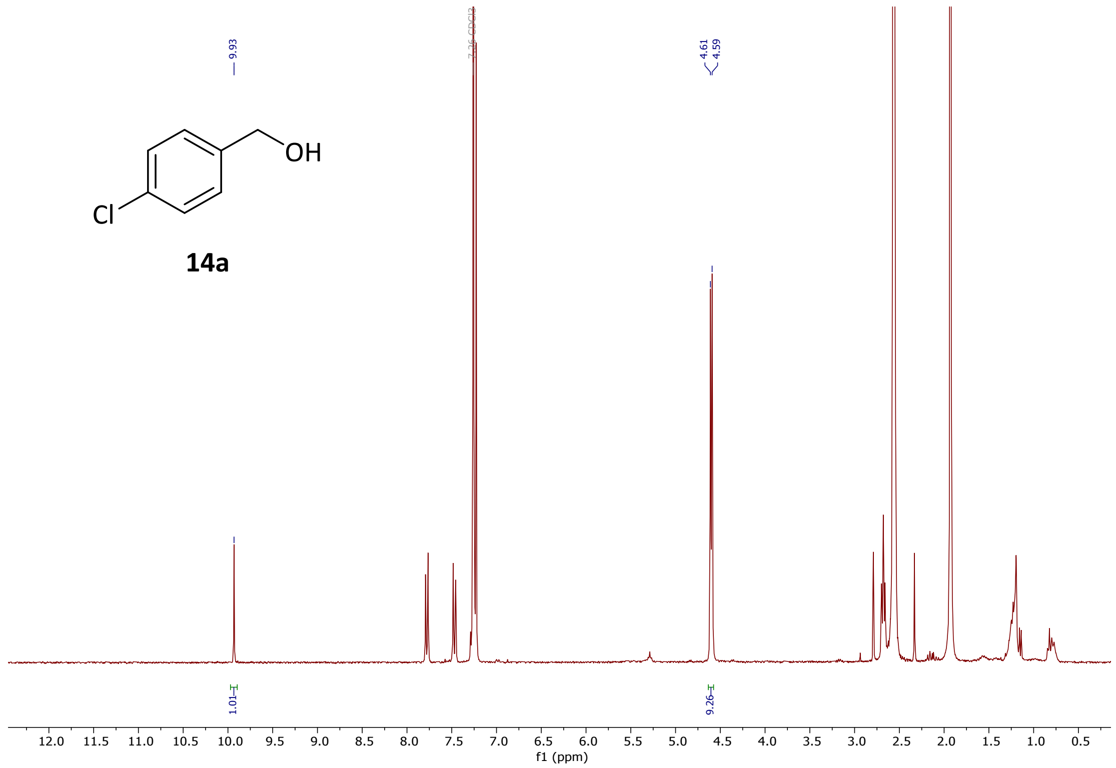

Figure S16. ^{1}H NMR spectrum of the reaction crude in the *Sh*AAO-catalyzed aerobic oxidation of **14a**. (400 Hz, $CDCl_3$).

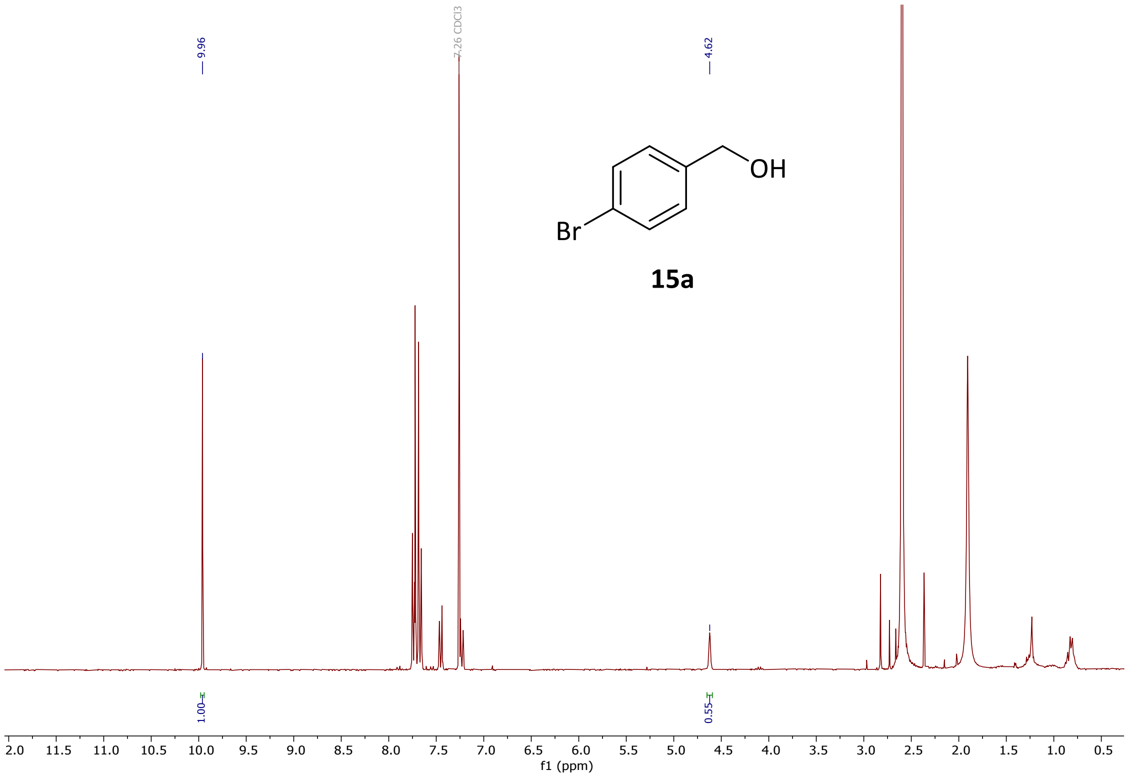

Figure S17. ^{1}H NMR spectrum of the reaction crude in the *Sh*AAO-catalyzed aerobic oxidation of **15a**. (400 Hz, $CDCl_3$).

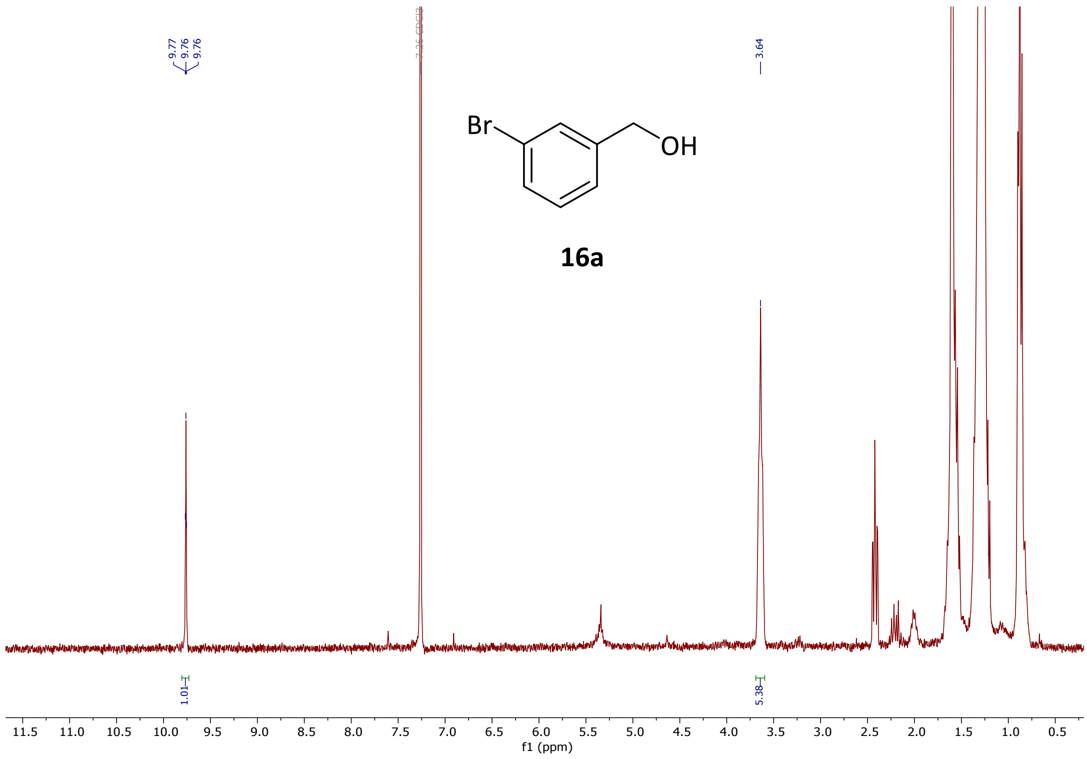

Figure S18. ^{1}H NMR spectrum of the reaction crude in the *Sh*AAO-catalyzed aerobic oxidation of **16a**. (400 Hz, $CDCl_3$).

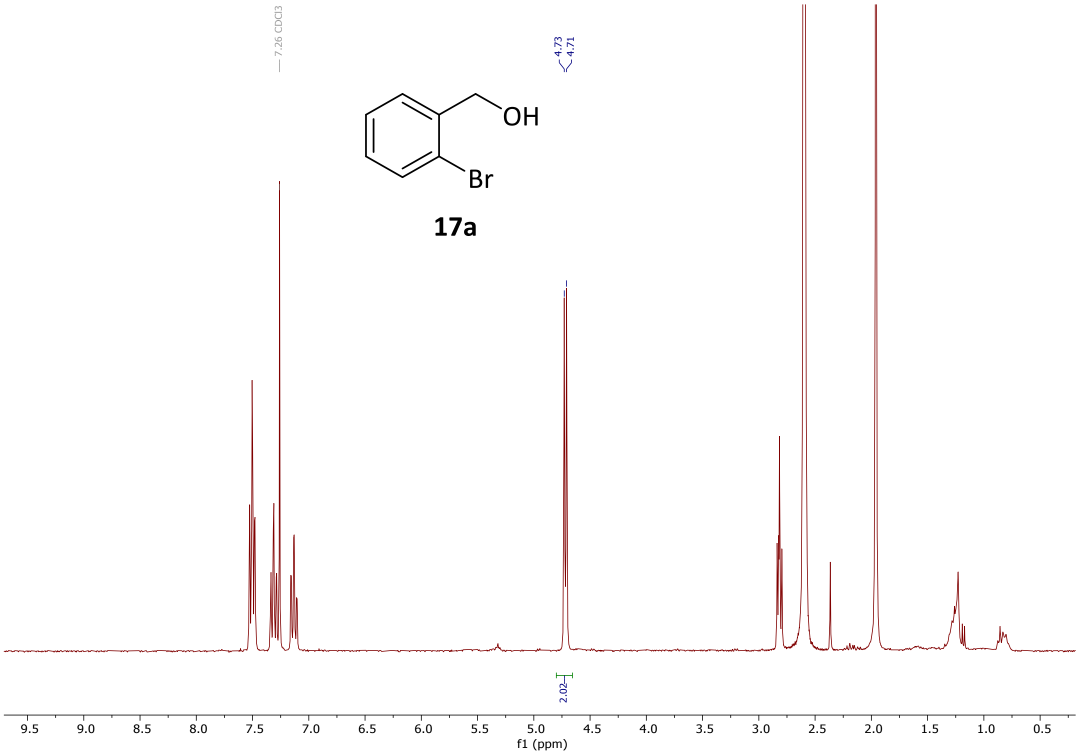

Figure S19. ^{1}H NMR spectrum of the reaction crude in the *Sh*AAO-catalyzed aerobic oxidation of **17a**. (400 Hz, $CDCl_3$).

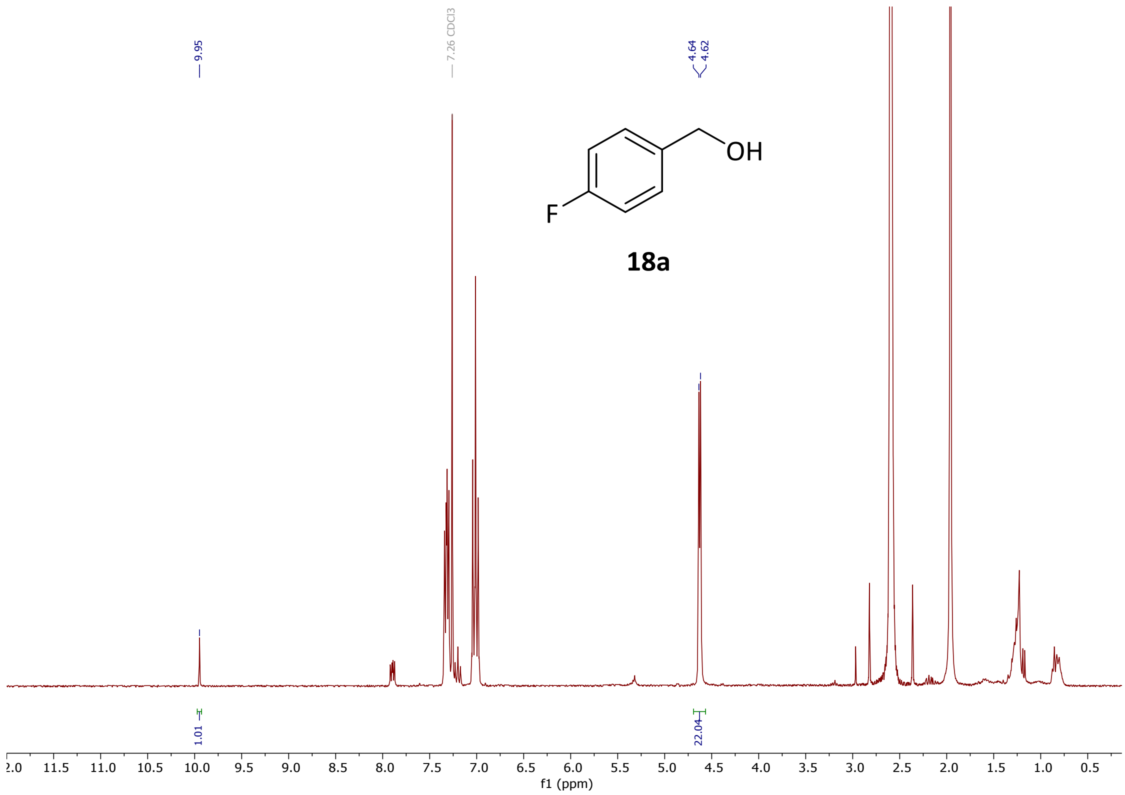

Figure S20. ^{1}H NMR spectrum of the reaction crude in the *Sh*AAO-catalyzed aerobic oxidation of **18a**. (400 Hz, $CDCl_3$).

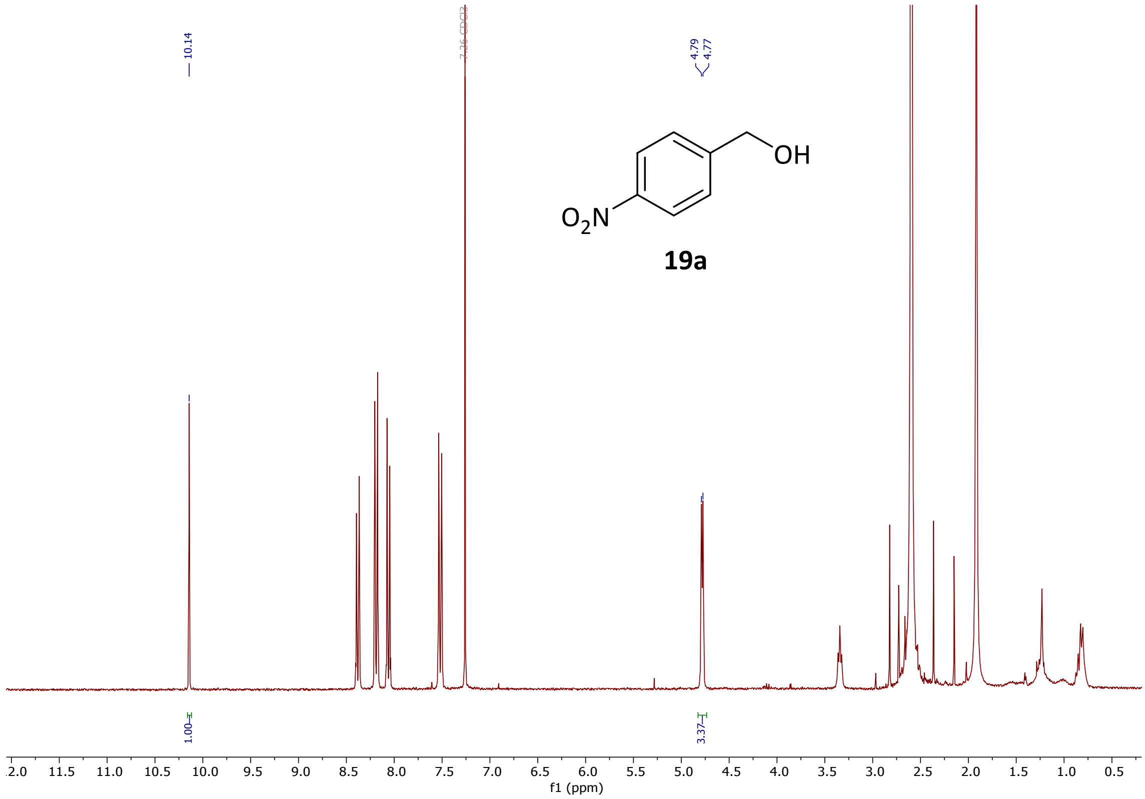

Figure S21. ^{1}H NMR spectrum of the reaction crude in the *Sh*AAO-catalyzed aerobic oxidation of **19a**. (400 Hz, $CDCl_3$).

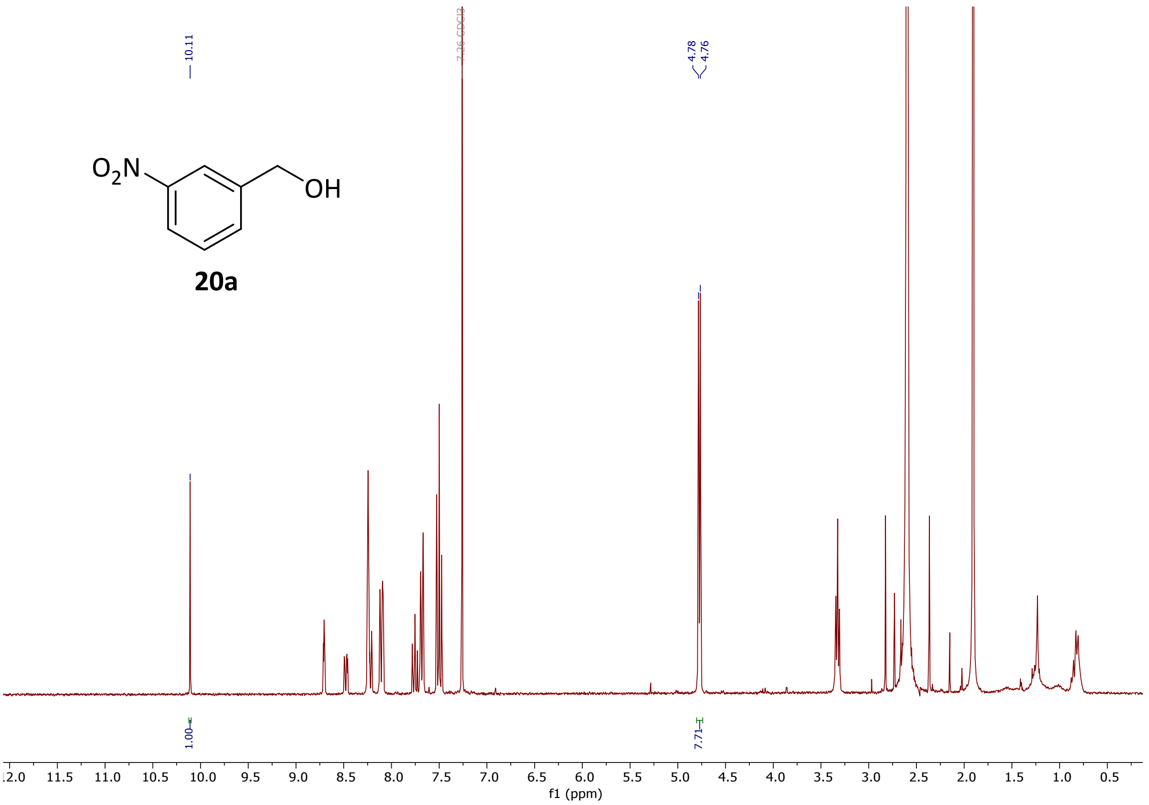

Figure S22. ^{1}H NMR spectrum of the reaction crude in the *Sh*AAO-catalyzed aerobic oxidation of **20a**. (400 Hz, $CDCl_3$).

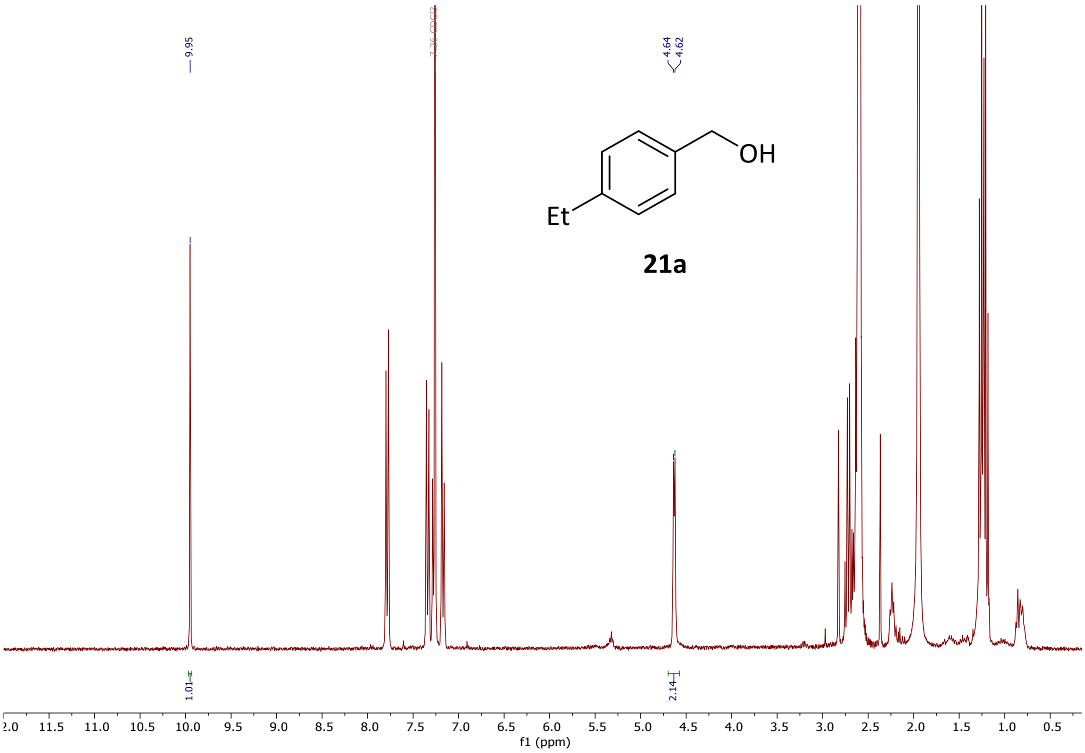

Figure S23. ^{1}H NMR spectrum of the reaction crude in the *Sh*AAO-catalyzed aerobic oxidation of **21a**. (400 Hz, $CDCl_3$).

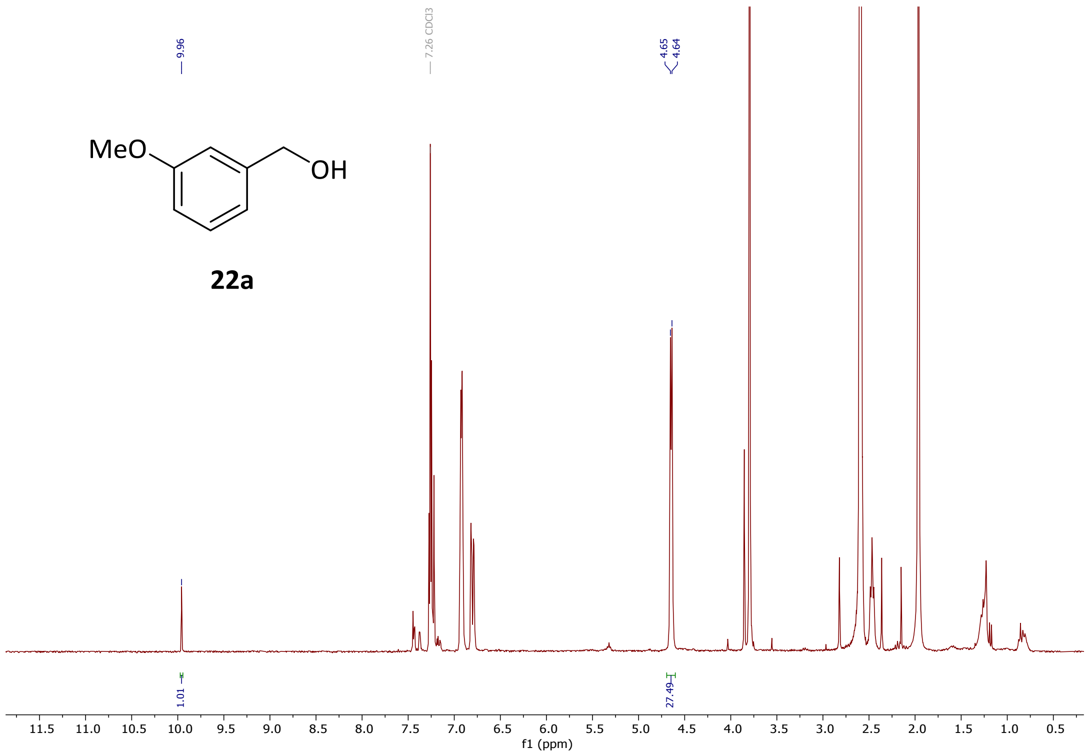

Figure S24. ^{1}H NMR spectrum of the reaction crude in the *Sh*AAO-catalyzed aerobic oxidation of **22a**. (400 Hz, $CDCl_3$).

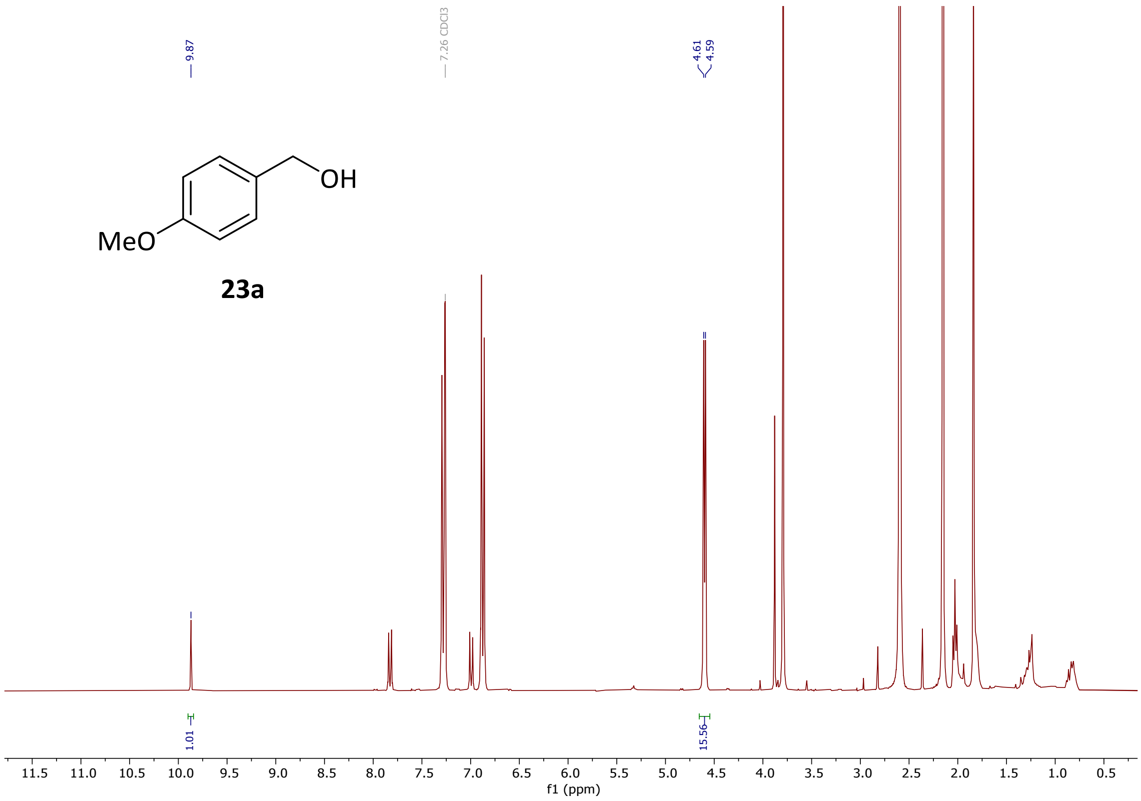

Figure S25. ^{1}H NMR spectrum of the reaction crude in the *Sh*AAO-catalyzed aerobic oxidation of **23a**. (400 Hz, $CDCl_3$).

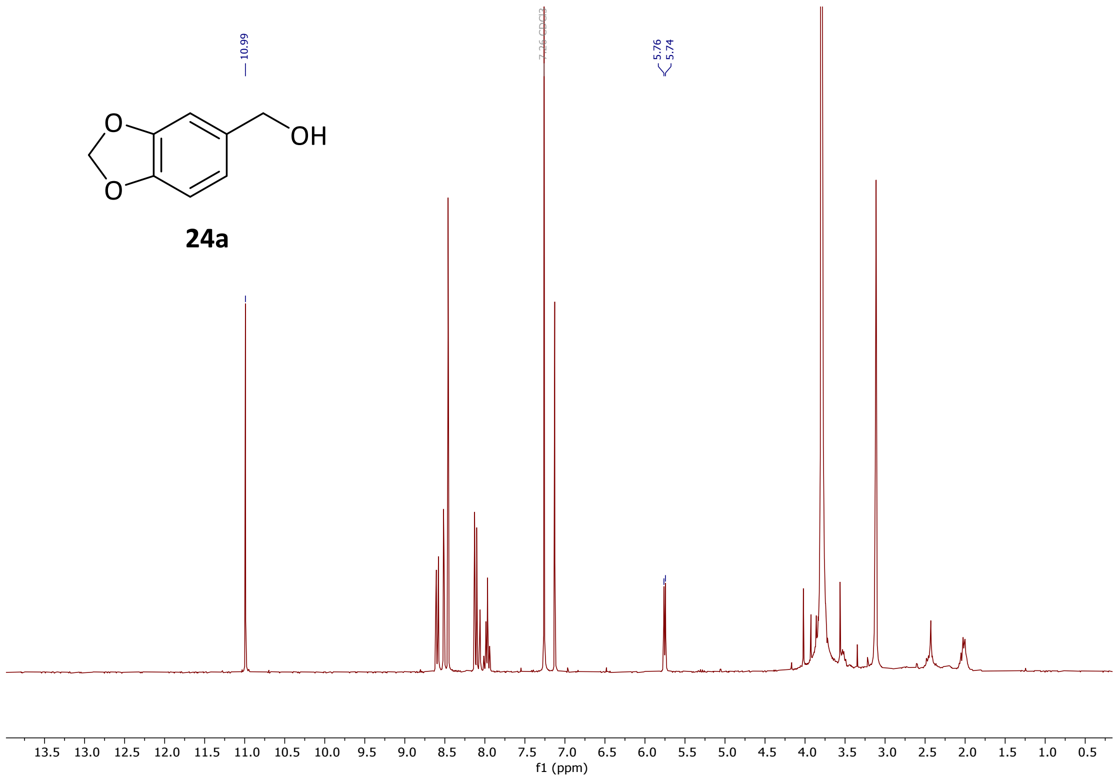

Figure S26. ^{1}H NMR spectrum of the reaction crude in the *Sh*AAO-catalyzed aerobic oxidation of **24a**. (400 Hz, $CDCl_3$).

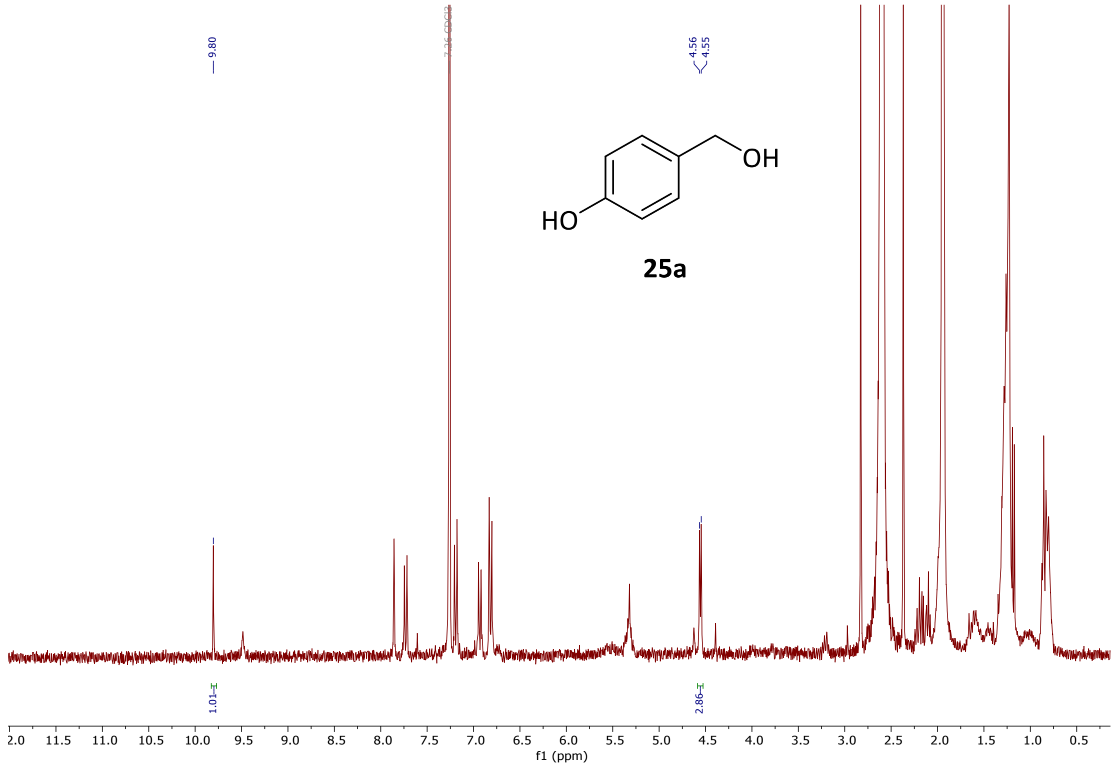

Figure S27. ^{1}H NMR spectrum of the reaction crude in the *Sh*AAO-catalyzed aerobic oxidation of **25a**. (400 Hz, $CDCl_3$).

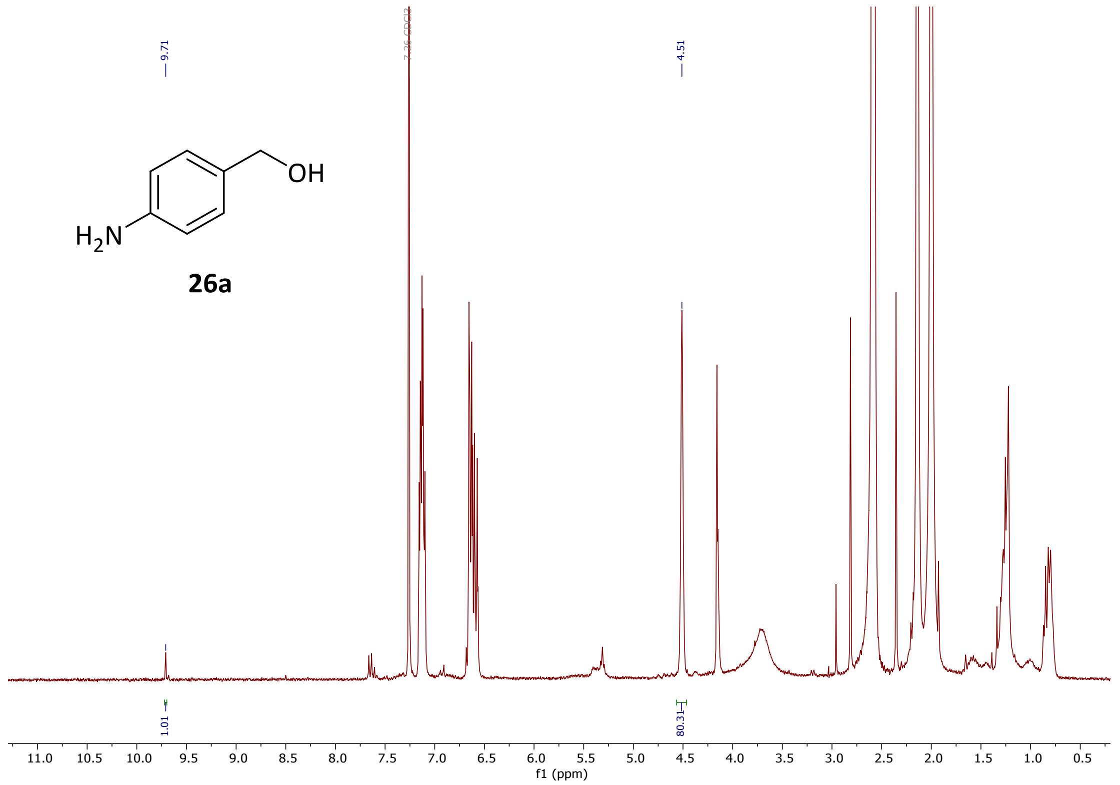

Figure S28. ^{1}H NMR spectrum of the reaction crude in the *Sh*AAO-catalyzed aerobic oxidation of **26a**. (400 Hz, $CDCl_3$).

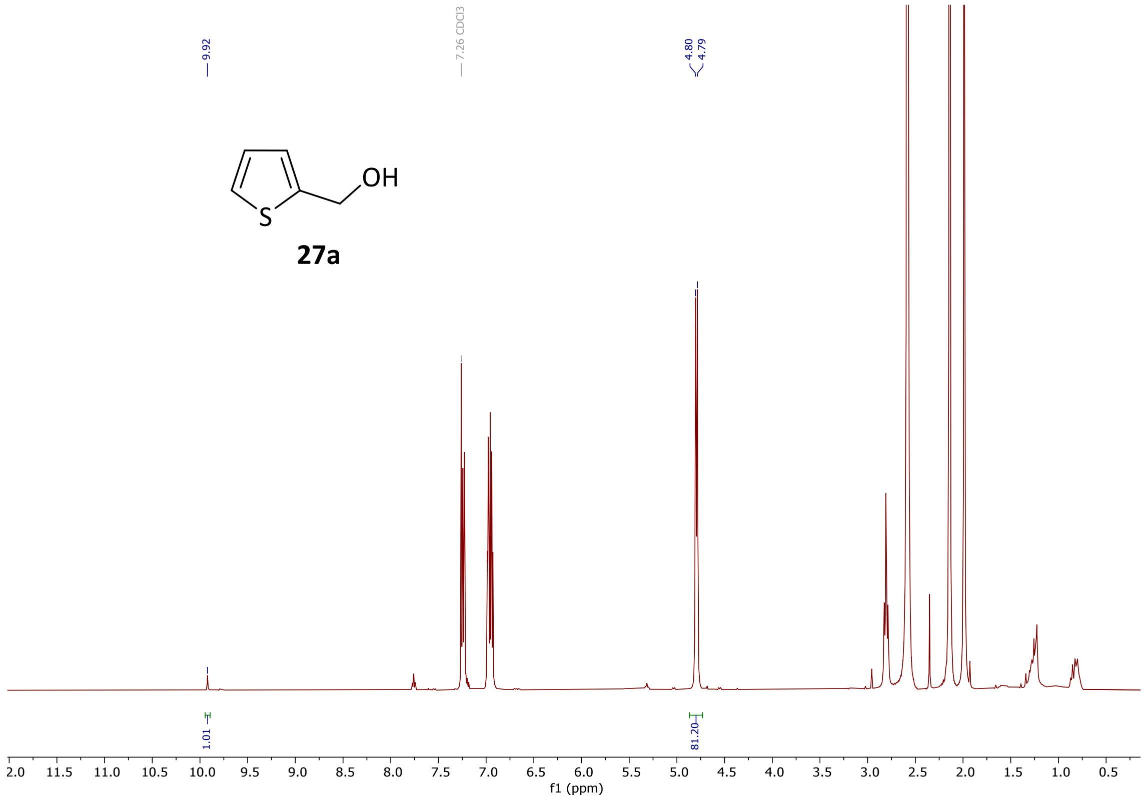

Figure S29. ^{1}H NMR spectrum of the reaction crude in the *Sh*AAO-catalyzed aerobic oxidation of **27a**. (400 Hz, $CDCl_3$).

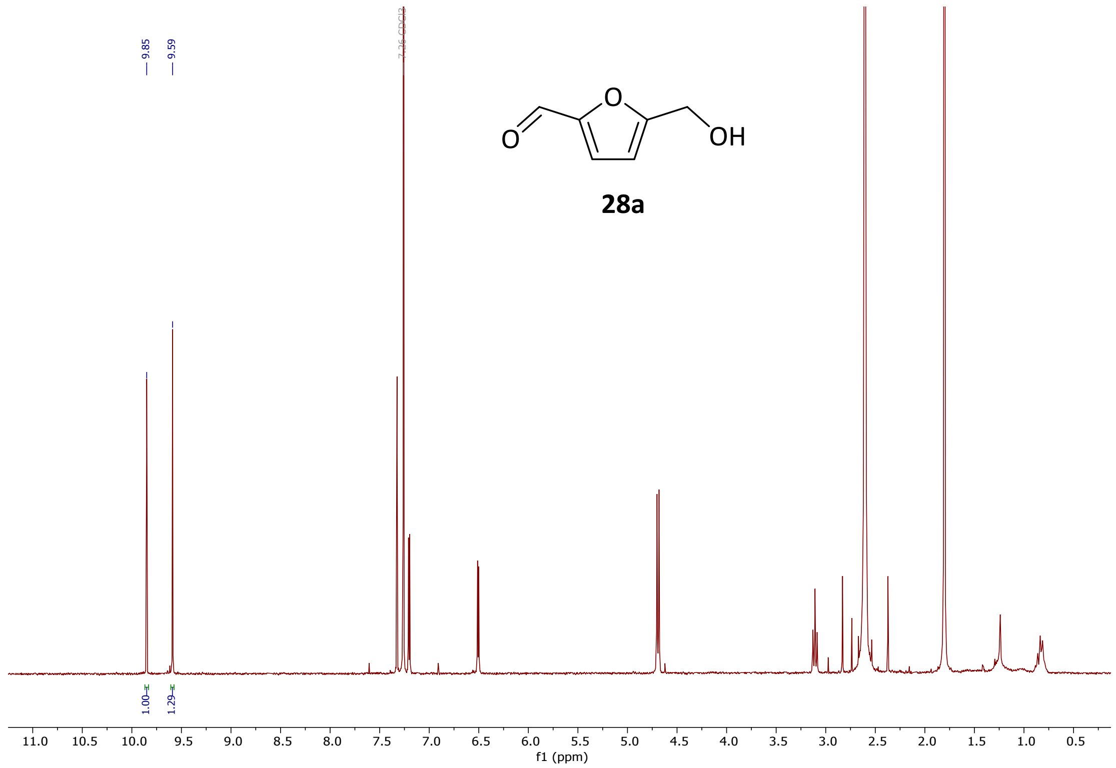

Figure S30. ^{1}H NMR spectrum of the reaction crude in the *Sh*AAO-catalyzed aerobic oxidation of **28a**. (400 Hz, $CDCl_3$).

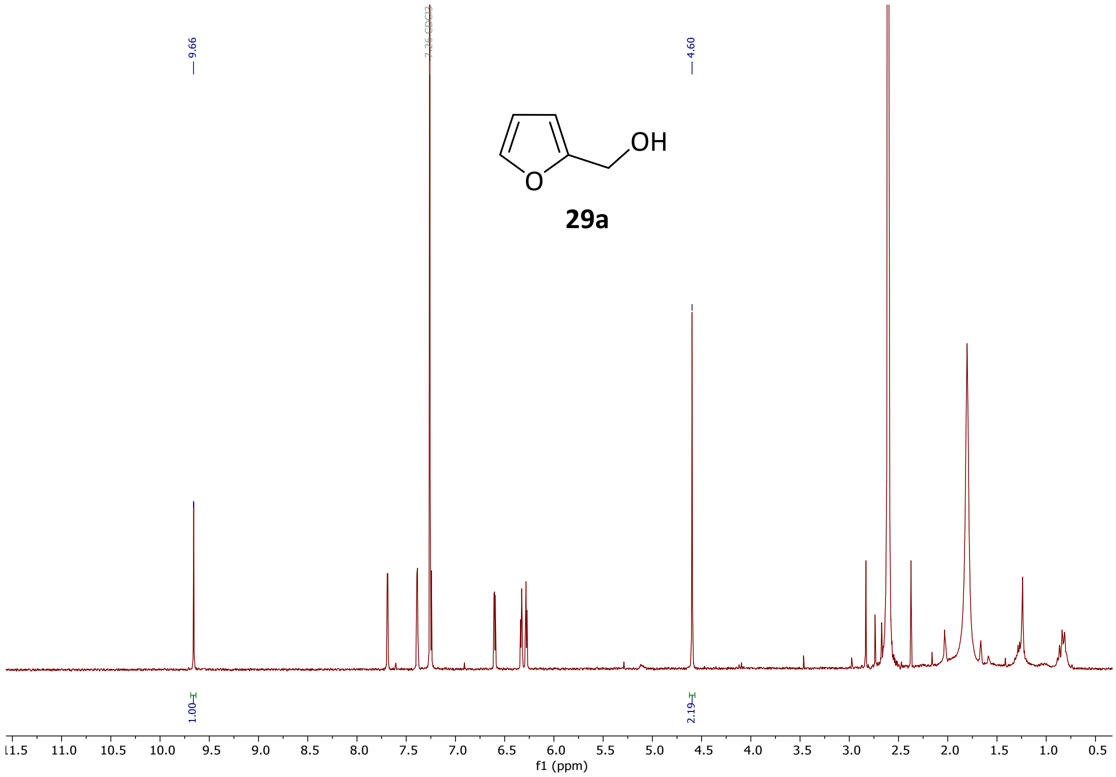

Figure S31. ^{1}H NMR spectrum of the reaction crude in the *Sh*AAO-catalyzed aerobic oxidation of **29a**. (400 Hz, $CDCl_3$).

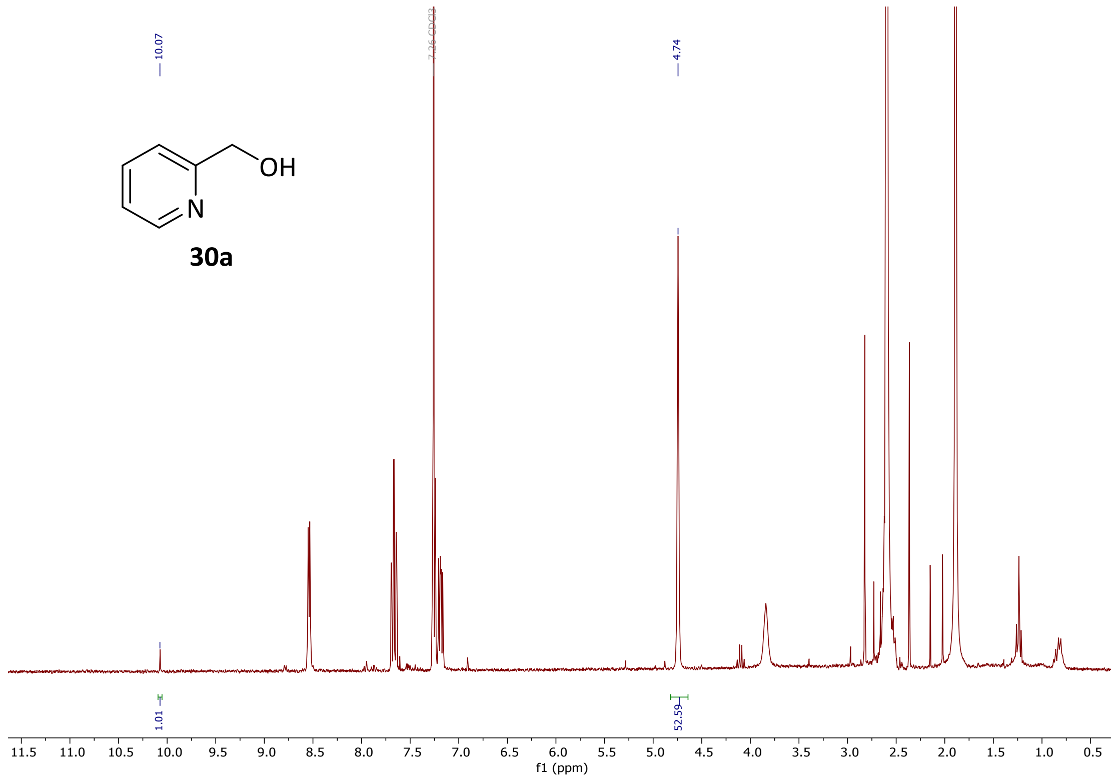

Figure S32. ^{1}H NMR spectrum of the reaction crude in the *Sh*AAO-catalyzed aerobic oxidation of **30a**. (400 Hz, $CDCl_3$).

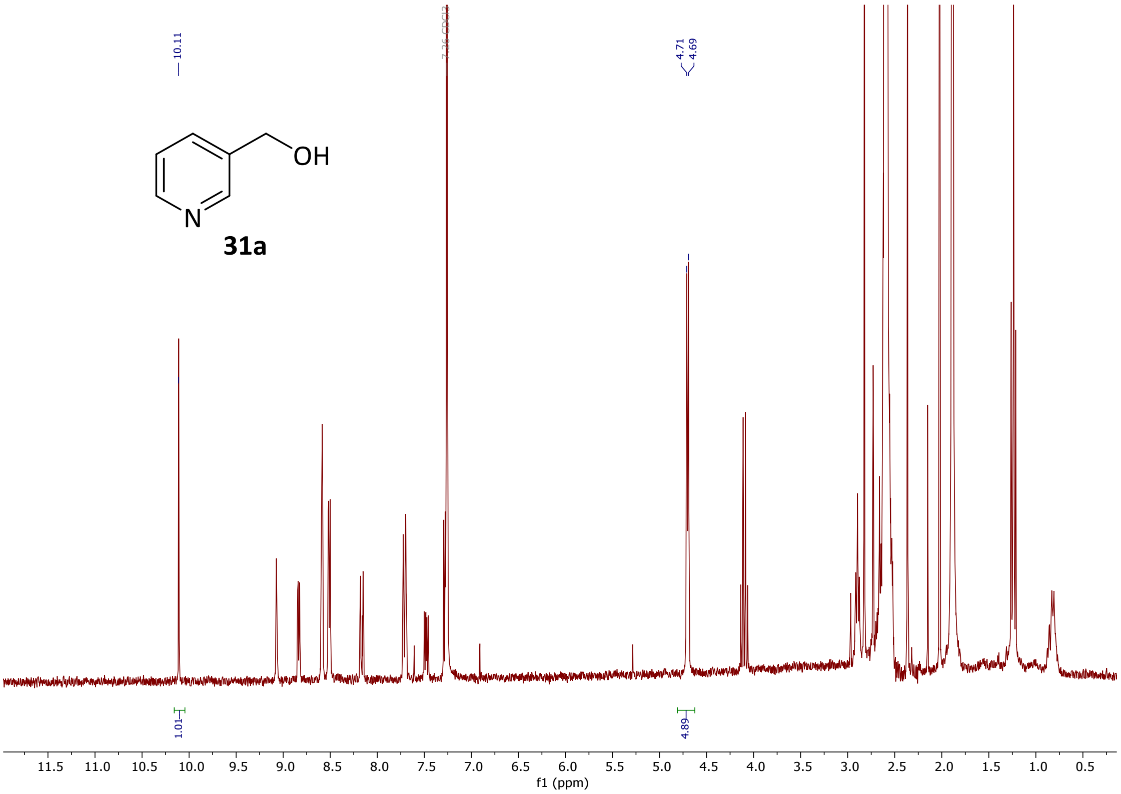

Figure S33. ^{1}H NMR spectrum of the reaction crude in the *Sh*AAO-catalyzed aerobic oxidation of **31a**. (400 Hz, $CDCl_3$).

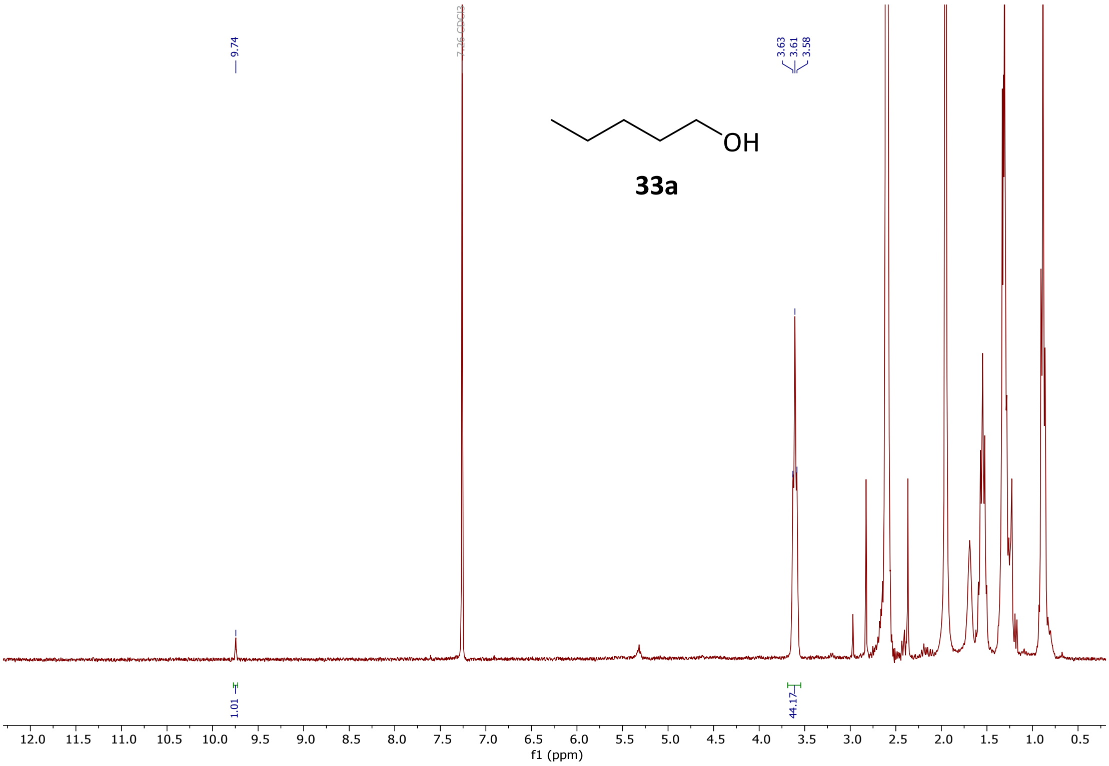

Figure S34. ^{1}H NMR spectrum of the reaction crude in the *Sh*AAO-catalyzed aerobic oxidation of **33a**. (400 Hz, $CDCl_3$).

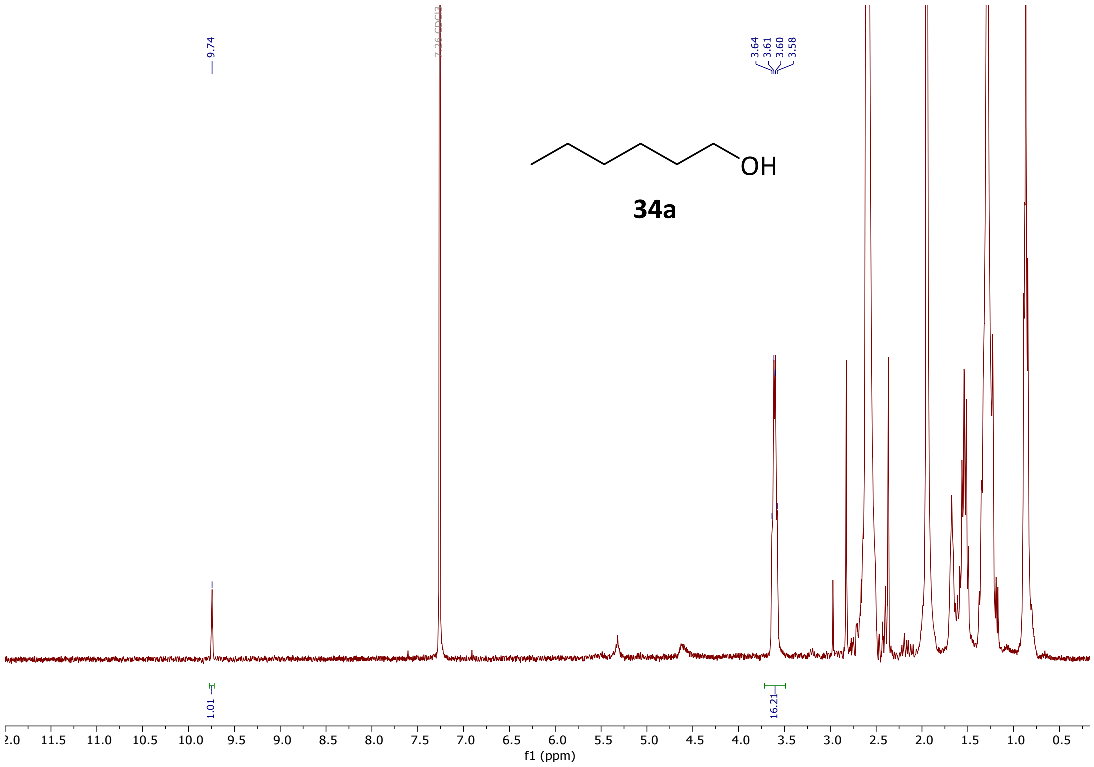

Figure S35. ^{1}H NMR spectrum of the reaction crude in the *Sh*AAO-catalyzed aerobic oxidation of **34a**. (400 Hz, $CDCl_3$).

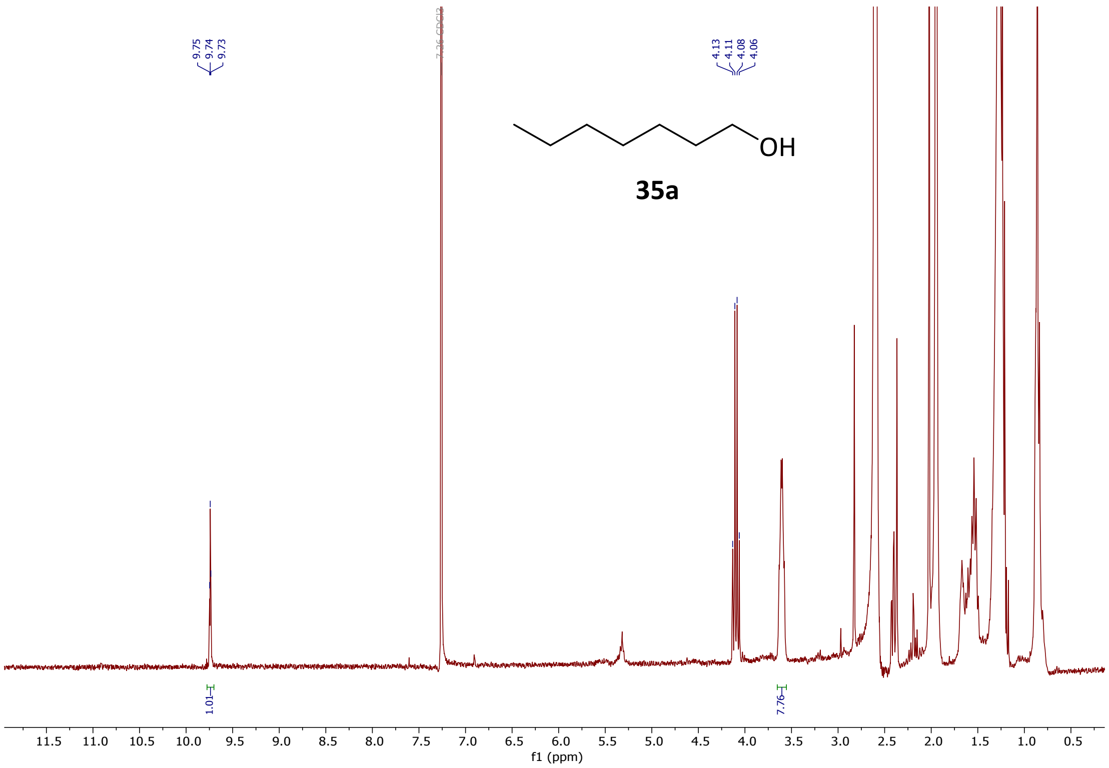

Figure S36. ^{1}H NMR spectrum of the reaction crude in the *Sh*AAO-catalyzed aerobic oxidation of **35a.** (400 Hz, $CDCl_3$).

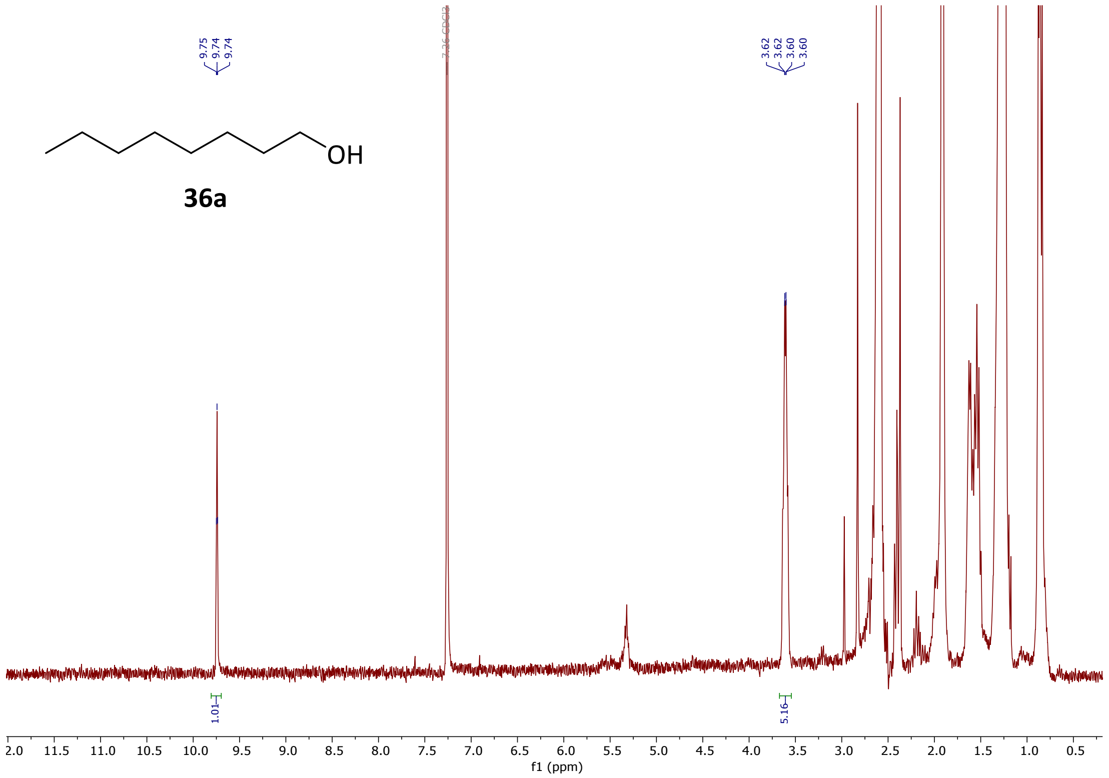

Figure S37. ^{1}H NMR spectrum of the reaction crude in the *Sh*AAO-catalyzed aerobic oxidation of **36a.** (400 Hz, $CDCl_3$).

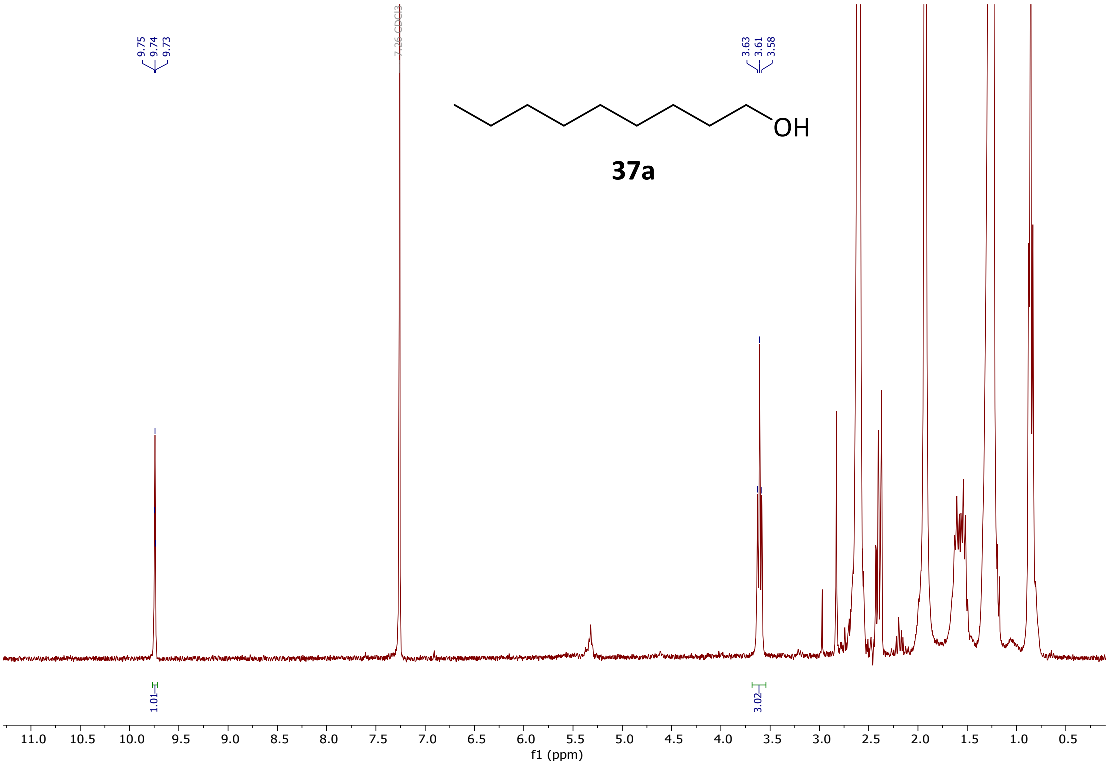

Figure S38. ^{1}H NMR spectrum of the reaction crude in the *Sh*AAO-catalyzed aerobic oxidation of **37a.** (400 Hz, $CDCl_3$).

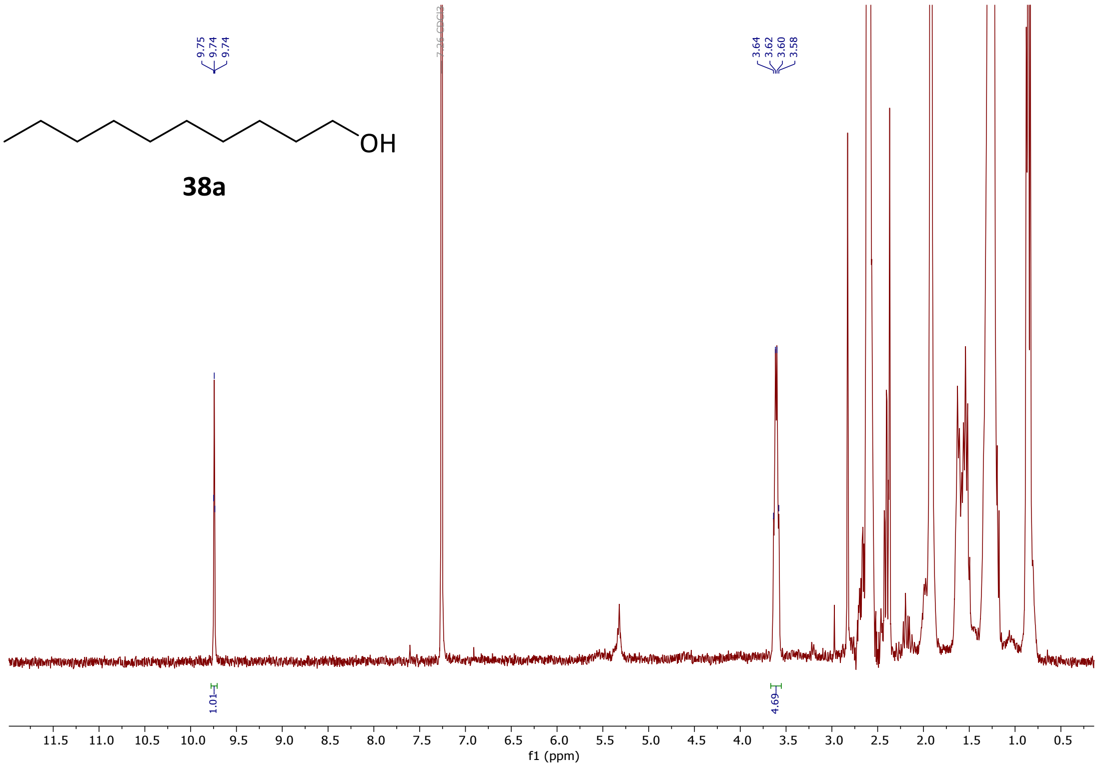

Figure S39. ^{1}H NMR spectrum of the reaction crude in the *Sh*AAO-catalyzed aerobic oxidation of **38a.** (400 Hz, $CDCl_3$).

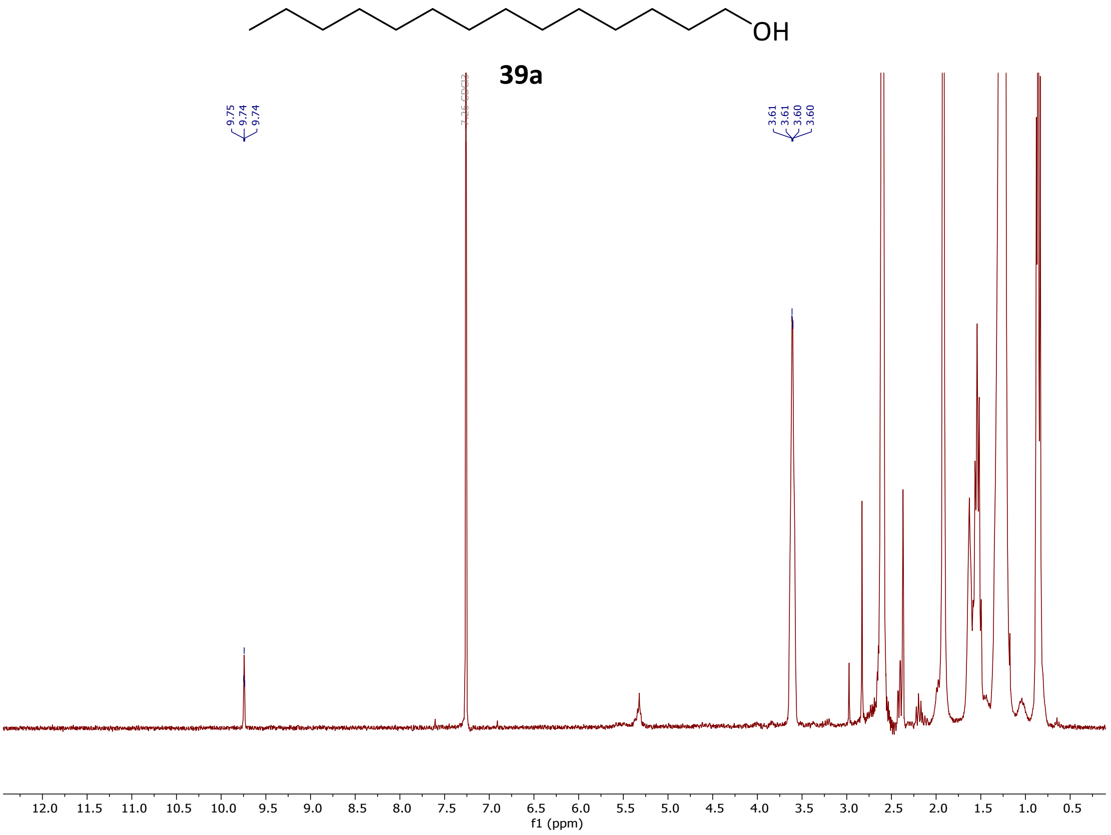

Figure S40. ^{1}H NMR spectrum of the reaction crude in the *Sh*AAO-catalyzed aerobic oxidation of **39a.** (400 Hz, $CDCl_3$).

S9. Synthesis of catalyst III and Singh's Catalyst (IV)

(2*S*,4*R*)-4-((*tert*-butyldiphenylsilyl)oxy)pyrrolidine-2-carboxylic acid (III)

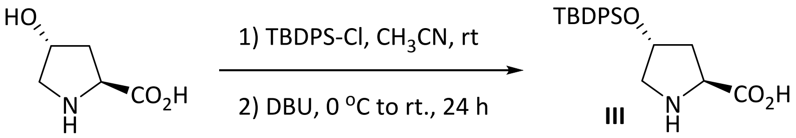

To a solution containing *trans*-4-hydroxy-L-proline (1.0 g, 7.63 mmol) in acetonitrile (20 mL), TBDPS-Cl (6.94 mL, 26 mmol) was added and the mixture stirred until a homogeneous solution was obtained. The reaction was then cooled to 0 °C and DBU (4.22 mL, 28 mmol) added. The resulting mixture was stirred for 24 hours at r.t.. The resulting reaction mixture was extracted with hexane (3 × 30 mL), organic phases combined, dried over $MgSO_4$ and the solvent removed under reduced pressure. The resulting oil was redissolved in a mixture of methanol (32 mL), THF (18 mL), water (16 mL), and 2 M NaOH (24 mL), and stirred for 90 minutes at room temperature. The pH was carefully adjusted to 6 with 2 M HCl before removing the organic solvents under reduced pressure. A 1:1 ratio of diethyl ether (Et_2O) was added to the resulting aqueous phase, and the biphasic mixture left to stand for 24 hours, leading to the formation of crystals in the organic phase. The solid was finally filtered and washed with cold Et_2O to obtain the silylated intermediate **III** as white crystals with a yield of 86% (2.42 g).

^{1}H NMR (300 MHz, MeOD) δ 8.01 – 7.86 (m, 4H), 7.85 – 7.56 (m, 6H), 4.94 – 4.81 (m, 2H), 3.70 – 3.57 (m, 2H), 2.67 – 2.48 (m, 1H), 2.43 – 2.23 (m, 1H), 1.35 (s, 9H).

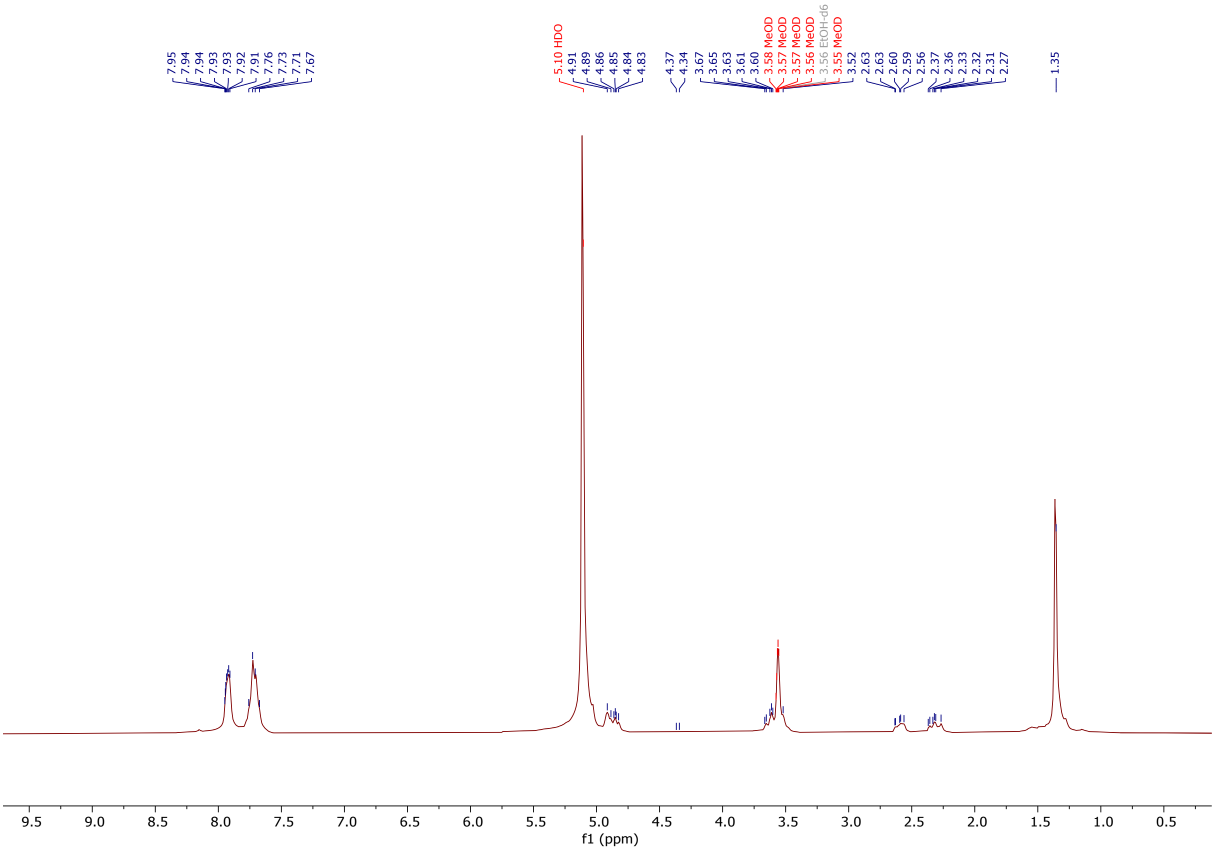

Figure S41. ^{1}H NMR spectrum of organocatalyst **III**. (300 Hz, $CDCl_3$).

(*S*)-N-((*S*)-1-hydroxy-4-methyl-1,1-diphenilpentan-2-yl)pyrrolidine-2-carboxamide (Singh's Catalyst, IV)

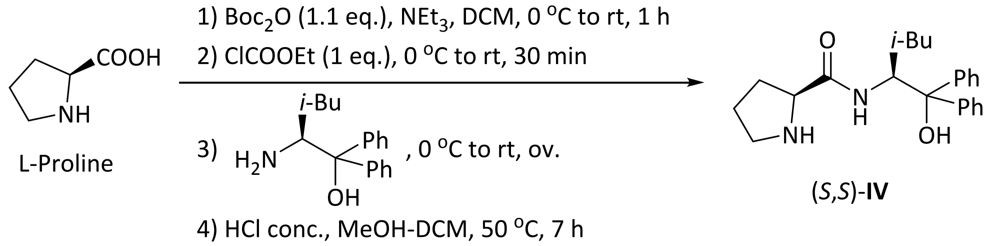

To a suspension of L-proline (1.15 g, 10 mmol) and Boc_2O (2.29 g, 10.5 mmol) in dry DCM (200 mL), NEt_3 (4.5 mL, 32.4 mmol) was slowly added at 0 °C. The ice bath was removed, and the reaction mixture was stirred at room temperature for 1 hour. During this time, the mixture became homogeneous as proline reacted with Boc_2O to form the N-Boc-proline intermediate. The mixture was then cooled again to 0 °C and treated with ethyl chloroformate (0.95 mL, 10 mmol), forming triethylammonium chloride as a white precipitate. After the addition was complete, the resulting mixture was stirred for an additional 30 minutes. Solid (*S*)-2-amino-4-methyl-1,1-diphenylpentan-1-ol (9.9 mmol) was then added portionwise using a powder funnel. After the addition was complete, the reaction mixture was stirred from 0 °C to room temperature overnight. The volatiles were then evaporated under reduced pressure, yielding N-Boc-prolinamide as a pale yellow solid. MeOH (16 mL) and DCM (8 mL) were added, followed by the slow addition of concentrated HCl (4 mL). The resulting mixture was heated to 50 °C using an oil bath and stirred for 7 hours. During this time, the mixture became homogeneous (after approximately 2.5 hours). Once the reaction was complete (as confirmed by TLC analysis), the resulting

solution was evaporated under reduced pressure and diluted with water (20 mL) and EtOAc (30 mL). The mixture was cooled to 0 °C, and the pH was adjusted to 8–9 using 6 N NaOH (~7 mL) under vigorous stirring. After separating the organic phase, the aqueous phase was extracted with EtOAc (3 × 20 mL). The combined organic layers were washed with 1 N NaOH, water, and brine, then dried over $MgSO_4$. The resulting solution was filtered through a short silica pad and concentrated under reduced pressure. Finally, the product was purified by recrystallization from EtOAc, affording (*S*,*S*)-**IV** as a white crystalline solid with a 64% yield (2.3 g).

^{1}H NMR (300 MHz, CDCl3) δ 7.94 (d, *J* = 8.4 Hz, 1H), 7.59 – 7.51 (m, 4H), 7.34 – 7.27 (m, 2H), 7.25 – 7.08 (m, 4H), 5.44 (s, 1H), 4.58 (ddd, *J* = 11.0, 8.4, 2.2 Hz, 1H), 3.48 (dd, *J* = 9.6, 4.7 Hz, 1H), 2.82 (dt, *J* = 10.1, 6.7 Hz, 1H), 2.56 (dt, *J* = 10.0, 6.3 Hz, 1H), 1.94 – 1.81 (m, 2H), 1.60 – 1.52 (m, 1H), 1.50 – 1.39 (m, 2H), 1.29 – 1.17 (m, 2H), 0.91 (d, *J* = 6.5 Hz, 3H), 0.86 (d, *J* = 6.7 Hz, 3H).

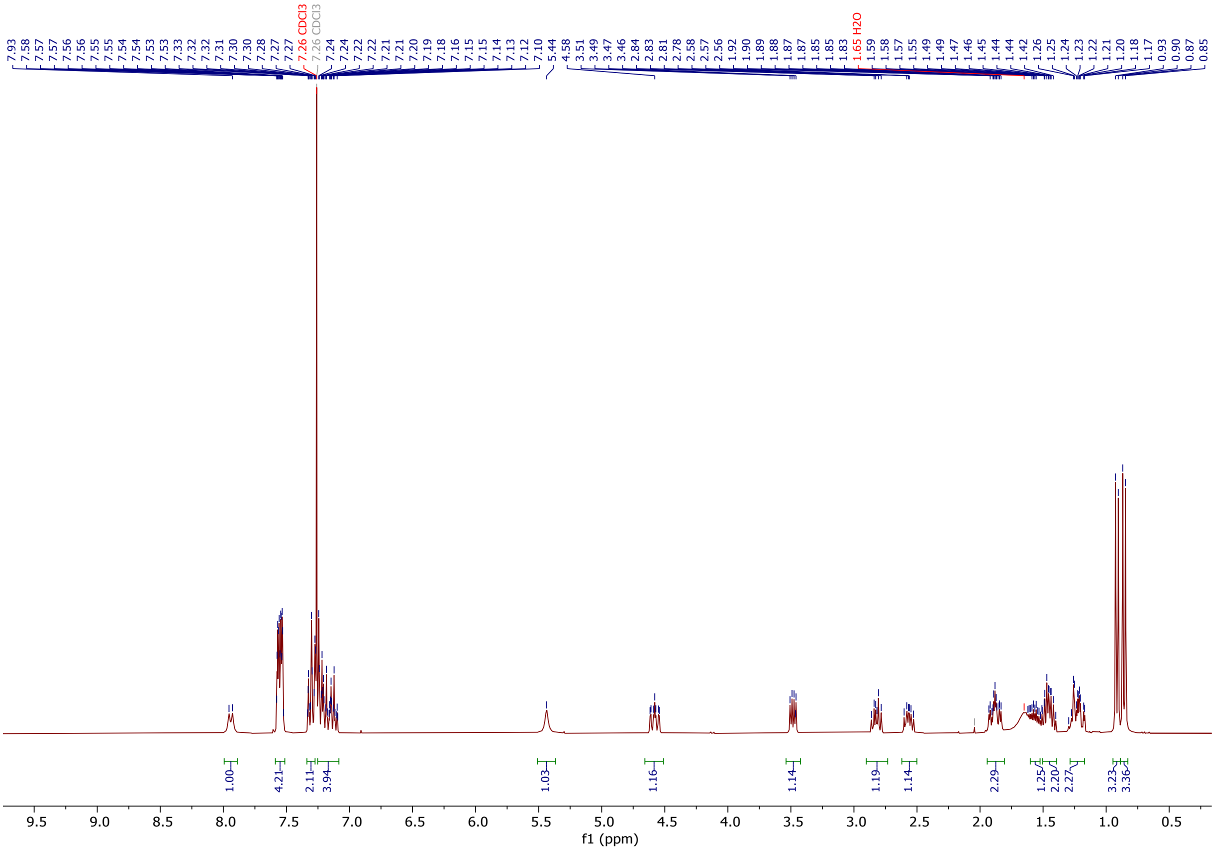

Figure S42. ^{1}H NMR spectrum of organocatalyst (*S*,*S*)-**IV**. (300 Hz, $CDCl_3$).

S10. Initial optimization of the biooxidation step for chemoenzymatic cascades.

Scheme S2. Effect of the reaction vessel in the *Sh*AAO-mediated oxidation of 1a, 9a and 12a.

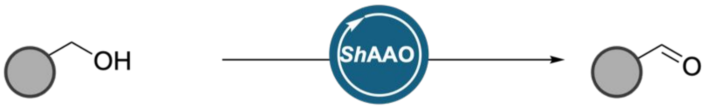

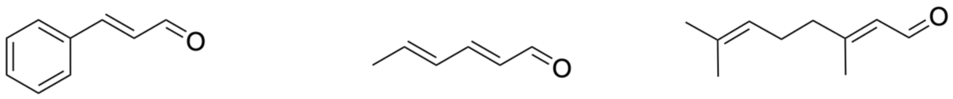

1b
2 mL eppendorf: 54% conv.
10 mL vial: 97% conv.

9b
2 mL eppendorf: 51% conv.
10 mL vial: >97% conv.

12b
2 mL eppendorf: 74% conv.
10 mL vial: 95% conv.

Conditions: 40 mM substrate concentration, 10% DMSO v/v, 1.1 µM *Sh*AAO in 100 mM pH 6 NaPi and in the presence of catalase (2000 U/mL) at 30 ºC in an orbital shaker for 24 h. 2 mL Eppendorf tubes or 10 mL glass vials.

Scheme S3. Effect of the absence of DMSO in the *Sh*AAO-mediated oxidation of 1a and 12a.

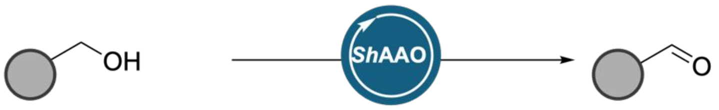

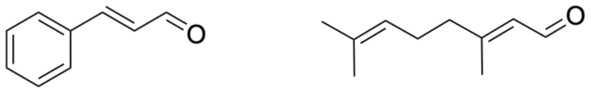

1b
97% conv. (no DMSO)
97% conv.

12b
88% conv. (no DMSO)
95% conv.

Conditions: 40 mM substrate concentration, 1.1 µM *Sh*AAO in 100 mM pH 6 NaPi and in the presence of catalase (2000 U/mL) at 30 ºC in an orbital shaker for 24 h. 10 mL vials in the presence or absence of 10% DMSO v/v.

S11. General procedure for the *one-pot* cascade process for the synthesis of chiral hydroxy ketones (*S*,*R*)-15c and (*S*,*R*)-20c.

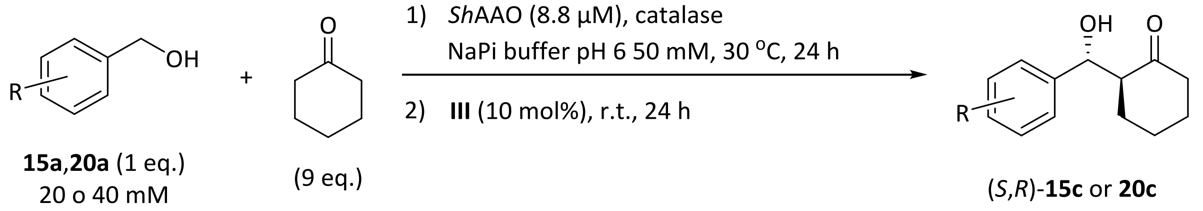

To a mixture of the corresponding alcohol **15a** or **20a** (0.1 mmol, 1 equiv.) in a 50 mL flask, catalase (2.5 or 5 mg, 1 mg mL^{-1}, 2000–5000 U mg^{-1}), 50 mM NaPi buffer pH 6 and 8.8 µM *Sh*AAO were added to reach a total volume of 2.5 or 5 mL, respectively. The reaction mixture was stirred at 30 °C in an orbital shaker for 24 h. Then, organocatalyst **III** (0.01 mmol, 10 mol%) and cyclohexanone (0.9 mmol, 9 equiv.) were added. The reaction mixture was stirred at room temperature until no starting material was detected by TLC analysis. Finally, the adducts (*S*,*R*)- (*S*,*R*)-**15c** and (*S*,*R*)-**20c** were purified by column chromatography using a mixture of *n*-hexane and ethyl acetate (8:2). (*S*,*R*)-**15c** was isolated in 99% yield (28 mg, >99:1 e.r., 20:1 d.r. antio:syn) as a pale yellow oil. (*S*,*R*)-**20c** was isolated in 50% yield (12.4 mg, 97:3 e.r., 12.5:1 d.r. antio:syn) as a pale yellow oil. Spectral data are consistent (^{1}H NMR, 300 MHz) with values previously reported in the literature.[6,7]

15c: **^{1}H NMR** (300 MHz, CDCl3) δ 7.50 – 7.43 (m, 2H), 7.23 – 7.16 (m, 2H), 4.75 (d, J = 8.7 Hz, 1H), 3.98 (s, 1H), 2.62 – 2.27 (m, 3H), 2.14 – 2.03 (m, 1H), 1.80 (dddd, J = 11.5, 4.6, 3.0, 1.7 Hz, 1H), 1.74 – 1.49 (m, 4H), 1.33 (dd, J = 12.6, 2.8 Hz, 1H). **20c**: **^{1}H NMR** (300 MHz, $CDCl_3$) δ 8.27 – 8.08 (m, 2H), 7.67 (dt, J = 7.7, 1.5 Hz, 1H), 7.52 (td, J = 7.9, 2.6 Hz, 1H), 4.90 (dd, J = 8.4, 2.6 Hz, 1H), 4.10 (d, J = 3.1 Hz, 1H), 2.62 (dddd, J = 12.7, 8.4, 5.5, 1.1 Hz, 1H), 2.54 – 2.31 (m, 2H), 2.12 (ddt, J = 11.7, 5.6, 2.8 Hz, 1H), 1.91 – 1.78 (m, 1H), 1.76 – 1.55 (m, 4H).

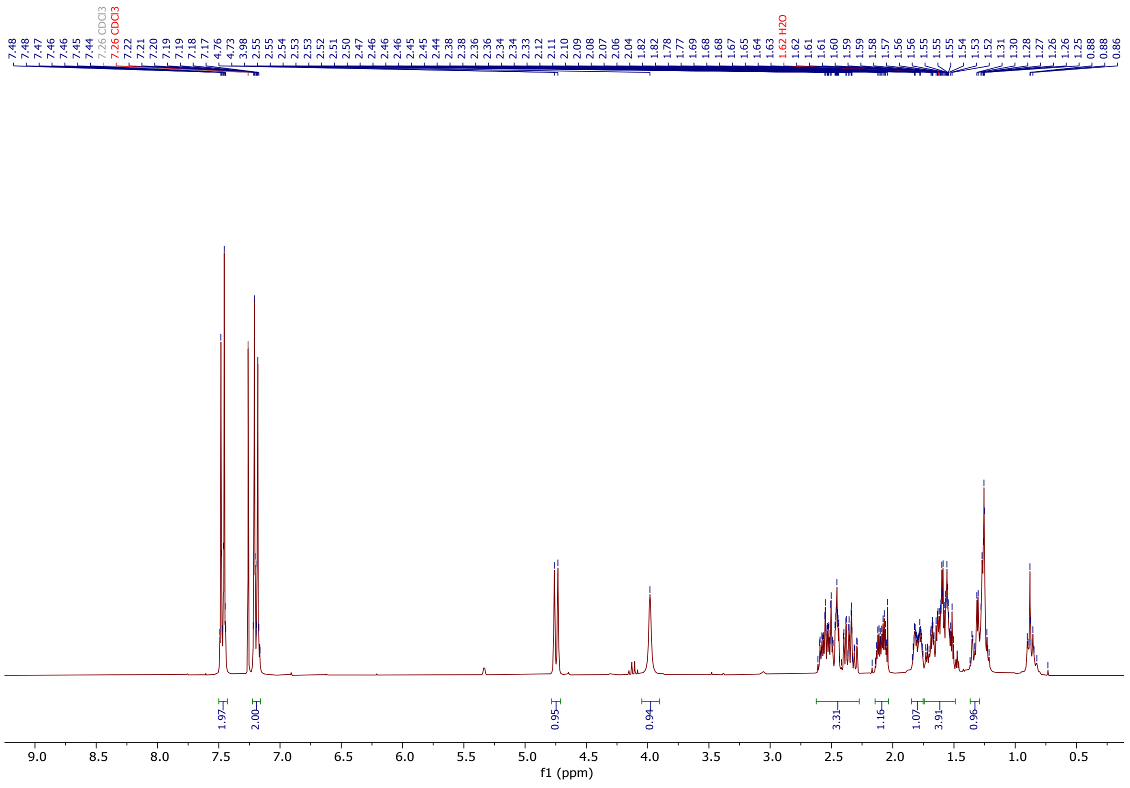

Figure S43. ^{1}H NMR spectrum of hydroxy ketone (*S*,*R*)-**15c.** (300 MHz, $CDCl_3$)

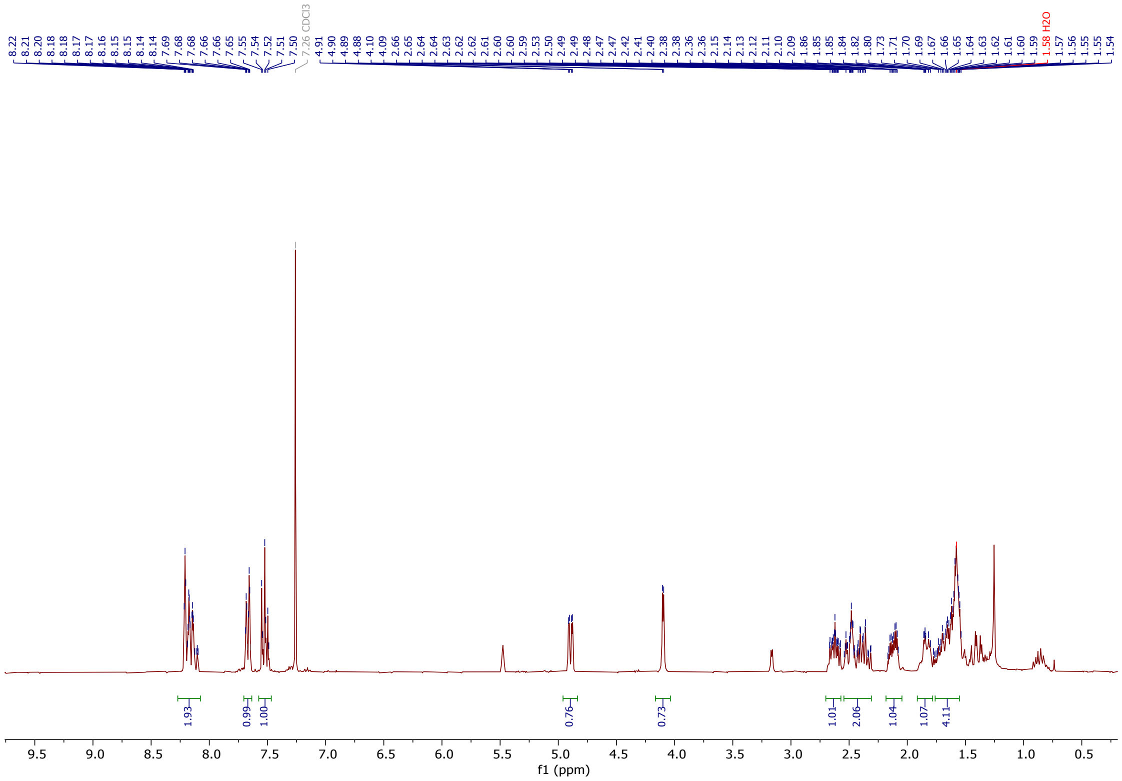

Figure S44. ^{1}H NMR spectrum of hydroxy ketone (*S*,*R*)-**20c.** (300 MHz, $CDCl_3$)

S12. General procedure for the *one-pot* cascade process for the synthesis of chiral 1,2-hydroxy phosphonate (*R*)-37c.

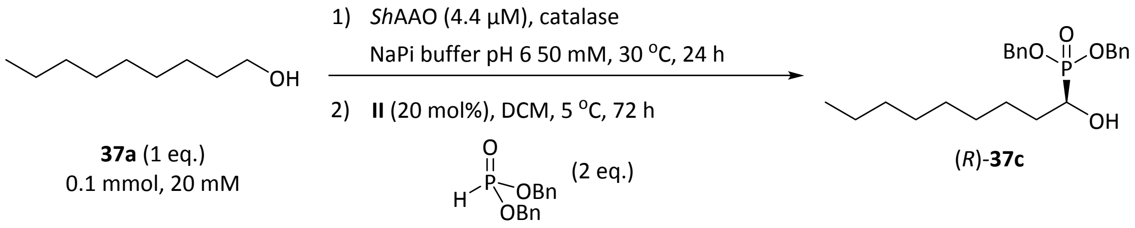

To a mixture of 1-nonanol **37a** (0.1 mmol, 1 eq.) placed in a 50 mL flask, catalase (5 mg, 1 mg mL^{-1}, 2000−5000 U mg^{-1}), 50 mM NaPi buffer pH 6 and 4.4 µM *Sh*AAO were added to a total volume of 5 mL and the reaction mixture was stirred at 30 °C in an orbital shaker for 24 h. After that time, the reaction mixture was cooled to 5 °C and organocatalyst **II** (0.02 mmol, 8.87 mg, 20 mol%), commercially available, DCM (600 µL) and dibenzyl phosphite (0.2 mmol, 46 µL, 2.00 equiv.) were added. The reaction mixture was stirred at that temperature in an orbital shaker until no starting material was detected by TLC analysis. The reaction crude was subsequently purified was purified by flash column chromatography using a mixture of *n*-hexane and ethyl acetate (6:2 to 6:4) to afford (*R*)-**37c** in 50% isolated yield (20.2 mg) and 69:31 e.r. as a yellow oil.

^{1}H NMR (300 MHz, $CDCl_3$): δ 7.42 – 7.27 (m, 10H), 5.16 – 5.00 (m, 4H), 3.90 (dt, *J* = 8.8, 4.4 Hz, 1H), 3.54 (bs, 1H), 1.83 – 1.52 (m, 3H), 1.37 – 1.19 (m, 11H), 0.95 – 0.81 (m, 3H).**^{31}P NMR** (121 MHz, $CDCl_3$) δ 26.4. **^{13}C{^{1}H}-APT NMR** (101 MHz, $CDCl_3$) δ 136.5 (d, *J* = 5.6 Hz), 128.7, 128.5, 128.1, 69.1, 68.1 (dd, *J* = 7.0, 1.7 Hz), 67.5, 32.0, 31.4, 29.4 (d, *J* = 17.5 Hz), 25.8 (d, *J* = 13.3 Hz), 22.8, 14.2. HRMS (ESI+) not obtainable.

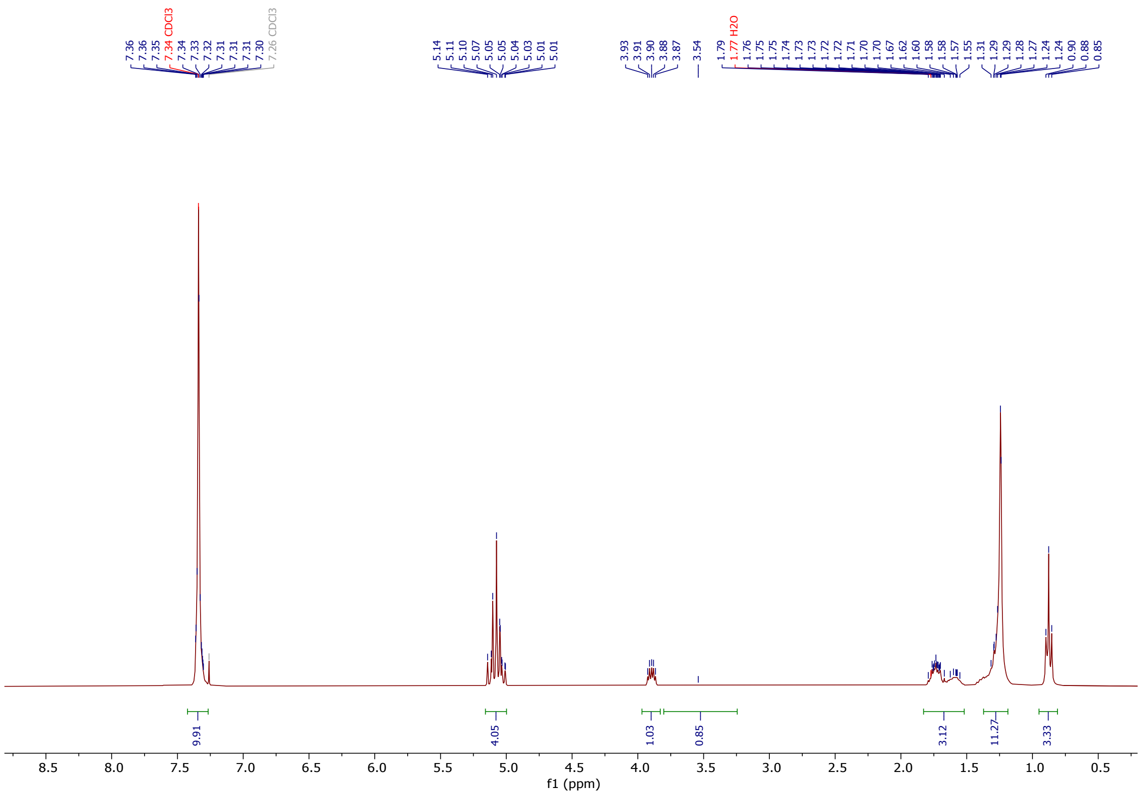

Figure S45. ^{1}H NMR spectrum of α-hydroxy phosphonate (*R*)-**37c**. (300 MHz, $CDCl_3$)

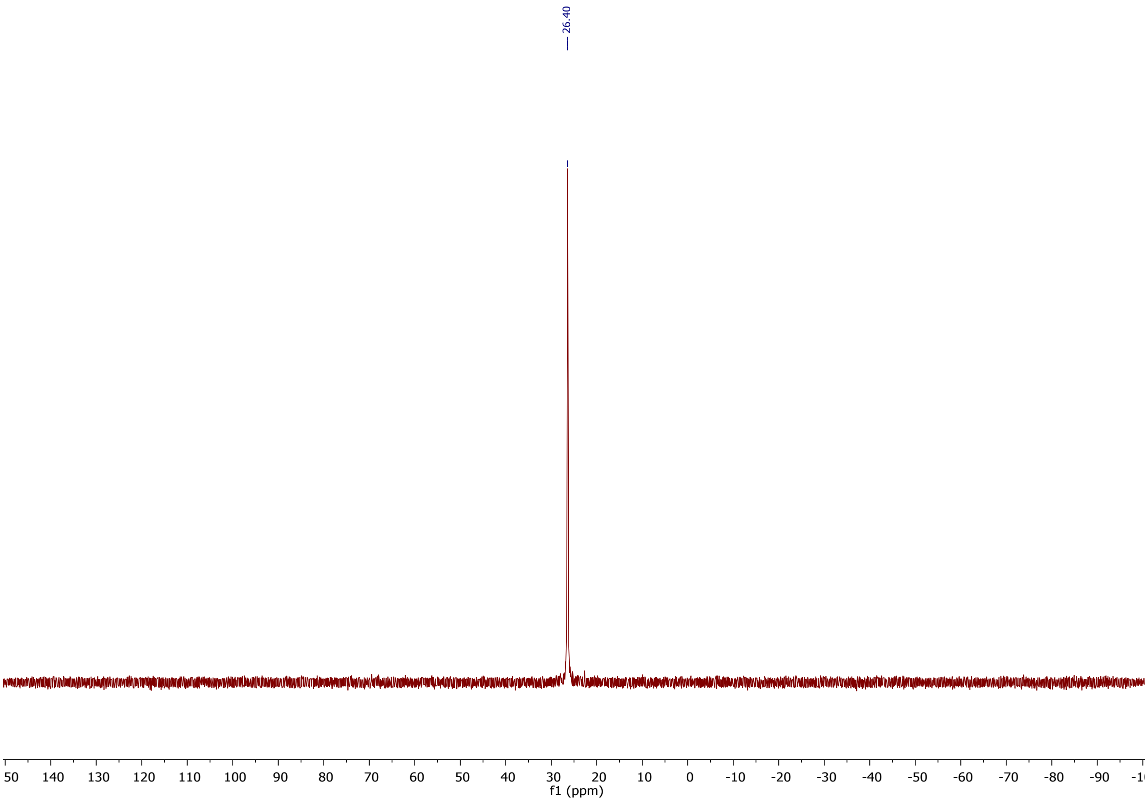

Figure S46. $^{31}P\{^{1}H\}$ NMR spectrum of α-hydroxy phosphonate (*R*)-**37c**. (300 MHz, $CDCl_3$)

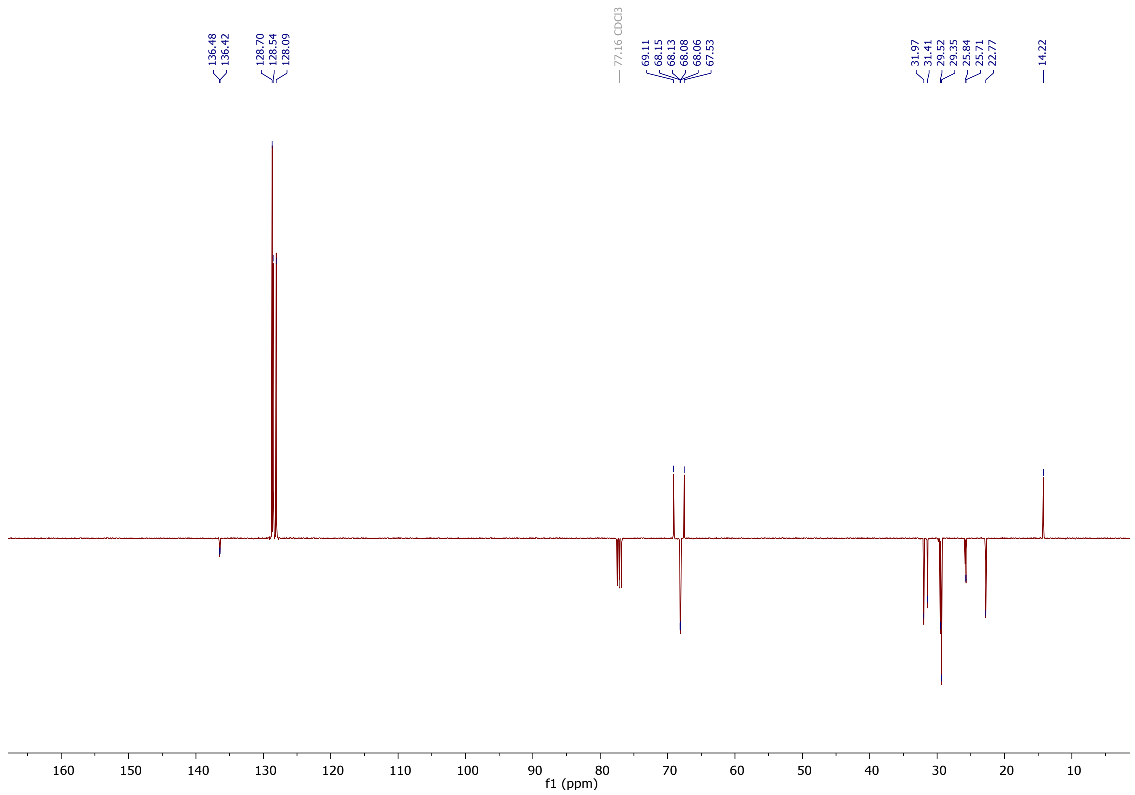

Figure S47. $^{13}C\{^1H\}$-APT NMR spectrum of α-hydroxy phosphonate (*R*)-**37c**. (300 MHz, $CDCl_3$)

S13. General procedure for the *one-pot* cascade process combining *Sh*AAO-mediated oxidation and organocatalytic asymmetric Michael additions.

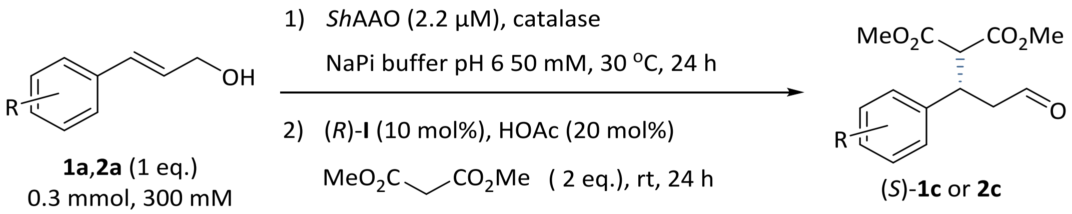

To a mixture of the corresponding alcohol **1a** or **2a** (0.3 mmol, 1 eq.) placed in a 10 mL reaction vial, catalase (1 mg, 1 mg mL^{-1}, 2000–5000 U mg^{-1}), 50 mM NaPi buffer pH 6 and and 4.4 μM *Sh*AAO were added to a total volume of 1 mL and the reaction mixture was stirred at 30 °C in an orbital shaker for 24 h. After that time, the reaction mixture was cooled to 0 °C in an ice bath and organocatalyst **I** (0.03 mmol, 10.1 mg, 10 mol%), dimethyl malonate (0.6 mmol, 73.8 μL, 2 equiv.) and acetic acid (0.06 mmol, 3.5 μL, 20 mol%) were subsequently added. The reaction mixture was stirred at that temperature for 1 hour and then at room temperature until no starting material was detected by TLC analysis. Then, the adducts (*S*)-**1c** and (*S*)-**2c** were purified by flash column chromatography using a mixture of *n*-hexane and ethyl acetate (8:2). (*S*)-**1c** was obtained in 61% isolated yield (48.3 mg, >99:1 e.r) as a yellow oil and (*S*)-**2c** was isolated in 52% yield (44 mg, 94:6 e.r.) as a yellow oil. Spectral data (^{1}H NMR, 300 MHz) are consistent with values previously reported in the literature.[2,3]

1c: **^{1}H NMR** (300 MHz, CDCl3) δ 9.61 (t, J = 1.7 Hz, 1H), 7.34 – 7.21 (m, 5H), 4.04 (ddd, J = 9.9, 8.5, 5.8 Hz, 1H), 3.77 (d, J = 6.9 Hz, 4H), 3.52 (s, 3H), 3.00 – 2.86 (m, 2H). **2c**: **^{1}H NMR** (300 MHz, $CDCl_3$) δ 9.61 (t, J = 1.7 Hz, 1H), 7.29 – 7.18 (m, 2H), 7.03 – 6.93 (m, 2H), 4.03 (td, J = 9.2, 5.2 Hz, 1H), 3.81 – 3.74 (m, 4H), 3.53 (s, 3H), 3.02 – 2.82 (m, 2H).

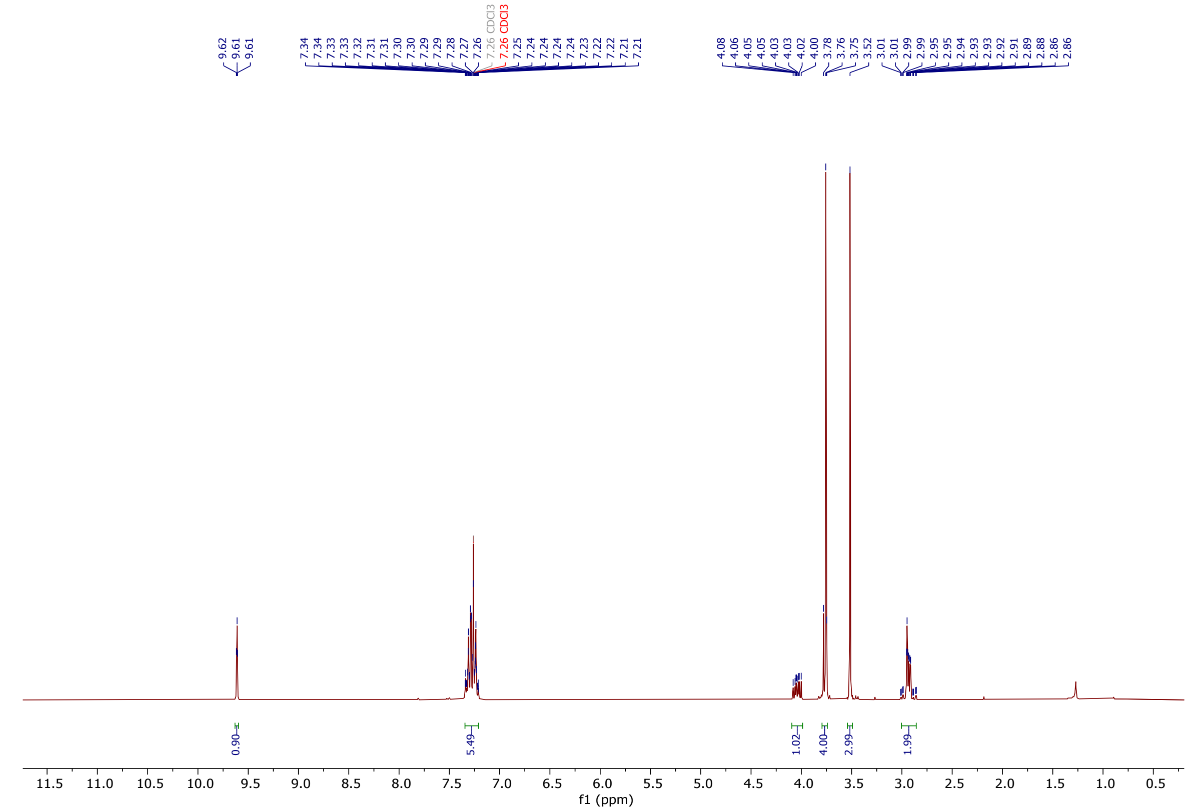

Figure S48. ^{1}H NMR spectrum of product (*S*)-**1c**. (300 Hz, $CDCl_3$).

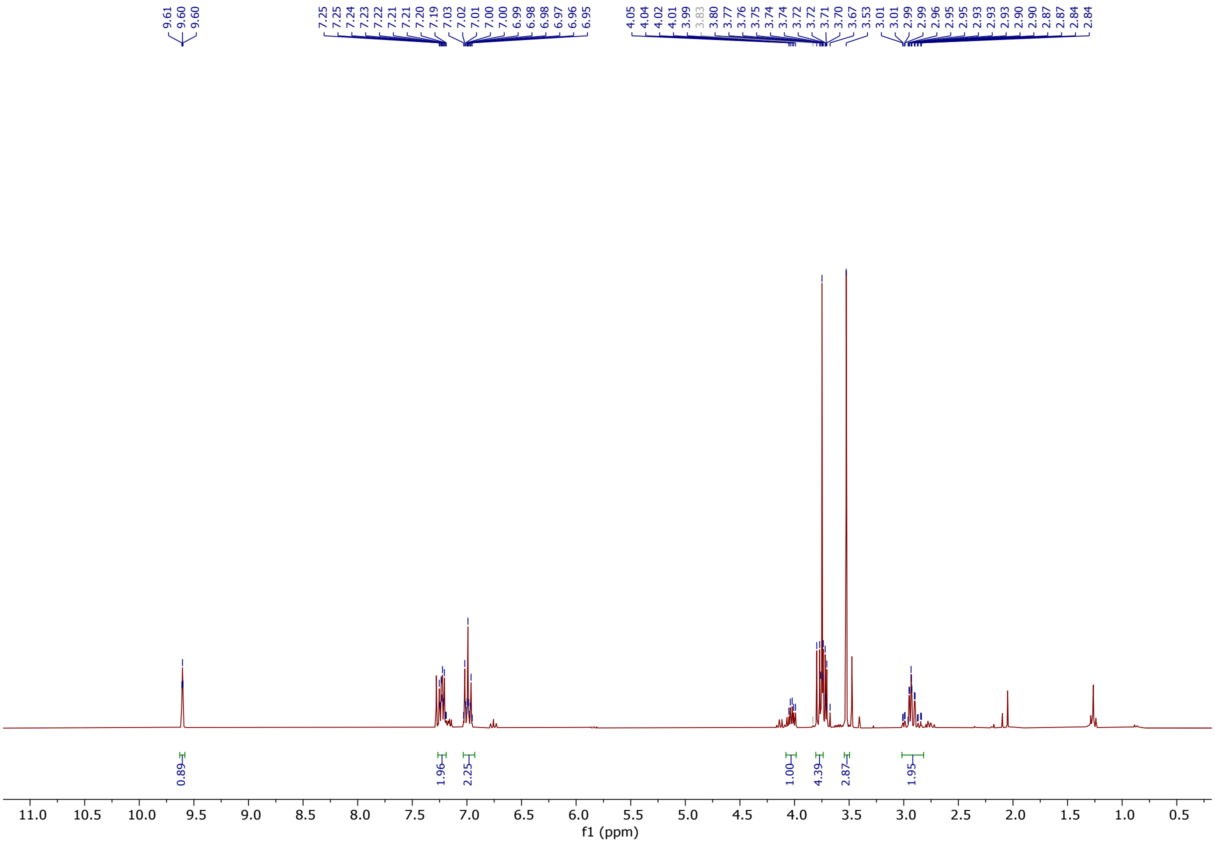

Figure S49. ^{1}H NMR spectrum of product (*S*)-**2c**. (300 Hz, $CDCl_3$).

S14. General procedure for the *one-pot* cascade process combining *Sh*AAO-mediated oxidation and Wittig olefination.

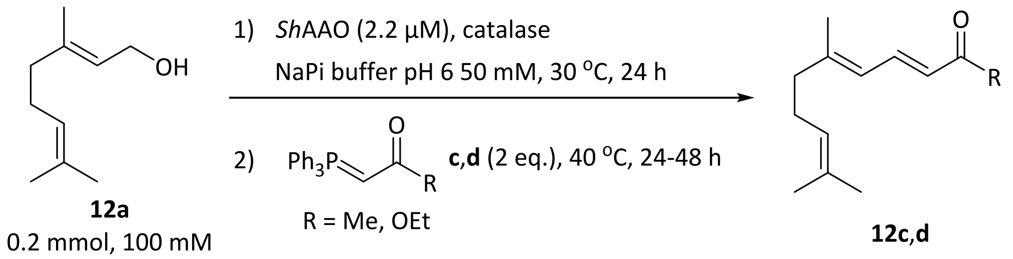

To a mixture of geraniol **12a** (0.2 mmol, 1.00 equiv.) placed in a 10 mL reaction vial, catalase (2 mg, 1 mg mL^{-1}, 2000−5000 U mg^{-1}), 50 mM NaPi buffer pH 6, and the aryl-alcohol oxidase from *Streptomyces hiroshimensis* (*Sh*AAO) (4.4 µM) were added to a total volume of 2 mL and the reaction mixture was stirred at 30 °C in an orbital shaker for 24 h. After that time, the corresponding phosphorous ylide **c** (R:Me) or **d** (R:OEt) (0.4 mmol, 2 equiv.) was added and the reaction mixture was stirred at 40 °C in an oil bath for 24 or 48 h, respectively. Then, the adducts **12c** and **12d** were purified by flash column chromatography using a mixture of *n*-hexane and ethyl acetate (8:2). **12c** was isolated in 62% yield (23.8 mg, 96:4 *E/Z*) as a clear oil and **12d** was isolated in 97% yield (43.1 mg, 87/13 *E/Z*) as a yellow oil. Spectral data (^{1}H NMR, 300 MHz) are consistent with values previously reported in the literature.[4,5]

12c: **^{1}H NMR** (300 MHz, $CDCl_3$) δ 7.43 (dd, J = 15.3, 11.4 Hz, 1H), 6.08 (d, J = 15.3 Hz, 1H), 6.00 (d, J = 11.3 Hz, 1H), 5.14 – 5.01 (m, 1H), 2.27 (s, 3H), 2.16 (d, J = 3.3 Hz, 3H), 1.91 (d, J = 1.3 Hz, 3H), 1.68 (s, 3H), 1.61 (s, 3H). **12d**: **^{1}H NMR** (300 MHz, $CDCl_3$) δ 7.43 (dd, J = 15.3, 11.4 Hz, 1H), 6.08 (d, J = 15.3 Hz, 1H), 6.00 (d, J = 11.3 Hz, 1H), 5.14 – 5.01 (m, 1H), 2.27 (s, 3H), 2.19 – 2.14 (m, 3H), 1.91 (s, 3H), 1.68 (s, 3H), 1.61 (s, 3H).

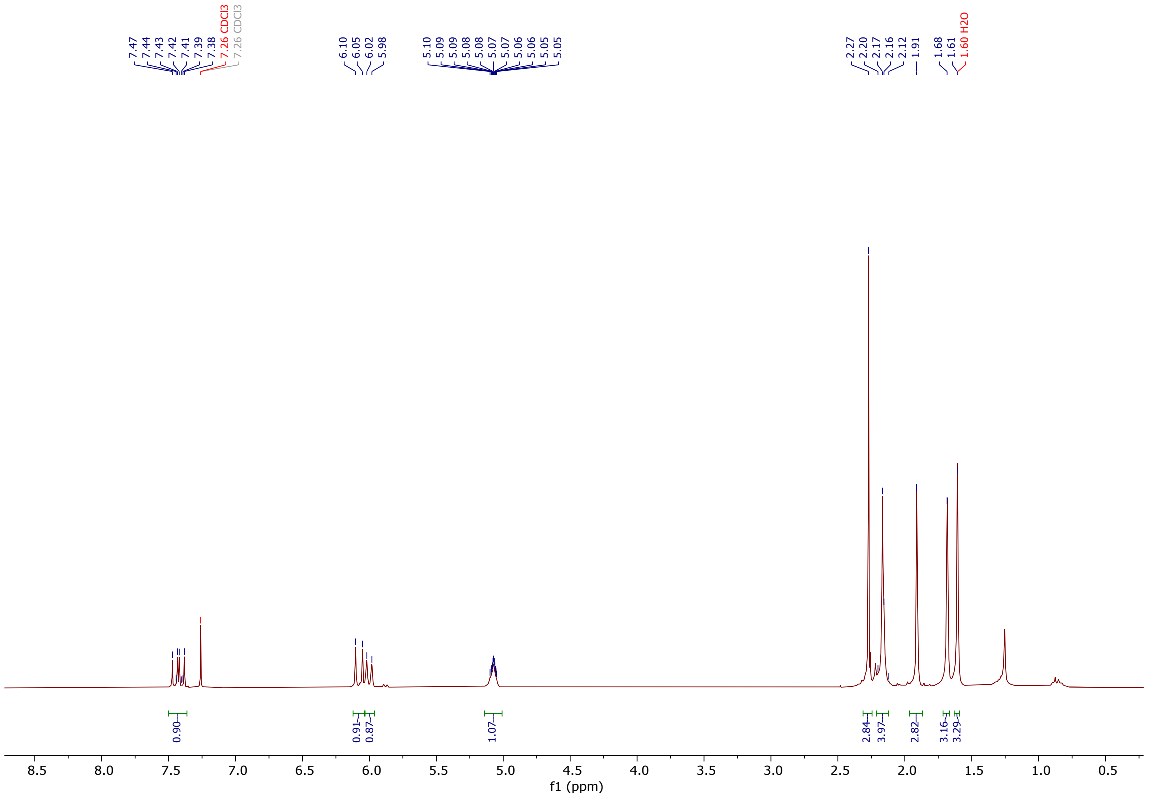

Figure S50. ^{1}H NMR spectrum of product **12c**. (300 Hz, $CDCl_3$).

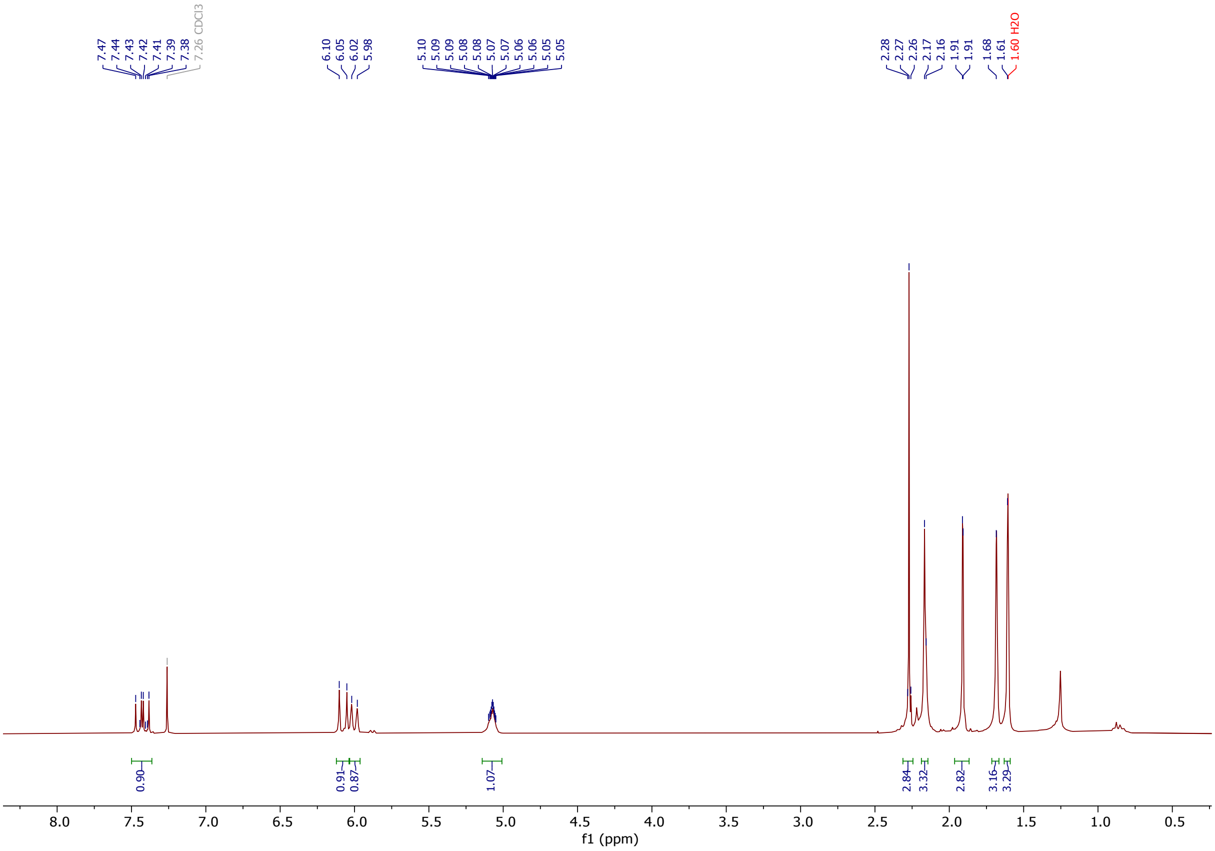

Figure S51. ^{1}H NMR spectrum of product **12d**. (300 Hz, $CDCl_3$).

S15. Chiral HPLC analysis of compounds 15c, 20c and 37c

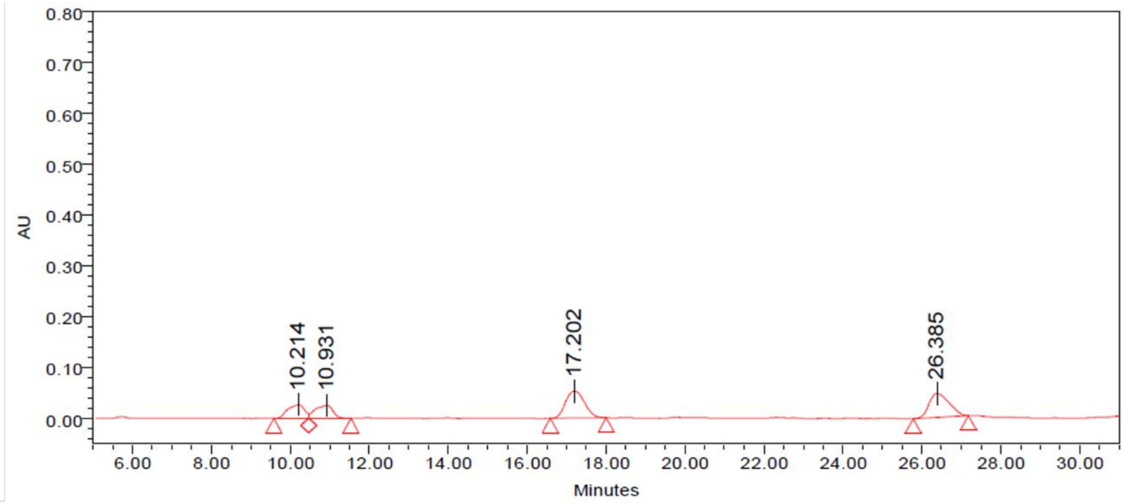

Processed Channel: PDA 251.2 nm

	Processed Channel	Retention Time (min)	Area	% Area	Height
1	PDA 251.2 nm	10.214	779954	15.62	26526
2	PDA 251.2 nm	10.931	801700	16.05	25449
3	PDA 251.2 nm	17.202	1760658	35.25	52683
4	PDA 251.2 nm	26.385	1651847	33.08	46716

Figure S52. Racemic mixture of **15c**. Daicel ChiralPak IC column (*n*-hexane/ethyl acetate = 90:10, 1 mL min^{-1}, λ = 251.2 nm).

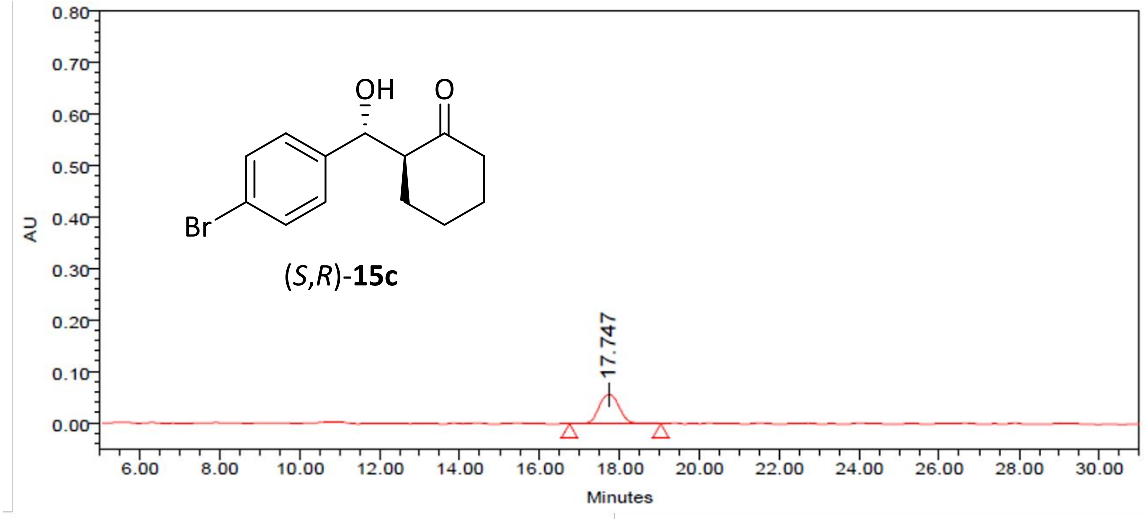

Processed Channel: PDA 251.2 nm

	Processed Channel	Retention Time (min)	Area	% Area	Height
1	PDA 251.2 nm	17.747	1981079	100.00	56724

Figure S53. Enantioenriched mixture of (*S*,*R*)-**15c** (>99:1 d.r. and >99:1 e.r.). Daicel ChiralPak IC column (*n*-hexane/ethyl acetate = 90:10, 1 mL min^{-1}, λ = 251.2 nm).

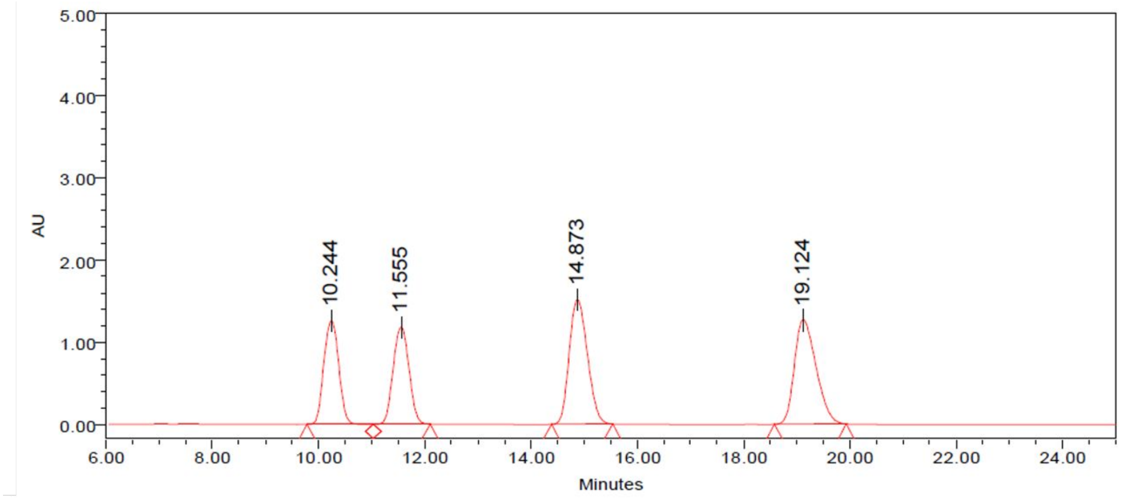

Processed Channel: PDA 276.3 nm

	Processed Channel	Retention Time (min)	Area	% Area	Height
1	PDA 276.3 nm	10.244	24599782	20.31	1257111
2	PDA 276.3 nm	11.555	24490208	20.22	1181174
3	PDA 276.3 nm	14.873	36060566	29.77	1515438
4	PDA 276.3 nm	19.124	35997383	29.71	1269589

Figure S54. Racemic mixture of **20c**. Daicel ChiralPak IC column (*n*-hexane/ethyl acetate = 85:15, 1 mL min^{-1}, λ = 276.3 nm).

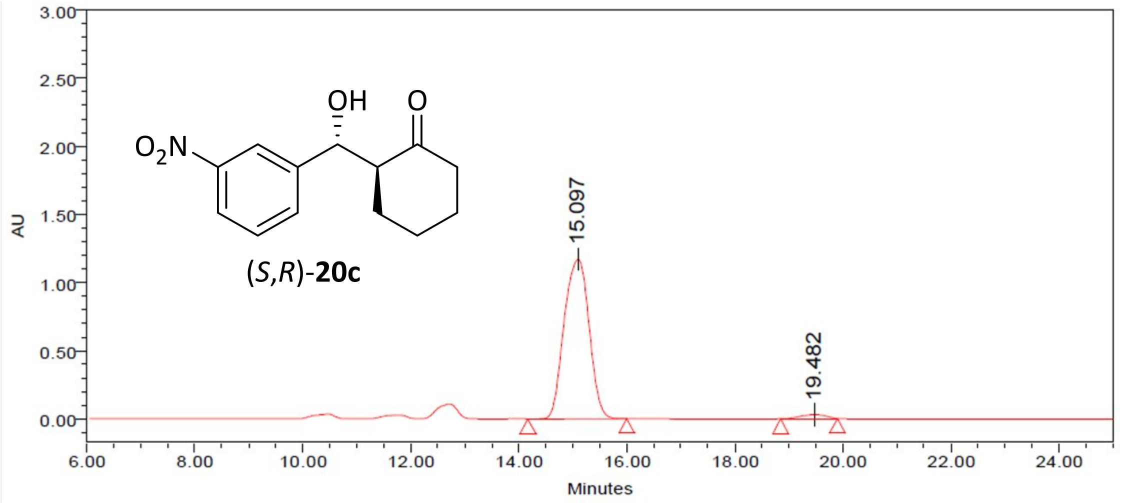

Processed Channel: PDA 276.3 nm

	Processed Channel	Retention Time (min)	Area	% Area	Height
1	PDA 276.3 nm	15.097	37682271	97.52	1172206
2	PDA 276.3 nm	19.482	959957	2.48	28946

Figure S55. Enantioenriched mixture of (*S*,*R*)-**20c** (98:2 e.r.). Daicel ChiralPak IC column (*n*-hexane/ethyl acetate = 85:15, 1 mL min^{-1}, λ = 276.3 nm).

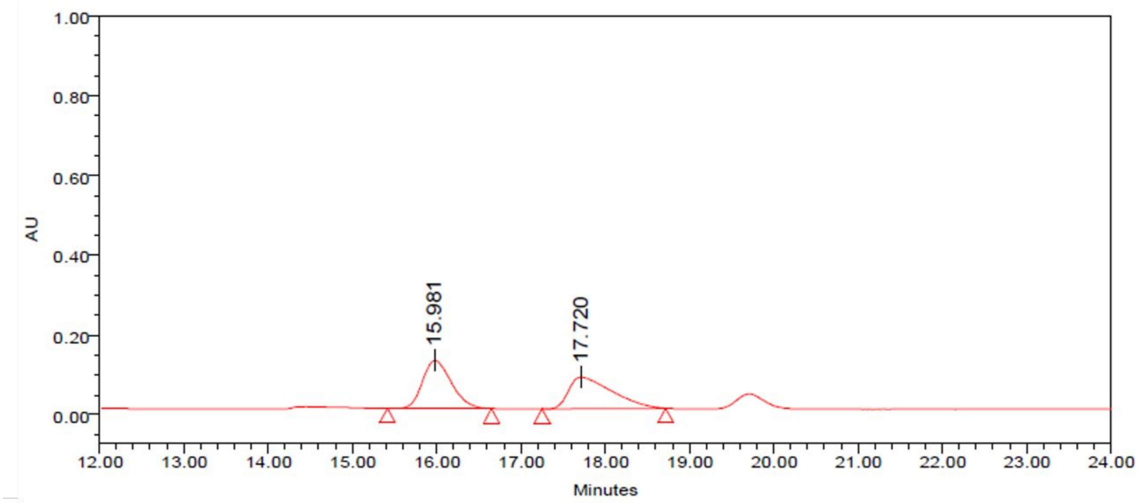

Processed Channel: PDA 219.9 nm

	Processed Channel	Retention Time (min)	Area	% Area	Height
1	PDA 219.9 nm	15.981	2866281	50.40	120823
2	PDA 219.9 nm	17.720	2821070	49.60	78909

Figure S56. Racemic mixture of **37c**. Daicel ChiralPak IA column (*n*-hexane/isopropyl alcohol = 95:5, 1 mL min^{-1}, λ = 219.9 nm).

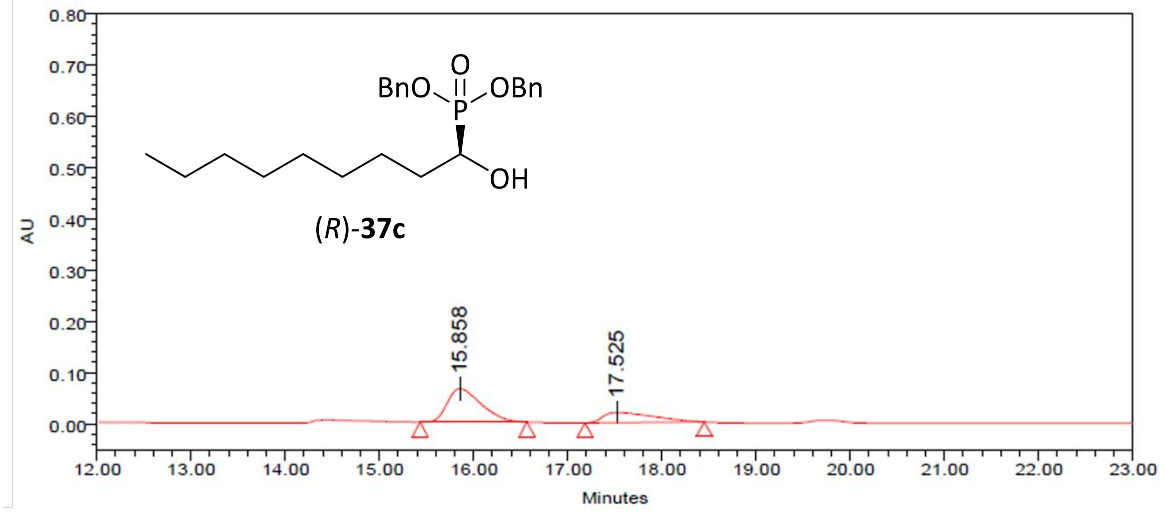

Processed Channel: PDA 223.3 nm

	Processed Channel	Retention Time (min)	Area	% Area	Height
1	PDA 223.3 nm	15.858	1637792	68.76	65345
2	PDA 223.3 nm	17.525	744265	31.24	19949

Figure S57. Enantioenriched mixture of (*R*)-**37c** (69:31 e.r.). Daicel ChiralPak IA column (*n*-hexane/isopropyl alcohol = 95:5, 1 mL min^{-1}, λ = 223.3 nm).

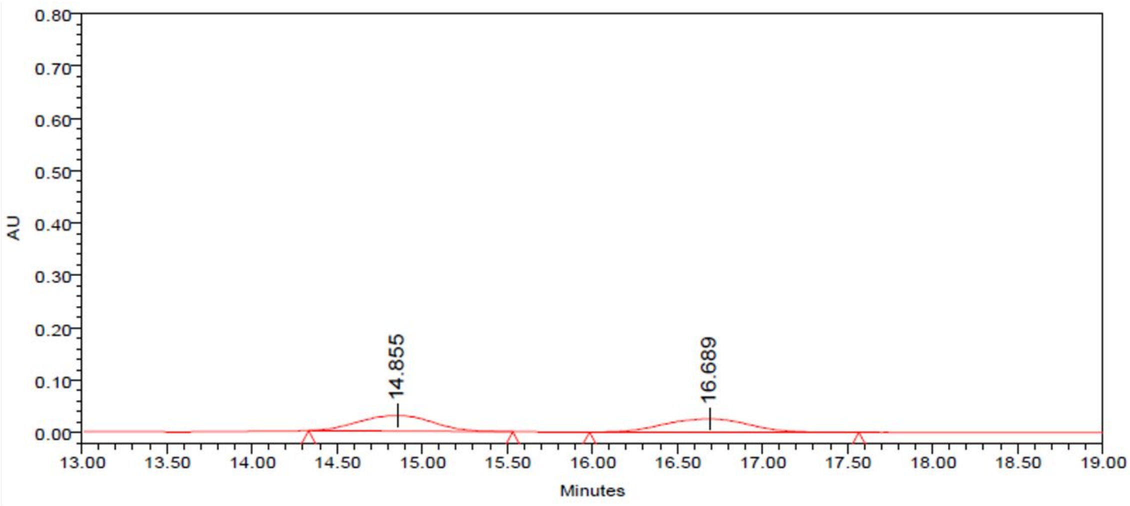

Processed Channel: PDA 234.9 nm

	Processed Channel	Retention Time (min)	Area	% Area	Height
1	PDA 234.9 nm	14.855	892651	50.52	29661
2	PDA 234.9 nm	16.689	874175	49.48	25311

Figure S58. Racemic mixture of **1c**. Daicel ChiralPak IA column (*n*-hexane/isopropyl alcohol = 90:10, 1 mL min^{-1}, λ = 234.9 nm).

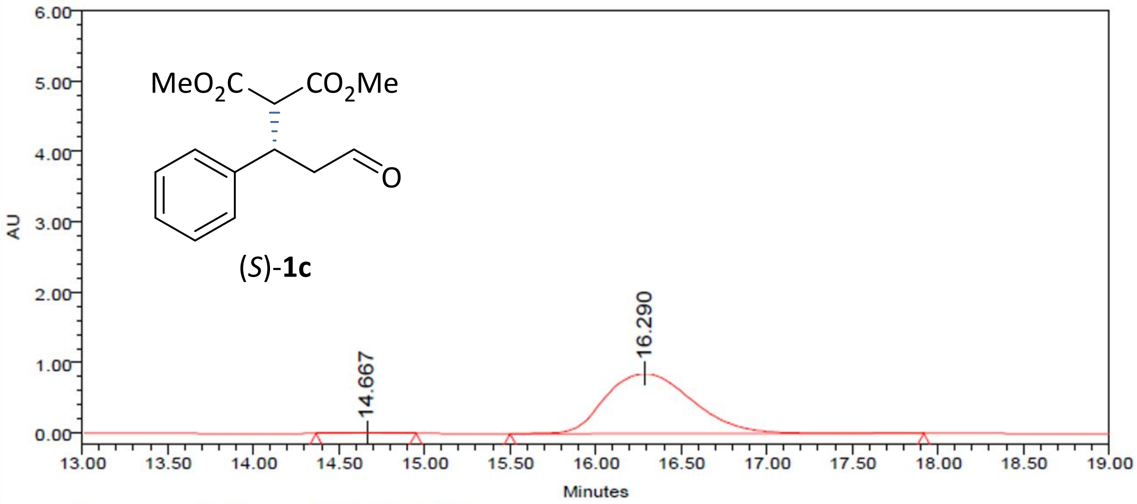

Processed Channel: PDA 234.9 nm

	Processed Channel	Retention Time (min)	Area	% Area	Height
1	PDA 234.9 nm	14.667	150743	0.50	6985
2	PDA 234.9 nm	16.290	30132587	99.50	849517

Figure S59. Enantioenriched mixture of (*S*)-**1c** (>99:1 e.r.). Daicel ChiralPak IA column (*n*-hexane/isopropyl alcohol = 90:10, 1 mL min^{-1}, λ = 234.9 nm).

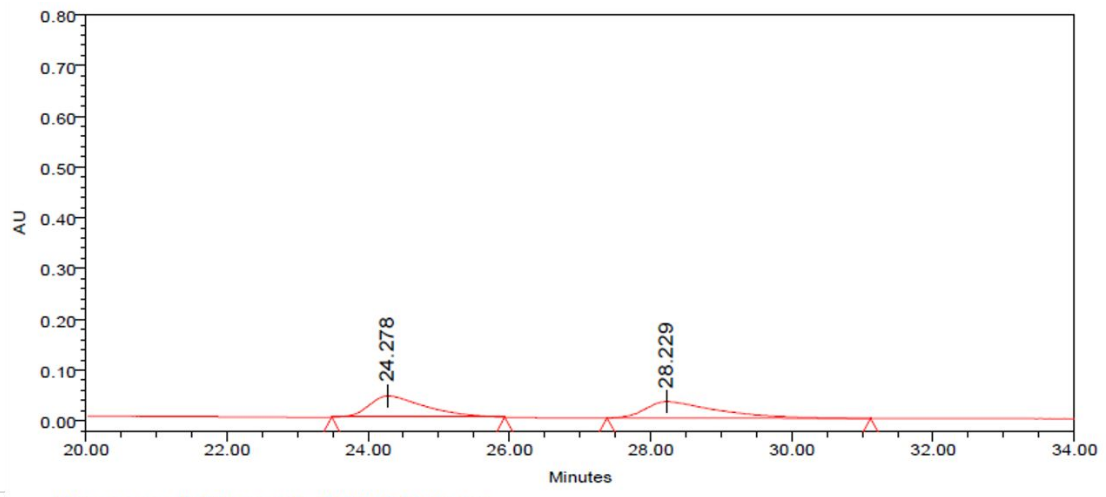

Processed Channel: PDA 249.9 nm

	Processed Channel	Retention Time (min)	Area	% Area	Height
1	PDA 249.9 nm	24.278	2298236	50.45	41703
2	PDA 249.9 nm	28.229	2257290	49.55	32417

Figure S60. Racemic mixture of **2c**. Phenomenex i-Amylose-1 column (*n*-hexane/isopropyl alcohol = 95:5, 1 mL min^{-1}, λ = 249.9 nm).

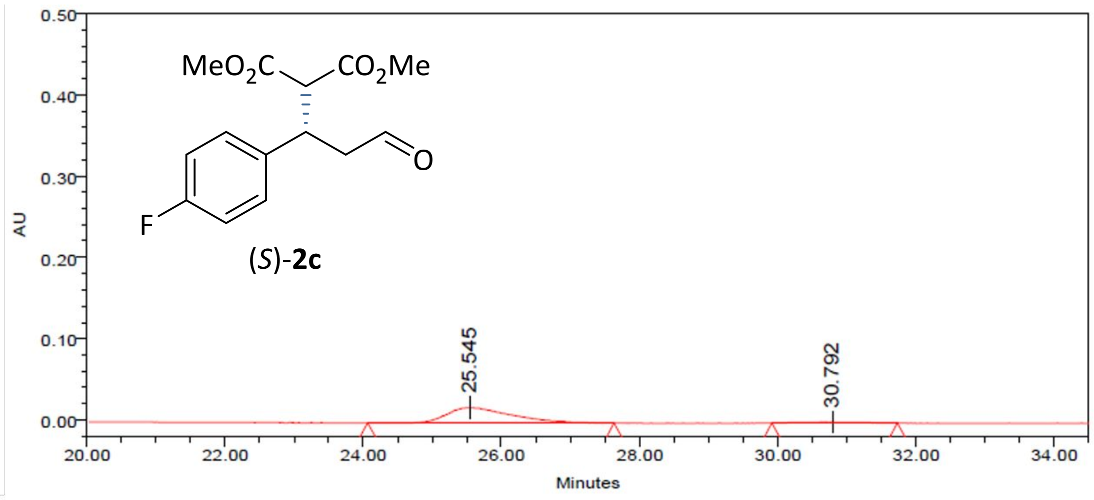

Processed Channel: PDA 249.9 nm

	Processed Channel	Retention Time (min)	Area	% Area	Height
1	PDA 249.9 nm	25.545	1218948	94.72	18767
2	PDA 249.9 nm	30.792	67967	5.28	1141

Figure S61. Enantioenriched mixture of (*S*)-**2c** (95:5 e.r.). Phenomenex i-Amylose-1 column (*n*-hexane/isopropyl alcohol = 95:5, 1 mL min^{-1}, λ = 249.9 nm).

S16. Preparative-scale biotransformations.

S16.1. Process optimization.

The oxidation of cinnamyl alcohol **1a** was used as a model reaction to investigate the best conditions for preparative-scale reactions. Reactions were run using standard *Sh*AAO-mediated oxidations with varying substrate concentrations and enzyme loadings.

Table S1. Conversions and turnover numbers at different substrate concentrations and enzyme loadings in the *Sh*AAO-mediated oxidation of 1a.

Entry	[substrate] (mM)	*Sh*AAO (µM)	Conversion (%)	TN
**1**	40	1.1	90	32727
**2**	80	1.1	48	34909
**3**	120	1.1	48	52364
**4**	120	-	<3	-
**5**	120	1.1	13	14182
**6**	120	2.2	25	13636
**7**	120	4.4	>97	27907
**8**	240	4.4	92	51349
**9**	500	4.4	44 ± 6	51163

Conditions: 1 mL reaction volume, 10% DMSO v/v, 2000 U catalase in 100 mM NaPi pH 6 buffer at 30 ºC and 24 h. Conversions determined by ^{1}H-NMR over the reaction crudes. Turnover numbers (TN) defined as mmol of product formed per mmol of catalyst.

Table S2. Conversions and turnover frequencies in the *Sh*AAO-mediated oxidation of 1a at different reaction times.

Entry	Time (min)	Conversion (%)	TOF (min^{-1})
**1**	o.n	44 ± 6	---
**2**	20	6	349
**3**	40	12	334
**4**	60	17	329
**5**	90	21	271
**6**	120	24	233
**7**	180	35	226
**8**	270	39	168
**9**	360	44	142

Conditions: 500 mM **1a** (67 mg), 4.4 µM *Sh*AAO, 10% DMSO v/v, 2000 U catalase in 100 mM NaPi pH 6 buffer at 30 ºC.Conversions determined by ^{1}H-NMR over the reaction crudes. Turnover frequency (TOF) defined as mmol of product formed per mmol of catalyst per unit of time.

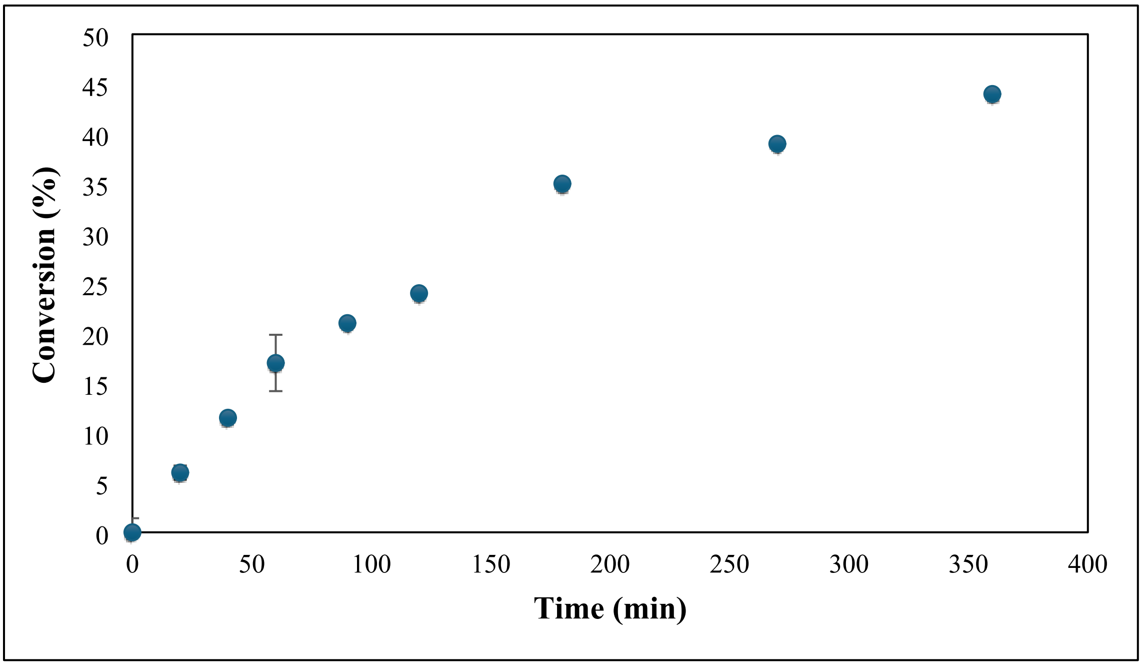

Figure S62. Time-course of the *Sh*AAO-mediated aerobic oxidation of **1a**. Conditions: 500 mM **1a** (67 mg), 4.4 µM *Sh*AAO, 10% DMSO v/v, 2000 U catalase in 100 mM NaPi pH 6 buffer at 30 ºC.

Table S3. Effect of the vessel and atmosphere in the *Sh*AAO-mediated oxidation of 1a.

Entry	Vessel	Atmosphere	Conversion (%)	TN
**1**	10 mL Erlenmeyer	Air	44 ± 6	51163
**2**	10 mL Erlenmeyer	O_2	54	62791
**3**	25 mL Erlenmeyer	Air	45 ± 9	52326
**4**	25 mL Erlenmeyer	O_2	38 ± 4	44186

Conditions: 500 mM **1a** (67 mg), 4.4 µM *Sh*AAO, 10% DMSO v/v, 2000 U catalase in 100 mM NaPi pH 6 buffer at 30 ºC. Conversions determined by ^{1}H-NMR over the reaction crudes. Turnover numbers defined as mmol of product formed per mmol of catalyst.

Table S4. Study of catalase loading and addition strategy in the *Sh*AAO-mediated oxidation of 1a.

Entry	Catalase (U)	Catalase Additions	Conversion (%)	TN
**1**	8000	1 (t_0)	44 ± 6	51163
**2**	16000	1 (t_0)	46 ± 14	53488
**3**	16000	2 (t_0, t_3)	57 ± 9	64390
**4**	24000	3 (t_0, t_2, t_4)	35 ± 2	40698
**5**	32000	4 (t_0, t_2, t_4, t_6)	59 ± 2	68605

Conditions: 500 mM **1a** (67 mg), 4.4 µM *Sh*AAO, 10% DMSO v/v in 100 mM NaPi pH 6 buffer at 30 ºC.Conversions determined by ^{1}H-NMR over the reaction crudes. Turnover numbers defined as mmol of product formed per mmol of catalyst.

S16.2. Gram-scale biooxidation procedure

Scale-up of cinnamaldehyde production was conducted in two 100 mL baffled Erlenmeyer flasks containing cinnamyl alcohol **1a** (500 mM, 5 mmol, 670 mg), DMSO (1 mL), 100 mM NaPi buffer pH 6 (9 mL), catalase (8000 U/mL), and *Sh*AAO, in a total reaction volume of 10 mL. The reaction was conducted overnight at 250 rpm and 30 ºC in an orbital shaker. Then, the reaction mixtures were combined and extracted with EtOAc (3 × 25 mL). The organic layers were combined, dried over Mg_2SO_4, filtered, and the solvent was removed under reduced pressure. The reaction crude was by column chromatography using an 8:2 mixture of *n*-hexane and EtOAc to afford 958 mg of cinnamaldehyde **2a** as a yellow oil (75% isolated yield).

Table S5. Gram-scale biotransformations using different purified *Sh*AAO and cell-free lysate.

Entry	[substrate] (mM)	*Sh*AAO	Conversion (%)	Yield (%)	TN	Productivity
**1**	500 (2x 670 mg)	4.4 µM (purified enzyme)	41.5	33 (422 mg)	48256	-
**2**	500 (2x 670 mg)	2 mg/mL (CFE)	60.5	53 (684 mg)	-	17.1
**3**	500 (2x 670 mg)	4 mg/mL (CFE)	92.5	75 (958 mg)	-	11.9

Scale up was conducted in two 100 mL baffled Erlenmeyer with 10 mL reaction volume each. Conversions determined by ^{1}H-NMR over the reaction crudes. Isolated yield determined after column chromatography. Turnover numbers defined as mmol of product formed per mmol of catalyst. Productivity defined as mg of product formed per mg of catalyst.

S17. Enzyme immobilisation

S17.1. Enzyme immobilisation procedure

EziG Carriers (Opal, Coral and Amber): 50 mg of the desired EziG carriers were washed twice with washing buffer (200 mM KPi, pH 6). After washing, a 1 mL solution of a known concentration of purified enzyme was mixed with the carrier on an orbital shaker at 4 ºC for 2 hours. The mixture was then centrifuged (20627 x *g*, 1 minute), and the supernatant was collected and analysed with a Nanodrop1000 to determine the concentration of remaining enzyme.

Purolite Lifetech ECR8309F: 50 mg of carrier was washed twice with washing buffer and then incubated at room temperature with 0.5 mL 2% glutaraldehyde solution for one hour. After further washing with washing buffer, the carrier was incubated with purified enzyme at 4 ºC for 20 hours. The mixture was then centrifuged (20627 x *g*, 1 minute), and the supernatant was collected and analysed with a Nanodrop1000 to determine the concentration of remaining enzyme. For scaled up batch reactions *Sh*AAO was immobilised on 150 mg ECR8309F.

Sunresin EMC 7025: 50 mg of carrier was washed twice with washing buffer. After washing, the carrier was incubated with purified enzyme at 4 ºC for 20 hours. This was then left to stand for 24 hours at 4 ºC. The mixture was then centrifuged (20627 x *g*, 1 minute), and the supernatant was collected and analysed with a Nanodrop1000 to determine the concentration of remaining enzyme.

The mass of enzyme immobilised on each carrier was calculated using equation 1:

Equation 1: Mass enzyme immobilised (mg) = mass offered (mg) - mass remaining (mg)

S17.2. Initial analytical scale immobilised enzyme biooxidation procedure

Initial activity testing of the immobilised preparations was carried out in 1.6 mL Eppendorf tubes. Cinnamyl alcohol (40 mM), DMSO (0.1 mL), catalase (2000 U/mL), and immobilised *Sh*AAO were added to reaction buffer (100 mM KPi buffer pH 6) up to a final volume of 1 mL. The reaction was shaken at 250 rpm for 90 minutes at 30 ºC. Reactions were then centrifuged (5000 x *g*) and the supernatant was removed and stored at 4 ºC for analysis, while the immobilised biocatalyst was retained for subsequent reaction cycles. Two additional reactions cycles were set up as described previously. Conversions were determined by analysis of ^{1}H NMR spectrum after extraction into $CDCl_3$.

S17.3. Scaled immobilised enzyme biooxidation procedure

Reaction in flasks: *Sh*AAO was immobilised on 200 mg of ECR8309F (1 wt%) and the reaction was run in either 100 mL baffled flasks or 100 mL Erlenmeyer flasks. Cinnamyl alcohol (670 mg, 5 mmol), catalase (8000 U mL^{-1}), and immobilised *Sh*AAO were added to reaction buffer (100 mM KPi, pH 6, 10% v/v DMSO) up to a total reaction volume of 10 mL. The reaction was shaken at 250 rpm for 24 hours and 30 ºC in an orbital shaker. Reactions were then centrifuged (5000 x *g*) and the supernatant removed and stored at 4 ºC for analysis, while the immobilised biocatalyst pellet was retained for subsequent reaction cycles. Conversions were determined by analysis of the ^{1}H NMR spectrum after extraction into $CDCl_3$.

Reaction in Falcon tubes: *Sh*AAO was immobilised on 150 mg of ECR8309F (1 wt%) and the reaction was run in a 50 mL Falcon tube. Cinnamyl alcohol (134 mg, 1 mmol), catalase (2000 U mL^{-1}), and immobilised *Sh*AAO were added to reaction buffer (100 mM KPi, pH 6, 10% v/v DMSO) up to a reaction volume of 25 mL. The reaction was shaken at 250 rpm for 24 hours at 30 ºC in an orbital

shaker. Reactions were then centrifuged (5000 x *g*) and the supernatant removed and stored at 4 ºC for analysis, while the immobilised biocatalyst pellet was retained for subsequent reaction cycles. Conversions were determined by analysis of the ^{1}H NMR spectrum after extraction into $CDCl_3$.

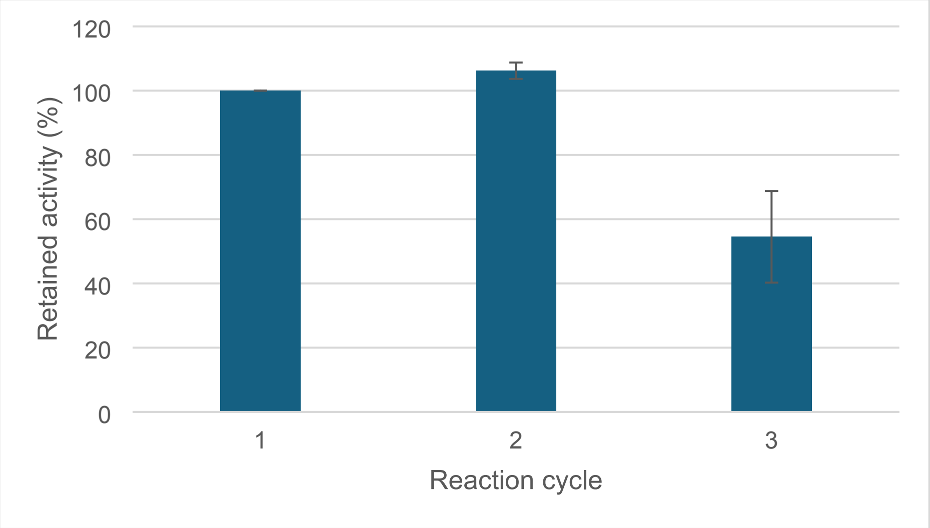

Figure S55. Scale up study of immobilised *Sh*AAO on 150 mg ECR8309F with 40 mM **1a** (25 mL reaction volume) and 2000 U/mL catalase. Initial conversion was 81%

Table S6. Immobilisation data for carriers screened in analytical scale initial activity and retained activity studies

Carrier	Enzyme offered (mg)	Enzyme immobilised (mg)	Immobilisation yield (%)	w/w %
**EMC 7025**	1.51	0.58	38.10	1.1
**Amber**	1.54	1.16	75.32	2.3
**Coral**	1.54	1.12	72.73	2.2
**Opal**	1.54	0.56	36.04	1.1
**ECR8309F**	1.54	0.59	37.99	1.2

Table S7. Scale up studies of immobilised *Sh*AAO on ECR8309F with [a] 0.5 M 1a and 8000 U/mL catalase or [b] 40 mM 1a and 2000 U/mL catalase.

Type	Mass resin (mg)	Enzyme offered (mg)	Enzyme immobilised (mg)	Immobilisation yield (%)	wt %	Conv	Initial TN	TTN
**100 mL Baffled flasks[a]**	201.0	2.5	2.0	80	1.0	12.5	17002	17002
**100 mL Erlenmeyer[a]**	199.5	2.5	2.3	92	1.2	29.4	34849	34849
**50 mL Falcon Tubes[a]**	199.5	2.5	2.1	84	1.1	13	17398	17398
**50 mL Falcon Tubes[b]**	150.0	2.2	1.7	77	1.2	81	25836	67267
